# Diversification of diterpene biosynthesis occurred early in octocoral evolution

**DOI:** 10.1073/pnas.2520279122

**Published:** 2025-11-26

**Authors:** Immo Burkhardt, Hannah K. Bone, Natalie E. Grayson, Helena A. Leucke, Johanna Gutleben, Paul R. Jensen, Andrea M. Quattrini, Alexander B. Chase, Bradley S. Moore

**Affiliations:** ^a^Scripps Institution of Oceanography, University of California San Diego, La Jolla, CA 92093; ^b^Department of Invertebrate Zoology, Smithsonian National Museum of Natural History, Washington, DC 20560; ^c^Roy M. Huffington Department of Earth Sciences, Southern Methodist University, Dallas, TX 75205; ^d^Skaggs School of Pharmacy and Pharmaceutical Sciences, University of California San Diego, La Jolla, CA 92093

**Keywords:** terpene cyclases, octocorals, evolution, biosynthesis, deep sea

## Abstract

Octocorals are the major source of marine-derived bioactive terpenoids. However, the vast majority of explored chemistry is known from shallow-water species, leaving deep-sea octocorals largely unexplored. Recent genomic work uncovered terpene biosynthetic pathways encoded in octocoral genomes, enabling deeper investigation into the evolution and ecological distribution of these compounds. Here, we collected nine deep-sea octocoral specimens representing both taxonomic orders and profiled their terpenoid chemistry. These samples revealed extensive diversity in sesquiterpene scaffolds, along with repeated detection of five widespread structural families of diterpenes (xeniaphyllene-, cembrene B-, elisabethatriene-, cembrene A-, and klysimplexin R; collectively, “XBECK-type” diterpenoids). Phylogenetic analysis of terpene cyclase (TC) sequences from these samples, combined with publicly available sequences and functional characterization of selected genes, indicated that most TC genes encode functionally diverse sesquiterpene cyclases that corresponded to deeply rooted taxonomic origins, rather than biochemical function. We further identified five monophyletic clades, each comprising isofunctional enzymes that produce precursors for distinct XBECK-type diterpenoids and showing broad representation across octocoral taxa. These findings suggest that diterpenoid biosynthesis evolved early in coral evolution, with the last common ancestor already possessing multiple functionally distinct TC enzymes. Our results establish terpenoid production as an ancient, central trait of octocorals, irrespective of habitat, thereby highlighting the tremendous potential of deep-sea corals as sources for bioactive terpenoids. Finally, these findings raise intriguing questions about the evolutionary origins of these pathways within early cnidarians and across animal phyla more broadly.

## Significance

Octocorals are important foundational species in marine ecosystems and produce diverse terpenoids, many of which boast promising pharmaceutical activities. However, the ecological functions and evolutionary origins of these metabolites are poorly understood, especially in deep-sea corals, whose natural habitats are threatened by deep-sea mining operations. By characterizing deep-sea octocorals off the California coast, we found that they are comparable to shallow-water species in their broad terpenoid diversity and are a promising resource for biomedical innovation. We identified diterpenoid structure types that arose early in octocoral evolution and have persisted for hundreds of millions of years. These findings highlight the evolutionary importance and ecological complexity of terpenoid production in octocorals and pose questions about their functions in early metazoan development.

**Fig. 1.**
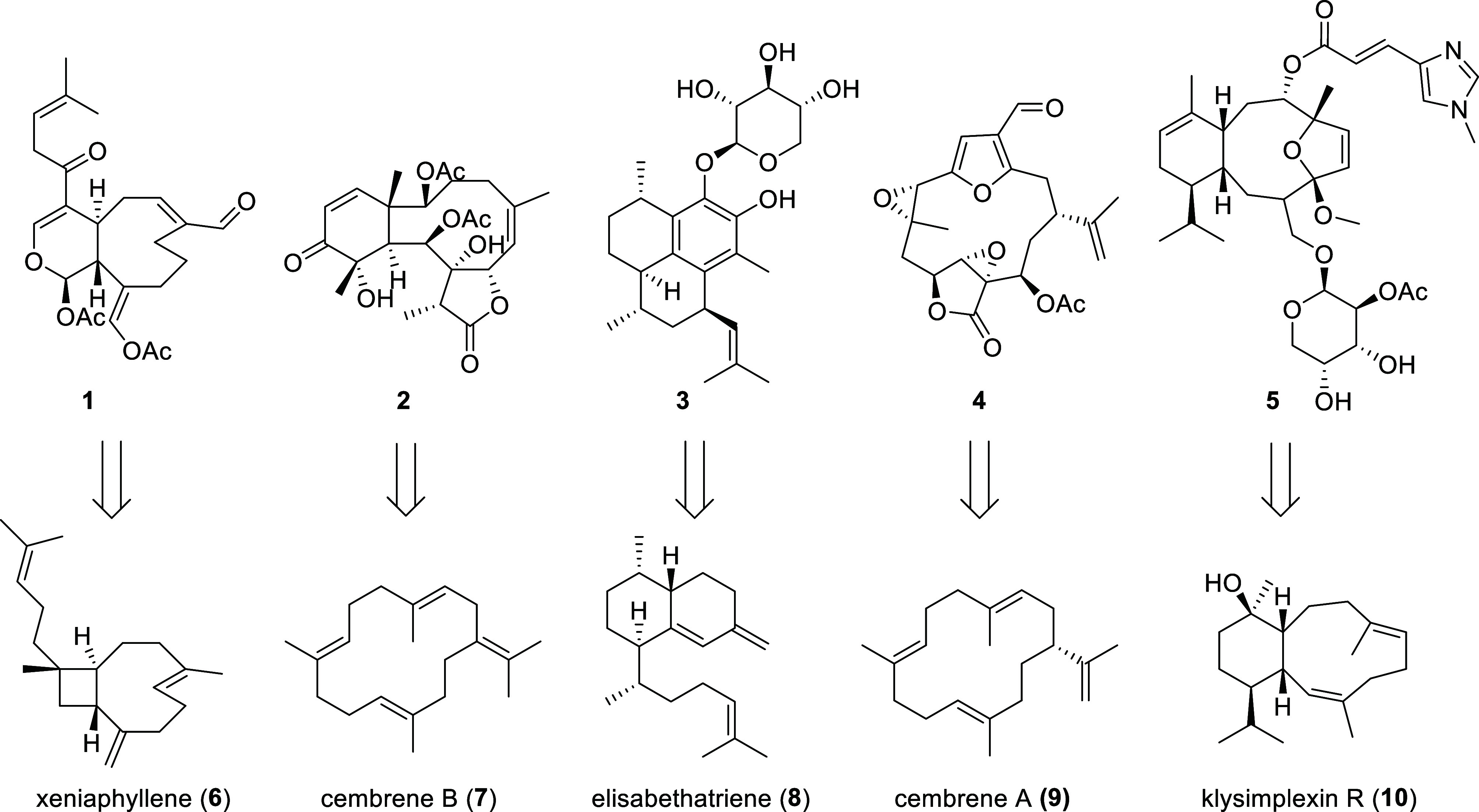
Examples of bioactive octocoral diterpenoids from the five largest structural classes and their biosynthetic precursors produced by octocoral terpene cyclases (TC).

Octocorals represent half of all coral species worldwide, totaling approximately 3,500 species (1). Since the split from their sister lineage ca. 770 million years ago, octocorals have colonized nearly all oceanic environments, from shallow tropical reefs to the abyssal zone below 4,000 m (2), with 75% of species living deeper than 50 m (3, 4). In contrast to hexacorals (stony corals, black corals, sea anemones), octocorals are known as a prolific source of specialized metabolites, constituting the largest known source of marine-derived terpenoids (5, 6). Over 4,000 octocoral-derived terpenoids have been described, many of which are of considerable medicinal and ecological interest due to their potent biological properties (7, 8). Prominent examples include cristaxenicin (**1**), with nanomolar antiplasmodial activity (9), renillafoulin A (**2**), which inhibits barnacle larval settlement (10), the anti-inflammatory pseudopterosin A (**3**) (11), the neuromuscular toxin lophotoxin (**4**) (12), and eleutherobin (**5**), known for pharmacological activity similar to the anticancer drug Taxol (Fig. 1) (13). A large number of the octocoral terpenoids identified to date are diterpenoids that are structurally related to compounds **1**-**5** (14). However, characterization of coral-derived terpenoids has disproportionately focused on shallow-water tropical species, with fewer than 100 compounds (~2.5%) known from cold- and deep-water species (15–17). This disparity contrasts markedly with the numerous deep-sea-specific species (3, 18). The primary reasons for this imbalance include difficulty accessing cold- and deep-water corals, which typically require specialized and costly equipment, such as remotely operated vehicles (ROVs). Moreover, the slow growth of deep-sea corals further restricts the collection of sufficient biomass for chemical studies (19, 20).

Recently, we (14) and Scesa et al. (21) demonstrated that octocoral genomes harbor genes coding for terpenoid biosynthetic pathways, dispelling earlier hypotheses of microbial symbiont origins. Octocoral genomes encode multiple enzyme classes essential for terpenoid biosynthesis, such as short-chain dehydrogenases, cytochrome P450s, and a phylogenetically distinct lineage of class I terpene cyclases (TCs). Class I TCs catalyze the first step in terpenoid biosynthesis, converting readily available oligoprenyl diphosphate precursors from central metabolism into specialized hydrocarbon structures (22). Coral TCs are monophyletic and broadly distributed across both octocoral orders, the Scleralcyonacea and Malacalcyonacea, which diverged from their last common ancestor around 540 million years ago (14, 23, 24). Notably, some of the initially characterized coral TCs produce diterpenes (C_20_-scaffolds) or monoalcohols closely resembling the principal carbon scaffolds of compounds **1**, **3**, **4**, and **5**: xeniaphyllene (**6**), elisabethatriene (**8**), cembrene A (**9**), and klysimplexin R (**10**), respectively (Fig. 1) (14, 21). These terpene hydrocarbons had previously been hypothesized (25–27) or experimentally linked (28) to higher oxidized terpenoids akin to compounds **1**, **2**, **3**, and **5** prior to genetic studies. We recently identified cembrene B (**7**) as the first biosynthetic intermediate for the briarane diterpenoids, including compound **2** (29). Cembrene B synthases are found within conserved biosynthetic gene clusters colocalized with genes encoding oxidative enzymes that construct the distinctive γ-lactone structural motif conserved in almost all briarane terpenoids. The cembrene B synthases were monophyletic and only detected within the order Scleralcyonacea. Beyond this recent finding, limited information exists on the evolutionary history and taxonomic distribution of coral TC genes, primarily due to sparse sequencing data at the time of initial studies (14, 21).

Leveraging evolving capabilities to connect genomic sequences with biosynthetic potential (30), we investigated the chemical and genetic diversity of terpenoid production in deep-sea octocorals. Our results show that deep-sea octocorals harbor a rich diversity of terpenoids comparable to shallow-water species. All but one investigated deep-sea species contained detectable terpenes within their tissues, and all possessed class I TC genes within their genomes. We performed a comprehensive phylogenetic analysis for octocoral TC genes, including 40 sequences obtained from nine deep-sea species described in this work and 137 sequences obtained from publicly available datasets spanning 39 species. Functional annotation of the enzymes revealed divergent evolutionary trajectories, suggesting the early emergence and conservation of diterpene biosynthesis across octocoral lineages.

## Results

### Sample Collection and Taxonomic Identification.

**Fig. 2.**
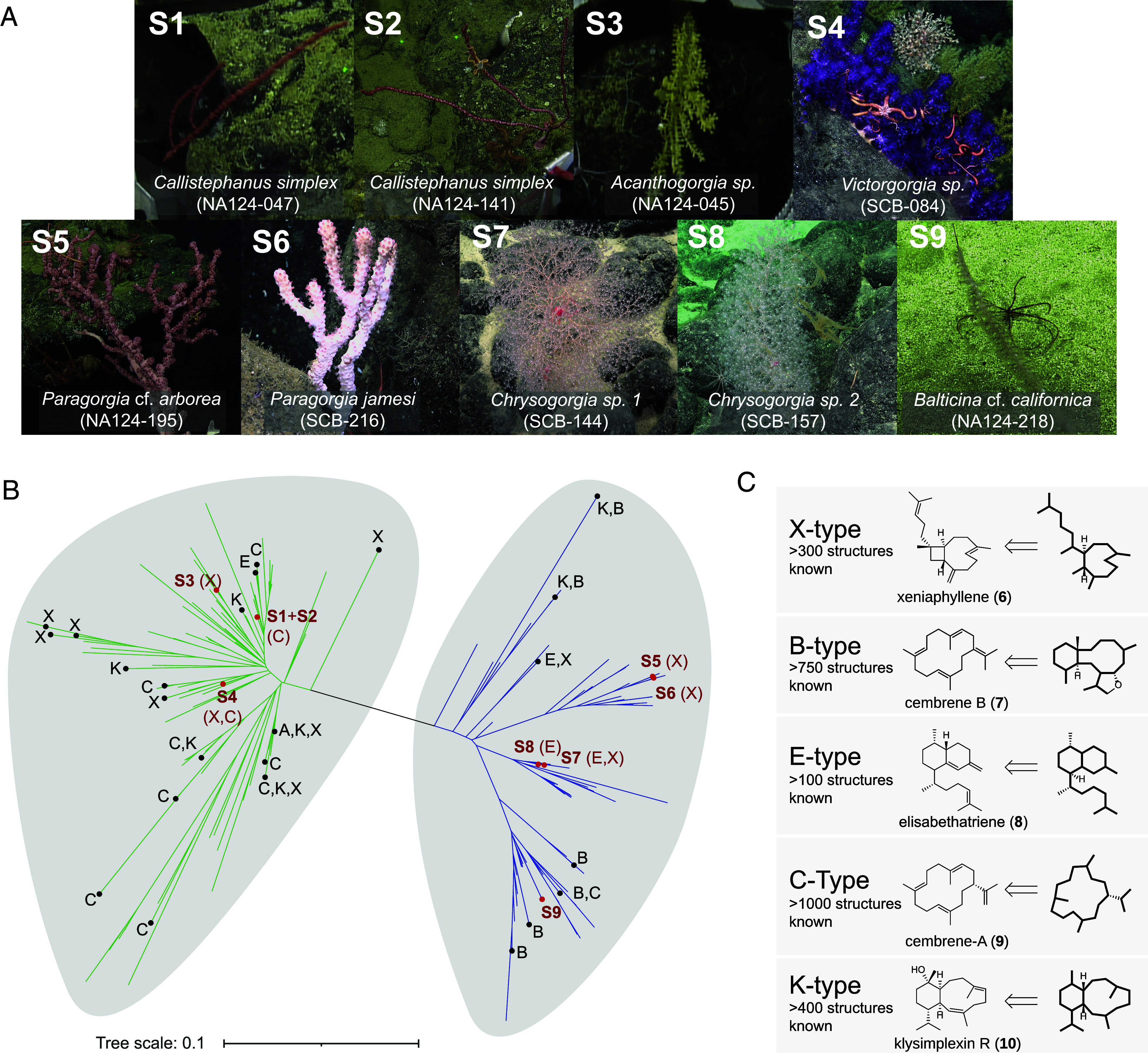
Taxonomic classification of deep-sea octocoral samples and distribution of diterpenoid compound types throughout octocorals. (*A*) In situ photographs of the deep-sea octocoral specimens analyzed in this study (S1–S9), labeled with their determined species identification. (*B*) Phylogenomic reconstruction of the nine octocoral mitogenomes sequenced in this work (highlighted in red labels corresponding to Panel *A*) alongside reference sequences from a published database (32). Branches are colored by taxonomic order: green for Malacalcyonacea, blue for Scleralcyonacea. Where applicable, detected diterpenoids are annotated by type (see Panel *C*). For a full representation of the Maximum Likelihood tree including bootstraps, see *SI Appendix*, Fig. S1; red font indicates diterpenes characterized from the deep-sea specimens in this study, black font denotes compounds previously reported in the literature. (*C*) Biosynthetic classification of octocoral diterpenoids based on their X-type (xeniaphyllene-derived), B-type (cembrene B-derived), E-type (elisabethatriene-derived), C-type (cembrene A-derived), and K-type (klysimplexin R-derived) structures, collectively referred to as XBECK-structure types.

Deep-sea octocoral samples were collected using remotely operated vehicles (ROV) from a series of offshore seamounts and ridges in the Southern California Borderland (depths range from 110 to 2,400 m; *SI Appendix*, Table S1) during two scientific cruises in 2020 and 2021. Nine specimens were initially characterized by morphological traits (Fig. 2*A*) and validated using phylogenomic analyses (Fig. 2*B*). Resulting taxonomic assignments spanned both orders Scleralcyonacea and Malacalcyonacea and included two *Chrysogorgia* spp., two *Paragorgia* spp., two *Callistephanus simplex*, a *Victorgorgia* sp., an *Acanthogorgia* sp., and a *Balticina* sp. (Fig. 2*A* and *SI Appendix*, Fig. S1) (31).

### Metabolomic Exploration of Samples and Distribution of Terpenoid Types Within Octocorals.

Metabolomic analysis of the nine deep-sea octocoral samples using gas chromatography-mass spectrometry (GC-MS) revealed substantial variability in sesquiterpene and diterpene hydrocarbon production. In one case only, *Balticina cf. californica* NA124-218, no terpenes were detected. In contrast, *Chrysogorgia sp*. SCB-144 produced a notably rich profile, with the identification of 22 sesquiterpenes and two diterpenes (*SI Appendix*, Figs. S2–S10). Overall, terpene production appeared to be driven more by fine-scale taxonomic relatedness than geographic location. For instance, the *C. simplex* specimens, despite being sampled from different collection sites (*SI Appendix*, Table S1), exhibited similar terpene profiles, consistent with species-specific chemical signatures previously observed in shallow-water corals such as *Sarcophyton* (33). However, these species-specific patterns were not conserved among closely related species (i.e., within genus). Minimal overlap in terpene profiles was observed between *Paragorgia jamesi* and *Paragorgia arborea*, as well as between the two distinct *Chrysogorgia* species.

Although overall terpene profiles were divergent across specimens, certain diterpenes exhibited broad taxonomic distribution. In particular, xeniaphyllene (**6**) was detected in distantly related corals from both orders, Malacalcyonacea (*Acanthogorgia* sp., *Victorgorgia* sp.) and Scleralcyonacea (*Paragorgia* sp., *Chrysogorgia* spp.) (Fig. 2*B*), suggesting that some diterpenes may be conserved or convergently evolved across octocoral lineages. The detected diterpenes were largely restricted to known coral compounds, xeniaphyllene (**6**), elisabethatriene (**8**), and cembrene A (**9**) (all confirmed by identical mass spectra to standards (14); *SI Appendix*, Figs. S2–S10). Only *Chrysogorgia* sp. SCB-157 produced previously uncharacterized diterpenes (*SI Appendix*, Fig. S9). In contrast, we observed far greater structural diversity among sesquiterpenes, including numerous uncharacterized compounds (*SI Appendix*, Figs. S2–S10). These patterns prompted us to examine whether terpenoid structural diversity more broadly reflects phylogenetic relationships within octocorals.

To investigate this, we expanded our analysis by incorporating published reports of diterpenoid production across octocorals, focusing on biosynthetic precursor relationships to compare diterpenoid types in a phylogenetic context. The structural diversity of octocoral diterpenoids is immense, and numerous principal carbon scaffolds have been described (8). However, many structurally distinct diterpenoids likely arise from common biosynthetic intermediates, with scaffold diversification occurring in late-stage tailoring steps, as suggested by the frequent coisolation of structurally related compounds or by chemical interconversion (26, 27, 34). Accordingly, we categorized diterpenoids into five types based on their presumed biosynthetic precursor rather than the final structure, which corresponded to major known coral diterpenoid scaffolds (Figs. 1 and 2*C*; see figure legend for details). Together, these five groups, referred to here as the XBECK-structure types, account for more than half of the over 4,000 reported octocoral terpenoids, including the examples in Fig. 1 (35). Four of the five diterpene types were broadly distributed across both octocoral orders, while the B-type was restricted to Scleralcyonacea (Fig. 2*B* and *SI Appendix*, Table S2), consistent with our recent finding that cembrene B synthases form a monophyletic clade within this order (29). The widespread taxonomic distribution of the other diterpene types suggested a distinct evolutionary history, prompting phylogenetic and functional analysis of the corresponding terpene synthases to investigate their biosynthetic origins.

### Terpene Synthase Phylogeny and Distribution.

**Fig. 3.**
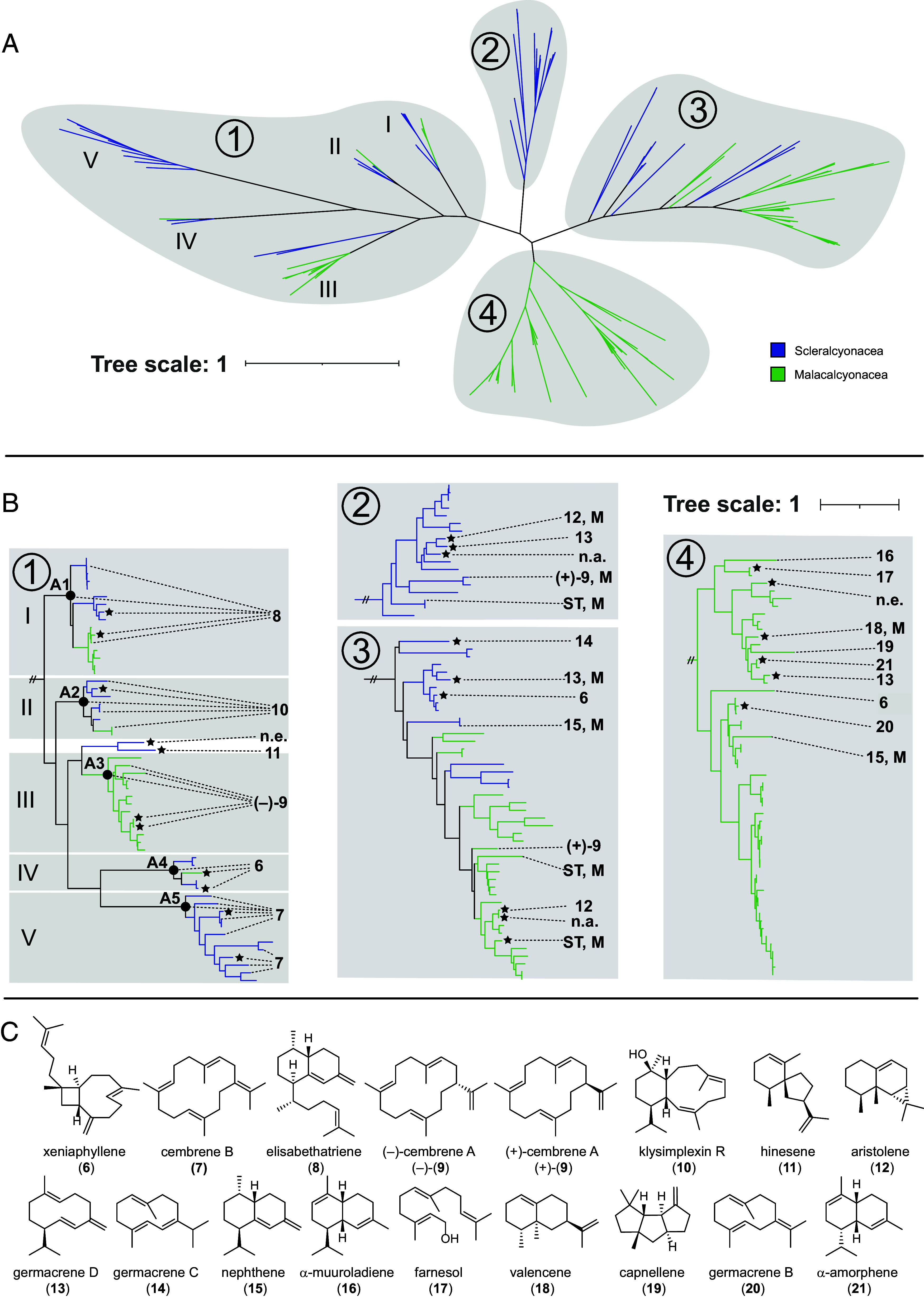
Phylogeny and enzymatic activity of octocoral TCs. (*A*) Unrooted phylogenetic tree showing the overall topology of octocoral TCs, with clades 1–4 and subclades I-V within clade 1. (*B*) Rectangular view of the phylogenetic tree. Labeled tips correspond to functionally characterized enzymes, annotated with their respective terpene products. Stars indicate enzymes from deep-sea octocorals newly characterized in this study. Filled circles mark ancestral nodes that have been biochemically characterized for each of the subclades 1-I through 1-V, labeled as A1 through A5, respectively. Abbreviations: M, multiple products; n.a., inactive protein; n.e., not expressed protein; ST, unknown sesquiterpene. (*C*) Structures of terpene products generated by characterized octocoral TCs.

All deep-sea coral genome assemblies generated in this work contained TC genes, their number ranging from one to seven per dataset. Phylogenetic analysis of these and publicly available octocoral TC genes revealed four major clades, with distinct taxonomic patterns across clades (Fig. 3*A* and *SI Appendix*, Fig. S11 and Supplementary File 1). Clades 1 and 3 contained TCs from both octocoral orders, whereas clade 2 was restricted to Scleralcyonacea and clade 4 to Malacalcyonacea. Clade 1 was further divided into five monophyletic subclades (I–V), with subclades 1-I, 1-II, and 1-IV containing representatives from both orders. In contrast, clades 2, 3, and 4 were constrained in their taxonomic distributions, with closely related sequences typically originating from the same family or genus (*SI Appendix,* Fig. S11). TC sequences derived from the deep-sea octocoral genomes generated in this study were distributed across all four clades as well as all five subclades in clade 1.

Functional annotation of previously characterized enzymes mapped onto the phylogenetic tree revealed apparent functional conservatism within clade 1 (Fig. 3*B*). This clade exclusively harbored diterpene synthases, including elisabethatriene (**8**) synthases in subclade 1-I, klysimplexin R (**10**) synthases in 1-II, cembrene A (**9**) synthases in 1-III, and cembrene B (**7**) synthases in 1-V (14, 21, 29, 36, 37). Subclade 1-IV contained no yet characterized enzymes. The remaining clades (2, 3, and 4) primarily contained sesquiterpene synthases, but also included a xeniaphyllene (**6**) synthase in clade 4, and two cembrene A (**9**) synthases in clades 2 and 3. However, no clear relationship between TC product type and phylogenetic placement was initially observed within these clades.

### In vitro Characterization of TCs From Deep-Sea Samples.

To investigate whether the phylogenetic structure of octocoral TCs reflects enzyme function, we characterized new TCs derived from the nine deep-sea specimens. Targeted genes were heterologously expressed in **Escherichia coli*,* and purified recombinant enzymes (*SI Appendix*, Fig. S12) were then incubated with the terpene precursors geranyl diphosphate (GPP), farnesyl diphosphate (FPP), and geranylgeranyl diphosphate (GGPP) to test for mono-, sesqui-, and diterpene production, respectively. We sampled two additional enzymes in each of the clades 1-I through 1-V to assess whether these enzymes generate diterpene products previously described from members of these clades. The only exception was clade 1-II, where we only identified one new sequence. Upon functional characterization, SCB-144-TC-3 and NA124-045-TC-3 from clade 1-I produced elisabethatriene (**8**); SCB-144-TC-7 from clade 1-II produced klysimplexin R (**10**); NA124-047-TC-3 and NA124-141-TC-4 from clade 1-III produced cembrene A (**9**); NA124-195-TC-5 and NA124-218-TC-1 from clade 1-V produced cembrene B (7) (Fig. 3*B* and *SI Appendix*, Fig. S13-34). Clade 1-IV contained no previously characterized enzymes, but both representatives characterized here (NA124-045-TC-5 and NA124-195-TC-3) produced xeniaphyllene (**6**). We also observed two paralogous sequences from the *Balticina* cf. *californicum* genome that were closest to subclade 1-III but separated by a fairly long branch and were hence not included in one of the subclades. We targeted both enzymes for characterization, NA124-218-TC-2 selectively producing hinesene (**11**) (*SI Appendix*, Fig. S34), a compound known from plants but not previously reported in corals. NA124-218-TC-3 failed to overexpress in *E. coli*. These results suggest that clade 1, excluding the two *Balticina* cf. *californica* TCs, consists of broadly distributed, functionally conserved diterpene synthases that likely contribute to the widespread occurrence of common diterpenoids across octocorals. Since the sampling density varied for each subclade, we generated sequences for each of the ancestral nodes from subclades 1-I to 1-V to see whether the ancestor of all identified extant sequences generates the same products as the individually tested enzyme representatives. Indeed, each ancestor reliably produced the same diterpene as the characterized extant enzymes from each clade, the products being elisabethatriene (**8**), klysimplexin R (**10**), cembrene A (**9**), xeniaphyllene (**6**), and cembrene B (**7**) for the ancestors of clades 1-I, 1-II, 1-III, 1-IV, and 1-V, respectively (*SI Appendix*, Figs. S35–S39).

Unlike clade 1, clades 2 to 4 showed greater variation in product diversity, with most previously characterized enzymes functioning as sesquiterpene synthases (Fig. 3 *B* and *C* and S11) and not showing obvious functional trends matching their phylogenetic relationship. We sampled across these three clades and tested 15 additional deep-sea enzymes. We aimed to cover a large phylogenetic space while also selecting some closely related enzymes for functional characterization. Adding the 15 new TCs to the 9 previously characterized enzymes (Fig. 3*B* and *SI Appendix*, Fig. S11) did not reveal significant functional conservation corresponding to phylogeny. Even enzymes with high sequence identity produced different products. For example, the **12** and **13** synthases in clade 2 (79% amino acid identity) and the **18**, **19**, **21**, and **13** synthases in clade 4 (57 to 79% amino acid identity), which exhibit similar sequence identities to functionally conserved diterpene synthases in clade 1 (*SI Appendix*, Tables S3–S7). Nine out of the total 24 characterized enzymes from clades 2 to 4 produced multiple major products, highlighting increased enzymatic promiscuity compared to clade 1. We also identified a xeniaphyllene (**6**) synthase in clade 3, indicating that **6** synthases occur in three of the four major TC clades. Together, these results fit the observed coral terpene profiles, with clade 1 enzymes showing conserved, predictable diterpene activity, while enzymes in clades 2, 3, and 4 displaying increased product promiscuity and functional divergence, mostly producing sesquiterpenes.

A few diterpene synthases were not located within clade 1. This observation included two previously characterized cembrene A (**9**) synthases from clades 2 and 3 and two xeniaphyllene (**6**) synthases, one characterized here, from clades 3 and 4. These enzymes clustered in distant, nonhomologous lineages relative to the corresponding clade 1 synthases. To test whether these enzymes produced truly identical products, we determined their absolute configurations. The products of cembrene A (**9**) synthases were compared to authentic standards of (–)-**9** (14) and (+)-**9** (38) by GC-MS on a homochiral stationary phase. This analysis showed that all (**9**) synthases from clade 1-III produced (–)-**9**, whereas the two enzymes from clades 2 and 3 produced (+)-**9**, proving their products to be distinct from enzymes in clade 1-III (Fig. 3 *B* and *C* and *SI Appendix*, Fig. S40). Conversely, optical-rotation measurements of preparative amounts of xeniaphyllene (**6**) obtained from enzymes in clade 1-IV and clade 3 identified them as (–)-**6**, and hence proved them identical to the product of the previously characterized **6** synthase from clade 4 (14). These results revealed that all **6** synthases identified so far form identical products, irrespective of their phylogeny or sequence identity.

Finally, we analyzed the chemical extraction data for the presence of compounds we identified in the in vitro characterization of enzymes. Whenever we characterized an elisabethatriene (**8**) synthase, a cembrene A (**9**) synthase, or a xeniaphyllene (**6**) synthase, we found the respective terpene product in the chemical extract of the specimen, with the exception of *Acanthogorgia sp*. NA124-045, where a **9** synthase was characterized, but the product was not observed in the extract (*SI Appendix*, Table S8). We did not observe klysimplexin R (**10**) or cembrene B (**7**) in any sample, even when we characterized an active diterpene cyclase from that specimen. Generally, the presence/absence correlation between detected diterpenes and a diterpene synthase in the predicted diterpene subclades 1-I through 1-V was strong and could be extended to species whose sequencing information was analyzed in this work and that had chemical data published in the literature (*SI Appendix*, Table S8). The two only exceptions to this were xeniaphyllene (**6**) detection in *Chrysogorgia sp*. SCB-144 and *Victorgorgia* sp. SCB-084, both genomes not encoding a sequence from subclade 1-IV. However, we characterized a phylogenetically distant **6** synthase with identical activity as the clade 1-IV enzymes in *Chrysogorgia sp*. SCB-144. The responsible synthase in *Victorgorgia sp.* remains unidentified. As many diterpene cyclases showed some degree of sesquiterpene synthase activity (*SI Appendix*, Figs. S13 and S34), we also analyzed the sesquiterpene content of the samples. Only in a few cases could we identify sesquiterpenes from in vitro assays in the chemical extracts of the same specimen, the examples being germacrene C (**14**) in *Paragorgia arborea* NA124-195 (*SI Appendix*, Fig. S5), germacrene D (**13**) in *Chrysogorgia sp*. SCB-144 (*SI Appendix*, Fig. S8), and aristolene (**12**) production in *Chrysogorgia sp*. SCB-157 (*SI Appendix*, Fig. S9). While **13** was detected as one of the products generated by elisabethatriene (**8**) synthases when incubated with FPP, we also identified a distinct **13** synthase in *Chrysogorgia sp.* SCB-144. Generally, the diterpene synthases produced multiple sesquiterpene products when incubated with FPP, while showing selective diterpene cyclase activity (*SI Appendix*, Figs. S13–S39). We did not observe a co-occurrence of the in vitro sesquiterpene blends with the diterpene products of enzymes in subclades 1-I to 1-V within the extracts of the deep-sea coral samples, pointing to selective diterpene production in vivo.

### Lineage-Specific Divergence and Selection in Terpene Cyclase Clades.

Next, we aimed to investigate whether the observed functional differences in clade 1 and clades 2, 3, and 4 occur due to distinct evolutionary mechanisms. We compared patterns of nucleotide divergence and synonymous site evolution in octocoral TC genes among the distinct clades and the subclades in clade 1 relative to mitochondrial genes. Although mitogenomes in octocorals show evidence of purifying selection and nuclear–mitogenome discordance (39, 40), they remain widely used as phylogenetic markers (e.g., mutS) and serve here as a conserved reference for comparative analyses. Functionally conserved diterpene synthase subclades within TC clade 1 partially exhibited constrained evolution, with subclades 1-II and 1-IV showing low divergence, while subclade 1-III and the more functionally diverse clades 2 and 4 diverged three- to four-fold more rapidly (*SI Appendix*, Fig. S41*A*). These trends were broadly consistent with population genetic metrics (*SI Appendix*, Table S9), including lower dN/dS ratios in some conserved subclades, but overall, no single metric showed a uniform pattern across clades. Together, these results indicated that evolutionary rates and constraints vary among TC clades but do not follow a simple relationship with enzyme function or phylogenetic grouping.

**Fig. 4.**
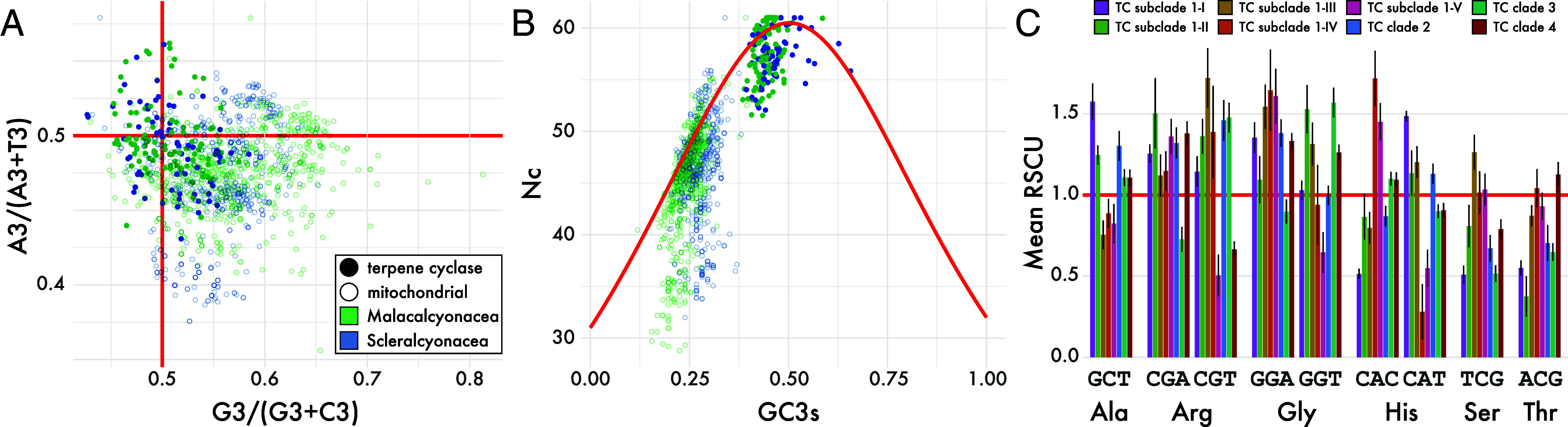
Codon usage patterns and synonymous codon biases in octocoral TC genes. (*A*) PR2 plot showing third-position nucleotide asymmetries in TC and mitochondrial genes. Each point represents an individual gene, colored by taxonomic order (Malacalcyonacea in green, Scleralcyonacea in blue). Red lines at 0.5 indicate expected equilibrium values under unbiased synonymous site evolution. TC genes exhibit greater variability in A/T and G/C asymmetries compared to mitochondrial genes, consistent with relaxed constraints or lineage-specific mutational biases. (*B*) Wright’s plot of effective codon number (Nc) versus third-position GC content (GC^3^) for TC and mitochondrial genes, colored by taxonomic order as in panel *A*. TC genes cluster at high Nc values, consistent with weak codon usage bias. The red curve shows the expected relationship between Nc and GC_3_ under neutral evolution. Mitochondrial genes are shown as a conserved comparative reference. (*C*) TC clade variation in relative synonymous codon usage (RSCU) for a subset of codons showing significant differences among TC clades (FDR < 0.05; RSCU range > 0.7). Codons are grouped by encoded amino acid, with bars colored by TC clade. These patterns highlight clade-specific synonymous site evolution in TC genes, suggesting lineage-specific mutational biases or weak selection on codon preference.

Taxonomic differences further contributed to variation in TC gene evolution. While mitochondrial genes showed no significant differences in nucleotide diversity (π) or allele frequency distributions (Tajima’s D) between the two octocoral orders (π: *P* = 0.8; Tajima’s D: *P* = 0.4), TC genes differed significantly in both metrics (π: *P* < 0.05; Tajima’s D: *P* = 0.06; *SI Appendix*, Fig. S41*B*), suggesting that terpene biosynthetic genes have been shaped, at least in part, by order-specific demographic histories or selection. Codon usage patterns were consistent with these broader trends. TC genes exhibited greater third-position nucleotide asymmetries than mitochondrial genes, with a significant order-specific bias in A vs. T usage (PR2_AT: *P* = 0.03) but no difference in G vs. C usage (PR2_GC: *P* = 0.22; Fig. 4*A*). Both gene sets showed a strong linear relationship between GC_3_ and overall GC content, but TC genes were systematically elevated in both, reflecting gene-specific mutational pressures (*SI Appendix*, Fig. S42*A*).

Codon bias in TC genes was modest overall, with most genes clustering at high effective codon number (Nc) values, consistent with weak codon usage bias (Fig. 4*B*). Nonetheless, TC genes exhibited broader codon repertoires and increased absolute codon frequencies for specific codons relative to mitochondrial genes (*SI Appendix*, Fig. S42*C*), highlighting compositional differences between gene groups. At finer scales, relative synonymous codon usage (RSCU) analyses identified a subset of codons with significant interclade differences (FDR < 0.05; RSCU range > 0.7) across TC genes, particularly for alanine, arginine, glycine, histidine, serine, and threonine (Fig. 4*C*). However, these patterns did not consistently align with enzyme function or evolutionary constraint, indicating that synonymous site evolution in TC genes is shaped by a combination of lineage-specific mutational biases, relaxed selection, and demographic history. The distinct codon usage patterns observed among clades are consistent with deep, independent evolutionary trajectories following the early diversification of multiple ancestral TC genes in octocorals.

## Discussion

The recent discovery of terpenoid biosynthetic pathways in octocorals prompted us to investigate their distribution and evolution across taxa and habitats. Here, we show that deep-sea octocorals, spanning six genera and both octocoral orders, produce species-specific sesquiterpenes and a restricted set of diterpenes. The latter corresponded to the precursors of XBECK-type diterpenoids, which encompass five structurally diverse groups originally reported from shallow-water species. Our findings highlight deep-sea corals as an untapped source of terpenoids, including likely novel XBECK-type diterpenoids, many of which exhibit promising bioactivities (Fig. 2*B*). Furthermore, the taxonomic distribution of these diterpenoid types reveals that those derived from **6**, **8, 9**, and **10** are found in both octocoral orders, consistent with an ancient evolutionary origin for their biosynthetic pathways.

This evolutionary pattern is mirrored in the phylogeny of octocoral TCs. Clade 1 comprises five monophyletic, isofunctional subclades of diterpene synthases that produce the precursors of the five major diterpenoid types, which was established by characterizing extant representatives as well as the reconstructed ancestors for all five subclades (Fig. 3*B* and *SI Appendix*, Fig. S11). These subclades show broad taxonomic representation, including sequences from both octocoral orders, consistent with ancient, conserved diterpene biosynthesis. In contrast, clades 2, 3, and 4 contain primarily sesquiterpene cyclases that exhibit rapid functional diversification and more limited taxonomic distribution (*SI Appendix*, Fig. S11), which corresponds to the species-specific sesquiterpene profiles observed via GCMS analysis. A similar evolutionary pattern was observed in plant TCs, where the ancestral function to produce *ent*-kaurene is preserved in all plants, while a vast array of additional TC copies producing a plethora of different products has evolved in different plant species (41). The long-term preservation of XBECK-type diterpene synthase activity, coupled with diversification of downstream tailoring enzymes, likely underpins the vast structural diversity of coral diterpenoids belonging to these five types. Indeed, functional diversification at early steps in these pathways (29, 42) would disrupt precursor production and associated biological functions, whereas diversification of late-stage redox enzymes can yield new derivative structures with novel or enhanced activity. While oxidized di- and sesquiterpenoids from octocorals have been linked to defensive roles (43, 44), the ecological roles of terpene hydrocarbons, specifically sesquiterpenes that might not represent pathway intermediates, remain unclear (45). Instead, they may serve functions like inter- or intraspecific communication, in which species-specific blends of compounds could be advantageous, analogous to volatiles in plants (46).

Comparative evolutionary analyses revealed distinct patterns across TC clades that partially align with their functional roles. The elisabethatriene (**8**)-, klysimplexin R (**10**)-, and xeniaphyllene (**6**)-producing diterpene synthase subclades (1-I, 1-II, 1-IV) exhibited slower divergence and elevated nucleotide diversity, consistent with functional constraint. In contrast, the cembrene A (**9**)- and cembrene B (**7**)-producing subclades (1-III, 1-V) showed divergence rates comparable to the more recently diversified, functionally heterogeneous sesquiterpene cyclases of clades 2-4. These patterns are further supported by synonymous site evolution and codon usage, which varied among TC clades in a manner consistent with their distinct evolutionary histories. A clear trend that differentiates the functionally conserved groups in clade 1 from clades 2, 3, and 4 is lacking. However, the clade-specific variation likely reflects a combination of lineage-specific mutational biases and relaxed selection, superimposed on deep, independent diversification of each TC lineage. In addition, significant differences in nucleotide diversity, synonymous site composition, and codon usage between octocoral orders across all TC clades indicate that demographic history and selection have further shaped TC gene evolution beyond phylogenetic divergence alone.

**Fig. 5.**
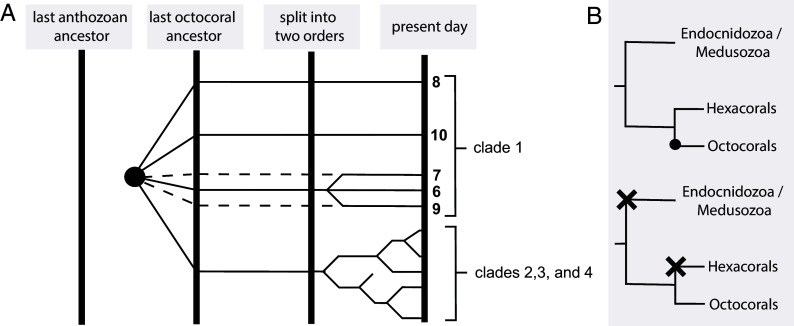
Schematic of possible early TC evolution in octocorals. (*A*) Diversification of different TC lineages throughout four timed events, marked by vertical bars. The latest plausible acquisition of the ancestor gene to all octocoral TCs is marked by a black circle. Diterpene lineages are marked with their generated products. The diversification of clades 2, 3, and 4 is shown schematically to illustrate the prolific duplication-neofunctionalizations observed in the phylogenetic data. (*B*) Two competing hypotheses for the emergence of octocoral TCs. *Top*: Acquisition of the ancestral TC by the last common octocoral ancestor. The acquisition event is marked by a black circle. *Bottom*: The last common cnidarian ancestor had the capability to produce terpenes, but it was lost in the emerging lineages of the endocnidozoans and medusozoans and later by the hexacorals, after their split from octocorals. Loss events are marked as X.

The presence of multiple, functionally distinct diterpene cyclase clades with broad taxonomic representation, including three subclades comprising sequences from both octocoral orders, suggests that the last common octocoral ancestor possessed at least four TC genes: three producing **6**, **8**, and **10** and a fourth ancestral gene that diversified into clades 2, 3, and 4 (Fig. 5*A*). Whether clades 1-III and 1-V and their observed **9**- and **7**-synthase activity originated in the last common ancestor and were subsequently lost in one order, or evolved independently postdivergence, remains unclear. We previously postulated that a TC gene was acquired in octocorals via horizontal gene transfer from a microbial organism (14), a hypothesis that was supported by the monophyly of octocoral TCs and the absence of terpene biosynthesis in hexacorals and all other cnidarians (Fig. 5 *B*, *Top*). However, the existence of multiple monophyletic, functionally distinct TC clades sharing moderate sequence identity raises the possibility that terpene biosynthetic capability originated earlier. One plausible scenario is that the last common anthozoan, or even cnidarian ancestor, possessed terpene biosynthesis, which was lost independently in medusozoans, hexacorals, and other lineages (Fig. 5 *B*, *Bottom*). Similar patterns of widespread ancestral biosynthetic capability followed by multiple independent losses have been observed for enzymes connected to sterol biosynthesis in metazoans (47). The deep evolutionary origins of animal terpene biosynthesis, potentially predating modern cnidarian diversification, remain an intriguing possibility. The recent discovery of phylogenetically distinct sponge terpene cyclases (48) further supports the hypothesis that terpene biosynthesis was widespread in early animals and that further biosynthetic diversity remains to be discovered across the animal kingdom.

Together, these findings represent a comprehensive evolutionary analysis of octocoral terpene biosynthesis, revealing both ancient, conserved pathways and ongoing gene diversification. Future research will expand on linked genomic and chemical datasets, which will further refine our understanding of coral evolution and facilitate the exploration and biotechnological potential of their abundant biosynthetic capacity.

## Materials and Methods

Method details and bioinformatic procedures are available in the *SI Appendix*.

### Collection, Genome Sequencing, and Taxonomic Classification.

Coral specimens were collected in the Southern California Borderland using ROV equipment in the research vessels Nautilus and Falkor in the years 2020 and 2021, respectively, and cryopreserved until utilization. Coral DNA was extracted using a bead beating and phenol/chloroform protocol and sequenced using Illumina NovaSeq S4 PE150 technology. The octocorals were identified by morphological characteristics and mtMutS sequences extracted from the assembled genome sequencing data.

### Chemical Analysis of Coral Samples.

Metabolites were extracted from thawed coral tissue using methanol/dichloromethane 2:1 and analyzed using gas chromatography coupled to mass spectrometry GC-MS.

### TC Identification and Characterization.

TC genes were identified from the genome assemblies using hmm searches. Genes selected for characterization were amplified by PCR from genomic DNA, cloned into the expression vector pET28a(+) and heterologously expressed in *E. coli* BL21 (DE3). Enzymes were then purified by nickel-affinity chromatography and incubated with geranyl diphosphate (GPP), farnesyl diphosphate (FPP), and geranylgeranyl diphosphate (GGPP) in vitro. Enzymatic products were extracted with hexane or pentane and analyzed by GC-MS or NMR spectroscopy.

## Supplementary Material

Appendix 01 (PDF)

Dataset S01 (CSV)

## Data Availability

The Illumina genome sequencing data are deposited at NCBI under the Bioproject number PRJNA1321964 (31) as biosamples SAMN51231267 (*Acanthogorgia sp*. NA124-045), SAMN51231268 (*Callistephanus simplex* NA124-047), SAMN51231289 (*Callistephanus simplex* NA124-141), SAMN51231291 (*Paragorgia cf. arborea* NA124-195), SAMN51231357 (*Balticina cf. californica* NA124-218), SAMN51231358 (*Victorgorgia sp*. SCB-084), SAMN51231360 (*Chrysogorgia sp*. SCB-144), and SAMN51231382 (*Chrysogorgia sp*. SCB-157). Characterized terpene cyclase sequences are available at GenBank under the accession numbers PX409220, PX409222, PX409224, PX409225, PX409226, PX409227, PX409229, PX409230, PX409232, PX409233, PX409234, PX409235, PX409236, PX409237, PX409238, PX409240, PX409241, PX409242, PX409243, PX409244, PX409245, PX409246, PX409247, and PX409249. Voucher specimens are available at the SIO Benthic Invertebrate Collection under the catalog numbers Co3550 (*Acanthogorgia sp*. NA124-045), Co3547 (*Callistephanus simplex* NA124-047), Co3555 (*Callistephanus simplex* NA124-141), Co3563 (*Paragorgia cf. arborea* NA124-195), Co3562 (*Balticina cf. californica* NA124-218), W1692 (*Victorgorgia sp*. SCB-084), Co3583 (*Chrysogorgia sp*. SCB-144), and Co3584 (*Chrysogorgia sp*. SCB-157). Sample metadata and all terpene cyclase sequences are available in the *SI Appendix* and Supplementary File 1.
