# Supplementary material for: Diversification of diterpene biosynthesis occurred early in octocoral evolution
Source: Proc Natl Acad Sci U S A. 2025 Nov 26;122(48):e2520279122. doi: 10.1073/pnas.2520279122 (PMC12685057; doi:10.1073/pnas.2520279122)
Supplement: Supplementary file 1 — Appendix 01 (PDF) [file pnas.2520279122.sapp.pdf]

Supplementary Information for:

Diversification of Diterpene Biosynthesis Occurred Early in Octocoral Evolution

Immo Burkhardt[1]*, Hannah K. Bone[1], Natalie E. Grayson[1], Helena A. Leucke[1], Johanna Gutleben[1], Paul R. Jensen[1], Andrea M. Quattrini[2], Alexander B. Chase[3], Bradley S. Moore[1,4]*

1: Scripps Institution of Oceanography, University of California San Diego; 9500 Gilman Dr., La Jolla, CA 92093, USA; 2: Department of Invertebrate Zoology, Smithsonian National Museum of Natural History, Washington, DC 20560, USA; 3: Roy M. Huffington Department of Earth Sciences, Southern Methodist University, Dallas, TX 75205, USA; 4: Skaggs School of Pharmacy and Pharmaceutical Sciences, University of California San Diego; 9500 Gilman Dr., La Jolla, CA 92093, USA.

Contents

Materials and Methods 3
General Methods 3
Sample collection 3
Taxonomic assignment of coral samples 3
Terpene extraction and analysis 4
DNA extraction and sequencing 4
Genome assembly and phylogenomics 4
Terpene Cyclase Phylogenetic Analyses 5
Evolutionary analyses: divergence, population genetics, and codon usage Divergence. 5
Molecular cloning 7
Heterologous expression for analytical scale 7
Heterologous expression for preparative scale 8
Analytical scale assays 8
Preparative scale assays and structure elucidation of TC products 8
Determination of the absolute configuration of cembrene A (9), produced by different TCs 12
Ancestral sequence reconstruction 13
Supplementary Tables 15
Supplementary Figures 23
NMR spectra 64
Supplementary References 76

Materials and Methods

General Methods

All chemicals and solvents were used as received from the commercial supplier (Sigma-Aldrich, Fisher or TCI). The substrates GPP, FPP, and GGPP were synthesized as reported previously (1, 2). Authentic terpene standard for **20** and **18** were purchased from Sigma Aldrich and Cayman chemicals, respectively, the residual standards were obtained from our in-house library. Thin Layer chromatography was performed with TLC plates purchased from Merck (silica gel 60 F254, glass plates). Column chromatography was carried out using a Flash EZ prep combiflash system (Teledyne ISCO). If not specified otherwise, GCMS analysis was carried out on an Agilent 7890A gas chromatograph with Agilent 5975C mass spectrometer, using a Rtx-5Sil 30 m × 0.25 mm, 0.25 µ column. Oven program was as follows: hold 70 °C for 3 min, 10 °C/min to 325 °C, hold at 325°C for 3min. Flow rate: 1 mL/min, Injection volume: 1 µL, splitless injection, Inlet temperature: 270 °C, MS transfer line temperature: 280 °C, MS source temperature 230 °C, MS quad temperature: 150 °C. NMR spectra were recorded on a JEOL ECZ spectrometer (500 MHz) in C_6D_6 and were referenced against C_6D_6 (δ = 7.16 ppm) for ^{1}H-NMR and C_6D_6 (δ = 128.06 ppm) for ^{13}C-NMR. Data for NMR spectra are reported as follows: shift (δ) in ppm, s = singlet, d = doublet, t = triplet, q = quartet, m = multiplet or unresolved, br = broad signal, J = coupling constant(s) in Hz. Optical rotation measurements were carried out using a Jasco P-2000 polarimeter.

Sample collection

Samples for this work were collected on two deep sea exploration expeditions, covering an area of approximately 39 000 km^2 of the "Southern California Borderland" (SCB), an area off the southwest coast of the US. The SCB extends from Point Conception to the border with Mexico and out to 300 km offshore. Sampling sites were selected with a focus on the presence of potentially commercially interesting mineral-rich hardgrounds, which are prevalent at slopes and ridges, as well as on off-shore seamounts. The first expedition on board the E/V Nautilus in November 2020 collected biological samples and oceanographic data at eight dive sites using the remotely operated underwater vehicle (ROV) Hercules, supported by the deep-tow system Argus. The second expedition on board Schmidt Ocean Institute's RV Falkor in August 2021 used ROV SuBastian to collect biological samples and oceanographic data at complementing eight dive sites Upon retrieval from the ROV, animal tissues were subsampled for DNA extraction (immediately frozen at -80 °C) and metabolomics (immediately frozen at -20 °C). For exact coordinates of samples investigated in this work see Table S1.

Taxonomic assignment of coral samples

Nine octocorals were identified using genomic data and morphological characters. To help identify octocorals, skeletal axis types and sclerite shapes were examined (3). mtMutS sequences, which are a mitochondrial informative DNA barcode(4), were then extracted from the assemblies. The data were aligned with muscle in AliView v 1.26 (5) against NCBI GenBank data.

Terpene extraction and analysis

Between 300 and 1000 mg of frozen coral tissue was separated from the samples, thawed and then macerated in 2 mL MeOH/DCM 2:1. The mixture was stirred for 12 hours in a closed 20 mL scintillation vial at room temperature. The resulting suspension was diluted with water (10 mL), 1 mL of hexane was added, and the sample mixed by vortexing for 10 seconds. After layer separation, the top layer was carefully pipetted off into GCMS vials and directly subjected to GCMS analysis. Chromatograms are shown in Figures S2-S10.

DNA extraction and sequencing

DNA was extracted using a bead beating and phenol:chloroform:isoamyl alcohol protocol as previously described (6). Subsampling and preparations were performed under sterile conditions inside a flow cabinet. Between 0.1 g and 0.5 g of tissue was thawed, cut into small pieces and used for DNA extraction. DNA was eluted into 20-80 µL of EB buffer. All DNA samples were additionally cleaned using a DNA clean & concentrator-100 kit (Zymo) and quantified using Nanodrop. DNA was submitted to the UCSD Institute for Genomic Medicine for library prep using the KAPA HyperPrep Kit (Roche) and metagenomic sequencing using 1 lane of Illumina NovaSeq S4 PE150 technology, targeting a read depth of 75M read pairs per sample.

Genome assembly and phylogenomics

We assembled high-quality draft genomes for nine deep-sea octocoral specimens using the BBMap toolkit v38.96 (7) for read trimming and quality filtering. Adapter sequences and low-quality regions were removed, and paired-end reads were merged prior to assembly with IDBA-UD v1.1.3 (8) using a k-mer size range of 30–200 with error correction enabled. Only contigs >2 kb in length were retained for further analyses. To exclude potential bacterial or archaeal symbiont sequences, we generated taxon-annotated GC-coverage (TAGC) plots, based on contig coverage (Bowtie2 v2.4.5) (9), GC content, and blastn v2.12.0+ searches against the NCBI non-redundant database (downloaded September 2022).

In addition to the deep-sea genomes, we included publicly available genomic and transcriptomic datasets from 34 representative octocoral species (Supplementary File 1). For datasets lacking pre-assembled genomes (i.e., *Capnella imbricata*; SRA accession SRR15733495), raw reads were retrieved from the SRA database and processed identically to the deep-sea samples.

All assemblies (N=43) were screened for the presence of 14 conserved mitochondrial marker genes (atp6, atp8, cob, cox1, cox2, cox3, mutS, nad1, nad2, nad3, nad4, nad4L, nad5, nad6) using diamond blastx (v2.1.6) (10) against a curated reference database of 184 octocoral mitochondrial genomes (11). To account for the incomplete nature of draft assemblies, we focused on a subset of seven mitochondrial markers (atp6, atp8, cox2, mutS, nad4, nad4L, nad6) that were consistently recovered across samples and exhibited more stable phylogenetic patterns in preliminary analyses. Samples lacking these seven markers or showing evidence of multiple divergent copies were excluded, resulting in a final dataset of 213 taxa for mitochondrial phylogenetic analyses.

Individual mitochondrial genes were aligned using Clustal Omega v1.2.4 (12) and concatenated into a multi-gene alignment. Phylogenetic relationships were reconstructed using RAxML-NG v1.2.0 (13) under a general time reversible (GTR)

model with gamma-distributed rate heterogeneity, starting from 20 randomized parsimony trees and assessed with 100 non-parametric bootstrap replicates.

Terpene Cyclase Phylogenetic Analyses
The nine deep-sea draft genome assemblies, along with the publicly available octocoral sequencing datasets, were queried for terpene cyclases using hidden Markov models (HMMs), as previously described (14). Briefly, open reading frames (ORFs) were translated using getorf (EMBOSS v6.6.0.0) and screened with hmmsearch (HMMER v3.3.2) (15) against custom TC HMM profiles. Putative hits were identified by manual checking for the conserved motifs DDXXD, NSE, and RY (14), and their corresponding nucleotide sequences were also extracted. In total, we identified 40 TC sequences from the deep-sea genomes and 137 TC sequences from public datasets, including previously characterized enzymes (Supplementary File 1). Both nucleotide and amino acid sequences were aligned separately. Nucleotide sequences were processed and phylogenies reconstructed as described above and used for all downstream evolutionary and population-level analyses. For visualization purposes, amino acid sequences of the TCs were aligned using Kalign v2.044, and a maximum-likelihood tree was generated in IQ-TREE v2.3.35 (16) using the Q.plant+I+R5 model selected by ModelFinder (17). Trees were visualized using iTOL v6 (18).

Evolutionary analyses: divergence, population genetics, and codon usage
Divergence.
To assess evolutionary congruence between terpene cyclase and mitochondrial gene divergence, we compared pairwise distances derived from TC phylogenies to corresponding mitochondrial phylogenetic distances for each genome where both data types were available. Analyses were performed separately for each major TC clade, with genome pairs excluded if mitochondrial data were unavailable or incomplete. For each clade-specific dataset, linear regressions were performed to assess the relationship between TC and mitochondrial divergence, and slope and adjusted R^2 values were reported.

Population Metrics. Population genetic statistics were calculated for TC genes and mitochondrial genes to assess evolutionary constraint and demographic patterns. Prior to alignment, TC nucleotide sequences were screened for complete open reading frames using a custom Python script to exclude sequences with frameshifts or premature stop codons. Genes passing this filter were aligned using Clustal Omega v1.2.4 and used for downstream analyses.

We estimated nucleotide diversity (π), Tajima's D, Watterson's theta (θ_W), and dN/dS ratios using the PopGenome (19), seqinr (20), and ape (21) packages in R. Z-scores and adjusted p-values for dN/dS were calculated based on variance estimates, and genes with unreliable estimates (e.g., negative or infinite dS) were excluded. Population genetic metrics were first calculated across all samples, then partitioned by octocoral order (Malacalcyonacea and Scleralcyonacea) based on mitochondrial phylogenetic assignment (see above). As a comparison, the same analyses were performed on the seven mitochondrial marker genes (atp6, atp8, cox2, mutS, nad4, nad4L, nad6) from their corresponding genomes. Because these analyses compare divergent octocoral lineages rather than populations within species, the application of dN/dS ratios is appropriate, consistent with prior expectations (22).

Codon Bias. Codon usage patterns were assessed for TC and mitochondrial genes to examine potential lineage-specific evolutionary pressures. Basic codon composition statistics, including third-position GC content (GC_3) and absolute codon frequencies, were calculated for each gene to examine correlations between overall GC content, codon preferences, and codon usage bias. Relative synonymous codon usage (RSCU) was also calculated for each gene using the seqinr package. RSCU values above 1.0 indicate preferential codon usage (with values >1.6 suggesting strong bias), while values below 1.0 indicate underrepresentation (with values <0.6 reflecting strong avoidance) (23). To test for differences in RSCU among TC clades, we performed statistical comparisons for each codon, applying false discovery rate (FDR) correction to account for multiple testing. Codons with FDR-adjusted p-values below 0.05 and an RSCU range exceeding 0.7 across clades were considered to exhibit biologically meaningful inter-clade variation.

To further investigate codon usage, calibrated CodonW v1.4.4 (24) analyses were performed following similar methodologies to investigate codon usage patterns in Demospongiae (25). As a calibration step, the mitochondrial gene atp8 was analyzed using the options -c_type 2 -f_type 7 -code 4, and sequences with internal stop codons or frameshifts were excluded based on log file filtering. All TC genes passing alignment quality filtering were subsequently analyzed with CodonW using the same options to generate consistent codon usage indices. Metrics extracted included the effective number of codons (Nc), where lower values indicate stronger codon bias (with Nc ≈ 20 representing maximal bias and Nc ≈ 61 indicating no bias), and the codon adaptation index (CAI), a measure of codon usage similarity to a reference set, with higher values reflecting greater adaptation to presumed translational preferences.

In addition to standard CodonW metrics, third-position nucleotide counts (A3s, T3s, G3s, C3s) were extracted from CodonW outputs. These were used to calculate PR2 bias metrics (PR2_AT and PR2_GC) in R, summarizing A/T and G/C asymmetries at third codon positions. Deviations from the expected PR2 equilibrium (PR2_AT = 0.5, PR2_GC = 0.5) can indicate strand-specific mutational biases or weak selection on codon composition (26).

Statistical Analyses. Comparisons of nucleotide diversity, Watterson’s theta, allele frequency distributions, and codon usage metrics between TC and mitochondrial genes and across taxonomic orders were performed using Wilcoxon rank-sum. All statistical analyses and data visualization were performed in the R software environment v4.2.2.

Molecular cloning

The protein encoding sequences of selected terpene cyclases were either cloned out of genomic DNA or ordered as original nucleotide sequences (Twist Biosciences). This was done using standard operations using commercially available kits. In brief, for genes that were cloned out of genomic DNA, (Supplementary File 1), primers specific to the gene of interest were designed (Table S11). PCR reactions were set up according to a standard protocol (Phusion High-Fidelity DNA Polymerase Master Mix, Thermo Scientific) based on the template DNA size and primer design. An example of the PCR conditions are as follows:

Repetitions	Temperature	Time
1x	98 °C	0:30 min
30x	98 °C	0:10 min
	56-70 °C	0:30 min
	72 °C	0:50 min
1x	72 °C	5:00 min

The resulting products were purified via a gel purification kit (Zymoclean Gel DNA Recovery Kit (ZymoResearch). Next, the purified terpene cyclase encoding genes, or the ordered genes were inserted into the *E. coli* expression vector pET28a(+) by cutting the empty vector with the restriction enzymes NdeI (NEB) and XhoI (NEB) in CutSmart® buffer (10x, NEB) at 37 °C for 4 h, and combining the open vector with the gene of interest using Gibson assembly (NEBuilder HiFi DNA Assembly Master Mix, New England Biolabs) according to the manufacturer's protocol. Subsequently, the plasmid is transformed into *E. coli* DH5α using chemically competent cells using the heat shock transformation method. Successful cloning and transformation of the template DNA was checked by sequencing the mini prepped plasmids (Quick-DNA Miniprep Kit, ZymoResearch). These TC-containing plasmids were transformed into *E. coli* BL21 (DE3) strains for heterologous expression of proteins.

Heterologous expression for analytical scale

Strains of *E. coli* BL21 (DE3) containing terpene cyclase encoding genes were grown from cryostocks in 10 mL of LB medium (Fisher) with 50 mg/mL kanamycin for 18 hours at 37 °C and 200 rpm in a shaking incubator. One mL of the starting culture was used to inoculate 100 mL of TB (Fisher) supplemented with 50 mg/mL kanamycin in a 250 mL Erlenmeyer flask. The culture was incubated at 37 °C and 200 RPM until the culture reached an OD of 0.6-0.8. Cultures were subsequently cooled to 18 °C, induced with 400 µM IPTG, and then shaken at 200 RPM for 18 hours. Cells were pelleted by centrifugation (8,000 g for 10 minutes at 4 °C) and resuspended in 5 mL of cold TC Lysis/Binding buffer (20 mM Na_2HPO_4, 0.5 M NaCl, 20 mM imidazole, 1 mM $MgCl_2$ at pH 7.4). Cells were lysed by sonication (2 seconds on, 2 seconds off for 1 minute, 30 seconds rest, repeated 2 times), and then the cell debris was pelleted using centrifugation (11,000g for 30 minutes at 4 °C). The supernatant was filtered with a 0.45 µM syringe filter and loaded onto a Ni-NTA column pre-equilibrated with TC binding buffer. The resin was washed twice with 2 mL of binding buffer each time, and the protein was eluted with 2.7 mL of TC elution buffer (20 mM Na_2HPO_4, 0.5 M NaCl, 0.5 M imidazole, 1 mM $MgCl_2$ at pH 7.4) into a 15 mL Falcon tube. Elution fractions were loaded onto a PD-10 column (Sephadex G-25 resin, Cytiva) pre-equilibrated with 25 mL of TC incubation buffer (50 mM Hepes, 5 mM $MgCl_2$, 250 mM NaCl, 10% glycerol at pH 7.8). The protein was then eluted with 3.5 mL of TC incubation buffer

into a 15 mL conical tube. The protein was used in enzyme assays directly or flash frozen in liquid nitrogen and stored at -80 °C for later use.

Heterologous expression for preparative scale

3 L of TB medium were inoculated with 1% vol of an *E. coli* BL21 (DE3) culture containing an expression plasmid with the respective terpene cyclase. Cultures were grown to an O.D. of 0.6-0.8 at 37°C with shaking. Once the O.D. was reached, the incubator was cooled to 18 °C, then induced with 400 µM IPTG and shaken at 200 RPM for 18 hours. Cells were pelleted using centrifugation (8,000 g for 15 min at 4 °C) and then resuspended in 60 mL of TC binding buffer (20 mM Na_2HPO_4, 0.5 M NaCl, 20 mM imidazole, 1 mM $MgCl_2$ at pH 7.4). The cells were lysed via sonication (15 seconds on, 45 seconds off, for a total of 7 minutes on-time). Cell debris was removed through centrifugation (11,000 g for 20 mins at 4 °C), and the supernatant was transferred to new tubes with the addition of DNase (Roche Diagnostics, 1 mg per 20 mL of supernatant) and centrifuged again (11,000 g for 20 mins at 4 °C). The supernatant was filtered through a 0.45 µm syringe filter and loaded onto a 5 mL His-Trap FF-NI column (GE). The column was washed with 40 mL of TC binding buffer (20 mM Na_2HPO_4, 0.5 M NaCl, 20 mM imidazole, 1 mM $MgCl_2$ at pH 7.4) and eluted with TS elution buffer (20 mM Na_2HPO_4, 0.5 M NaCl, 0.5 M imidazole, 1 mM $MgCl_2$ at pH 7.4), collecting in 2 mL fractions. Fractions were pooled and loaded onto a PD-10 column (Sephadex G-25 resin, Cytiva), pre-equilibrated with 25 mL TC incubation buffer (50 mM Hepes, 5 mM MgCl2, 250 mM NaCl, 10% glycerol at pH 7.8) in 2.5 mL batches. Each column was eluted with 3.5 mL of TC incubation buffer. The protein was then used in enzyme assays or flash-frozen in liquid nitrogen and stored at -80°C.

Analytical scale assays

Enzyme assays on an analytical scale were conducted in 1.5 mL of TC incubation buffer (50 mM Hepes, 5 mM $MgCl_2$, 250 mM NaCl, and 10% glycerol at pH 7.8) with 3 µM enzyme and 0.25 mg mL^{-1} of substrate (GPP, FPP, or GGPP) to test for mono, sesquiterpene, and diterpene synthase activity. Assays ran for 18 hours at room temperature. 200 µL of hexane were added to the cultures, the mixtures were vortexed, and then centrifuged in a microcentrifuge at maximum speed for 1 minute at room temperature. 150 µL of the extract was carefully pipetted off and analyzed by GC-MS. For assay results see Figures S13-S33.

Preparative scale assays and structure elucidation of TC products

Preparative scale assays were carried out to isolate products for Na124-TC-2 and SCB84-TC-6 and elucidate their structures. FPP (80 mg) was dissolved in ammonium bicarbonate solution (50 mM in 15 mL) and added dropwise to 600 mL of TC incubation buffer (50 mM Hepes, 5 mM $MgCl_2$, 250 mM NaCl, 10% glycerol at pH 7.8) with 3 µM enzyme concentration over 3 h. The assay was run for 18 h at room temperature, and then extracted with pentane (3 x 200 mL). The organic layer was dried over $MgSO_4$ and filtered. The resulting filtrate was then concentrated in vacuo to ~1 mL. The crude fraction was then purified using silica gel chromatography (100% pentane), with individual fractions checked with thin layer chromatography (silica plate, 100% hexane mobile phase) and stained with phosphomolybdic acid. Fractions containing the product were pooled, concentrated to dryness, and analyzed with NMR.

Analytical data for NA124-045-TC-5 product (xeniaphyllene **6**)

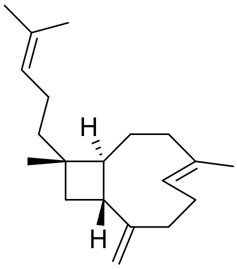

6

Optical rotation: $[\alpha]_D^{25.0}$ = −13.2 (c 0.17, $CHCl_3$). **^{1}H-NMR** (500 MHz, C_6D_6): δ = 5.38 (br. s, 1H), 5.28-5.19 (m, 1H), 5.04 (s, 1H), 4.87 (s, 1H), 2.44-2.29 (m, 2H), 2.17-2.10 (m, 1H), 2.09-1.90 (m, 5H), 1.85-1.77 (m, 1H), 1.77-1.70 (m, 1H), 1.70 (s, 3H), 1.60 (s, 3H), 1.58 (s, 3H), 1.51-1.44 (m, 2H) 1.42-1.33 (m, 2H) 0.98 (s, 3H) ppm. **^{13}C-NMR** (125 MHz, $CDCl_3$): δ = 154.7, 135.1, 130.8, 125.8, 124.9, 112.4, 52.6, 49.0, 44.6, 40.3, 39.7, 36.3, 35.0, 30.3, 29.0, 25.9, 23.4, 20.1, 17.7, 16.5 ppm.

NMR-data and optical rotation are in agreement with data for **6** produced by XsTC-1 (14).

Analytical data for SC144-TC-1 product (xeniaphyllene **6**)

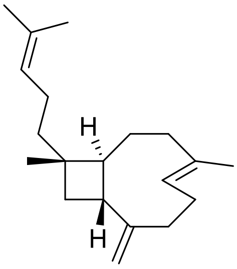

6

Optical rotation: $[\alpha]_D^{25.0}$ = −9.3 (c 0.03, $CHCl_3$). **^{1}H-NMR** (500 MHz, C_6D_6): δ = 5.38 (br. s, 1H), 5.28-5.19 (m, 1H), 5.04 (s, 1H), 4.87 (s, 1H), 2.44-2.29 (m, 2H), 2.17-2.10 (m, 1H), 2.09-1.90 (m, 5H,), 1.85-1.77 (m, 1H), 1.77-1.70 (m, 1H), 1.70 (s, 3H), 1.60 (s, 3H), 1.58 (s, 3H), 1.51-1.44 (m, 2H) 1.42-1.33 (m, 2H) 0.98 (s, 3H) ppm. **^{13}C-NMR** (125 MHz, $CDCl_3$): δ = 154.7, 135.1, 130.8, 125.8, 124.9, 112.4, 52.6, 49.0, 44.6, 40.3, 39.7, 36.3, 35.0, 30.3, 29.0, 25.9, 23.4, 20.1, 17.7, 16.5 ppm.

NMR-data and optical rotation are in agreement with data for **6** produced by XsTC-1 (14).

Analytical data for NA124-218-TC-2 product (hinesene, **11**):

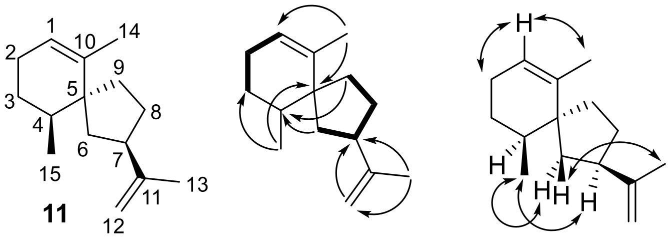

11

C^a	$^{13}C^b$	$^{1}H^b$
1	122.1	5.35-5.33 (m, 1H)
2	25.0	1.96-1.91 (m, 2H)
3	28.5	1.54-1.48 (m, 1H)<br>1.34-1.29 (m, 1H)
4	37.8	1.53-1.50 (m, 1H)
5	49.0	-
6	37.5	1.77-1.72 (m, 1H)
7	48.0	2.43 (qt, J=12.3, 6.5, 1H)
8	32.5	1.81-1.77 (m, 1H)<br>1.55-1.49 (m, 1H)
9	36.2	1.73-1.70 (m, 1H)<br>1.59-1.53 (m, 1H)
10	140.1	-
11	148.4	-
12	108.7	4.87 (s, 1H, H_Z)<br>4.81 (s, 1H, H_E)
13	21.4	1.70 (s, 3H)
14	20.1	1.67 (q, J = 1.9, 3H)
15	16.6	0.88 (d, J = 6.8, 3H)

[a]Carbon numberings as shown above table; [b]chemical shifts are given in ppm, coupling constants are given in Hertz, multiplicity: m = multiplet, s = singlet, d = doublet, t = triplet, q = quartet, br = broad.

NMR: See Table. **Optical rotation**: $[\alpha]_D^{28.0}$ = –11.8 (*c* 0.27, $CHCl_3$).

The absolute configuration was set as shown above by comparing the optical rotation to the literature value of enantioselectively synthesized (–)-**11** (27).

Analytical data for SCB84-TC-6 product (α-amorphene **21**):

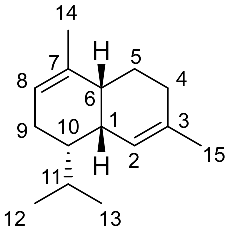

21

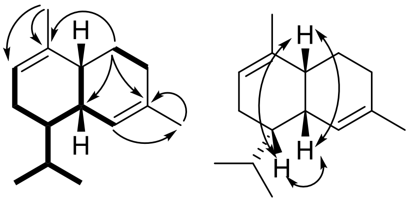

C[a]	^{13}C[b]	^{1}H[b]
1	c	2.69 (br s, 1H)
2	120.0	5.30 (s, 1H)
3	136.0	-
4	26.8	1.95-1.85 (m, 1H)<br>1.64-1.59 (m, 1H)
5	25.7	1.97-1.91 (m, 1H)<br>1.65-1.58 (m, 1H)
6	39.0	2.27 (br s, 1H)
7	133.4	-
8	124.5	5.51-5.47 (m, 1H)
9	27.6	2.04-1.96 (m, 1H)<br>1.73-1.64 (m, 1H)
10	45.4	1.19-1.13 (m, 1H)
11	29.1	1.61-1.54 (m, 1H)
12	21.0	0.95 (d, *J* = 6.6 Hz, 3H)
13	21.7	0.90 (d, *J* = 6.6 Hz, 3H)
14	20.9	1.65 (s, 3H)
15	24.3	1.64 (s, 3H)

[a]Carbon numberings as shown above; [b]chemical shifts are given in ppm, coupling constants are given in Hertz, multiplicity: m = multiplet, s = singlet, d = doublet, t = triplet, q = quartet, br = broad.

NMR: See Table. **Optical rotation**: $[\alpha]_D^{28.0}$ = −41.6 (*c* 0.045, CH_2Cl_2).

^{1}H-NMR data and optical rotation are in agreement with reported values from the literature (28).

Determination of the absolute configuration of cembrene A (9), produced by different TCs

For determination of the absolute configuration of cembrene A (**9**) from the enzymes DgTC-2, NA124-47-TC-3, NA124-141-TC-4, BaTC-1, and TmTC-1, the extracts of incubation experiments with GGPP and these enzymes were subjected to GC/MS analysis on the same gas chromatograph and mass detector as described in the general methods using a Cyclosil-B column (Agilent, 30 m length, 0.25 mm diameter, 0.25 µm film). A suitable GC Program for separating (+)- and (–)-**9** (increasing from 120 °C at 0.9 °C min^{-1} to 180°C and then increasing at 30 °C min^{-1} to 250 °C, operated in splitless mode) was determined by separating a racemic cembrene A (**9**) standard generated by mixing equal amounts of (+)- and (–)-**9**. Authentic standards for both enantiomers were obtained from in vitro assays using **9**-synthases that had been established as (+)-**9** (29) and (–)-**9** (14) synthases previously. The results are shown in Figure S40.

Ancestral sequence reconstruction

Amino acid sequences for ancestral nodes for the subclades 1-I to 1-V were determined using FireProt^{ASR} (30). We recreated a reduced phylogeny containing only the relevant sequences from clades 1 and 2 (as outgroup) using kalign2 and IQ-tree with the exact same parameters as described above. The topology of all subclades in clade 1 remained exactly the same with respect to the full phylogeny (Figure S43). The multiple sequence alignment and the phylogeny were used as input files for FireProt-ASR using the ancestral sequence reconstruction mode with default settings. The amino acid sequences from the ancestral nodes for subclades 1-I, 1-II, 1-III, 1-IV and 1-V were exported and ordered as synthetic genes codon-optimized for *E. coli* expression from Twist Biosciences. The genes were heterologously expressed, purified and characterized using the same methods as described above.

Determined ancestral sequences:

>Anc-clade-1-I

MSCSKEIHAPRKWVQRQKEMLKKKTDENLISMDELIELVVECGLCDEASVRKMYEKI
NTYQFMWCLVPTVPSDQWSREIFKTSLHFLCALFLVDDAVESYSEKEMKELSDAYD
MLEKQVCETFPNFPSINEMKQSLKHLKNPFDIASITFCMQYVNKIASILLKEGSTPQN
VVYNFRRRTSNAISISFQAVLIKSKCGSNVTSHEMLWRRVFDGLVILFYQFGELVSG
ATKNTQQHITVVTELRMLGCLYCIVINDLYSYHRDKLASSDNIIKTWLLEKTVSNLSEA
TSRCCQILDAVMQYMYERVEQCKQSHPGCPELEALLETTVYTTVGWIFAHTTVVPR
YSESPLKVALVEVEEEELPTWLAEKDEYGWNVVEKFLETMNDEKHKGILDALSGFV
DGRGQLLKTQL*

>Anc-cade-1-II

MSSTYEVTAPAKWVKHHKEVLSQPPDENLIAMDKLINWAVACGVTDEAGVRKSFKK
LNAYVYLRCMYPLVPSDPWSAEMFRINLHFTTAGYIVDDRIESYTMEEMNELCDGY
DMLEKEVSKTFPKCPSIEEMKESLKHLKNKFSIAAITMVMDFVNQSALFFLRQGKTS
RDRVDNFRRRLSNAVTIYYQAIRNKVKTGSNVTEGEMLWRRCFDVLAVPSYLAPES
FTHAIEKGEWPIRVLYELYMLGILYSTVINDLYSYHREKLDDCDNVIKVWLQEKSVSSI
EDANEKICQILDAILQFMYEKIEEAKAKYPNSPELQALLDYTGYVTAGWIFVHNTAAP
RYLESPYQVTLTEIEDNEIPNWLKEKDEFGWRVLRRFMETMNSEKGKAVMDALCGF
TDGRGILMT*

>Anc-cade-1-III

MSCDNNVRVPSKWTVPHKKMLAEEEDQELIALDELLRWVNETGLTTEEAAKIVFKKI
NAYFYLRCLYPILPDDPKSMKIFQLNLHFLILGYIIDDAIEKYNENEMKELISGYNLLQN
QVSETFPKFPSISEMKQLLGNVKNDFSKSAIVTIVDYVNKSTLILLEGGEIAEDKVVNF
RKRLSNAVAVYFDALLSKTKTGCEISEGEMLWRRCFDGLAIPVYMSTEVFSKTLEKN
HVLPVSEFYKLYLLGILFCVVINDLYSYERDKLDDTGDSIIKVWFKEKSVTDMTAATS
KISKILDAIIQQMYLFVEEGKARHPELSEWFERIAYMTVGWIYIHKTVVPRYVSSPFQI
TLVEIQENMIPNWLLEKDAYGQRVVQQFLENLNDPQQKNAMDFLYGSAIDLAD*

>Anc-cade-1-IV

MACSKHVRIPATWLYPHKEVLQDPPKEDLSTPKELVTWLCHEVKLADEDVVNKTFH
HLKPYFFVSLMYPRLPSDPWSMKLYELNVYFLVAAYILDDYHMETYPSTLVEEMVEA
YDSVNKEMDECFPDCPNGADIEKSLSSVENPSARNIIATMTDYINRSALIVWNSGRV
SLNRAADFRRRMAGTLRAYFHAVQMKNSEWTKVSVAQYLWNRCPESGAPCFYLS
NEFFSGFLETTSSIPMATFYYQYMYGSAVCSVINDLVSFYRDKNTDDVNIVKILDKSG
NAQDTEAAKSAILQLLESLIRYIYNSTKKMKKEYPECSSWFDFINDTTVGWIYIHTFTT
RYLSSPYKISIVPLEGEMLNDWLNEKENEFALRIIQEFQNVLEKEKDKMKMLYAF*

>Anc-cade-1-V

MSCTREVRVPVSWCVPLNQKSKSSTKLKKEIVDWCVKTGVASKSLAEKAVEQLNPF
LYMNILFPKVDPSDPSAAKLYEINASLIVGGYIVDDVLETYSLEALEELDNFFRSTEAW
VSKLLPDDYPTLDEISESLSEIKVPYSRAVICMFIDNFNKYCYEIWRNYKVSLSKVSNF
RRRLSASVTAYFEMAMAKRRNDAKIDEKEFLWQRSADNLGFPVLMLSEVLSGLLQK
ECENIPATITLHYFHMYSNLFAHVLNDLNSYHRDIHTDHNNLVKLWLQNGIATDFDDA
ATKIVQFLNSVIINIHKSVQKIKREFPNCPALQNFLESISYTFTGWVHVHTTAVERYKL
SPYQITLAKVEEGEVQQWLQDESEFGKRCVQMFDELMQEREEEMVLIYGLD*

Supplementary Tables

Table S1: Biological samples used in this study.

Sample number	Taxonomic ID	Depth [m]	Latitude	Longitude	Cruise	Terpenoids reported from genus
NA124-045	*Acanthogorgia* sp.	884.2	33.036	-121.000	Nautilus 2020	yes (31, 32)
NA124-047	*Callistephanus simplex*	692.1	33.033	-120.998	Nautilus 2020	no
NA124-141	*Callistephanus simplex*	562.7	32.738	-120.023	Nautilus 2020	no
NA124-195	*Paragorgia* cf. *arborea*	1121.8	32.679	-118.122	Nautilus 2020	yes (33, 34)
NA124-218	*Balticina* cf. *californica*	109.0	33.353	-119.045	Nautilus 2020	no
SCB-084	*Victorgorgia* sp.	1269.0	33.146	-120.941	Falkor 2021	no
SCB-144	*Chrysogorgia* sp.	2477.3	31.901	-120.036	Falkor 2021	no
SCB-157	*Chrysogorgia* sp.	2415.6	31.899	-120.039	Falkor 2021	no
SCB-216	*Paragorgia jamesi*	459.8	33.101	-117.881	Falkor 2021	yes (33, 34)

Table S2: Literature reports of XBECK-type diterpenoids in various octocorals.

Name	Diterpene type
Clavularia inflata	X-type (35)
Erythropodium caribaeorum	K-type, B-type (36, 37)
Briareum asbestinum	K-type, B-type (38, 39)
Heliopora coerulea	E-type, X-type (38, 39)
Junceella fragilis	B-type (40)
Dichotella gemmacea	B-type (40)
Veretillum cf cynomorium	B-type, C-type (41, 42)
Stylatula elongata	B-type (43)
Pennatula aculeata	B-type (44)
Sarcophyton trocheliophorum	C-type (45)
Sarcophyton glaucum	C-type (46)
Sinularia flexibilis	C-type, K-type (47, 48)
Sinularia lochmodes	C-type (49)
Sinularia maxima	C-type (50)
Sinularia brassica	E-type (51)
Eunephthya thyrsoidea	X-type (52)
Cladiella pachyclados	K-type (53)
Anthelia glauca	X-type (54)
Protodendron repens	X-type (55)
Ovabunda macrospiculata	X-type (56)
Eunicella cavolini	K-type (57)
Antillogorgia elisabethae	E-type (58)
Antillogorgia bipinnata	E-type, C-type (59)
Leptogorgia alba	C-Type (60)

Table S3: Amino acid percent identity matrix for TCs from clade 1-I

CsTC-1	100															
RspTC-1	74.45	100														
SCB157_TC-3	77.86	81.16	100													
SCB144_TC-3	76.4	82.52	86.89	100												
HcTC-3g-4	58.87	60.84	57.88	59.61	100											
HcTC-3g-3	59.8	61.03	58.58	60.29	95.1	100										
HcTC-3	60.29	61.76	59.31	61.03	96.57	98.05	100									
HcTC-3g-1	60.54	62.25	59.31	60.78	97.06	97.8	99.02	100								
HcTC-3g-2	60.05	61.52	58.58	60.29	96.08	96.59	98.54	98.54	100							
NA124045TC-3	59.02	61.99	59.32	60.83	65.69	66.59	67.32	67.32	66.59	100						
PbTC-1	59.27	63.68	59.32	61.56	66.67	67.8	68.54	68.54	67.8	94.7	100					
PcTC-3	59.27	63.44	59.32	61.56	67.16	68.29	69.02	69.02	68.29	93.98	97.59	100				
AeTC-1	58.78	61.74	59.08	60.83	64.71	65.85	66.59	66.34	65.61	82.89	85.3	84.82	100			
AbTC-1	58.54	62.23	58.84	60.58	64.46	65.37	66.1	66.1	65.61	84.58	87.71	87.47	89.26	100		
GvTC-1	59.27	62.47	59.08	61.07	64.22	65.61	66.1	65.85	65.37	86.75	88.92	89.16	90.14	92.79	100	
gGvTC-2	59.51	62.95	59.32	61.07	64.46	65.85	66.34	66.1	65.61	86.99	89.16	89.4	90.14	92.79	99.04	100

Table S4: Amino acid percent identity matrix for TCs from clade 1-II

EvTC-2	100						
VgTC-2	76.11	100					
BaTC-2	75.12	83	100				
BspTC-1	74.88	83	98.52	100			
ErycarTC-1	62.56	70.69	70.94	70.94	100		
SCB_144_TC7	67.24	75.86	73.4	73.15	73.4	100	
CdTC-3	61.82	67.98	67.49	66.75	64.53	77.4	100

Table S5: Amino acid percent identity matrix for TCs from clade 1-III

StTC-1	100												
PbTC-3	59.29	100											
NA124_045_TC-2	58.12	88.1	100										
EvTC-1	58.73	79.19	78.28	100									
NA124_141_TC-4	58.73	74.62	74.24	79.4	100								
NA124_047_TC-3	58.73	74.62	74.49	79.65	99.25	100							
gGvTC-3	55.7	70.38	68.26	71.11	82.41	82.66	100						
AbTC-2	53.67	68.27	68.69	72.36	82.91	83.17	85.18	100					
gGvTC-5	54.94	70.81	70.2	73.62	84.92	85.18	88.69	88.94	100				
DgTC-2	55.19	64.54	64.72	62.69	61.68	61.68	57.72	58.63	60.41	100			
ErTC-2	60.2	71.36	72.26	71.76	69.97	69.47	64.12	65.65	65.9	67.86	100		
TspTC-12	60.2	73.1	74.24	72.51	72.29	72.29	66.08	67.25	68.26	74.31	75.06	100	
SCB_084_TC-3	61.71	73.35	74.24	73.24	72.8	72.8	65.83	66.5	68.01	75.06	75.32	96.44	100

Table S6: Amino acid percent identity matrix for TCs from clade 1-IV

HcTC-5-2	100				
HcTC-5g-1	93.69	100			
NA124_045_TC5	61.36	61.36	100		
SCB_216_TC1	64.14	65.66	68.94	100	
NA124_195_TC3	63.64	64.9	68.43	97.98	100

Table S7: Amino acid percent identity matrix for TCs from clade 1-V

RmTC-1	100											
Rkoe_cbTC	82.49	100										
Basb_cbTC	49.24	47.45	100									
ErycarTC-6	51.65	49.87	59.6	100								
VgTC-1	48.86	48.35	52.14	50.76	100							
Selo_cbTC	52.79	52.3	55.42	55.7	75.06	100						
NA195_TC-5	50.38	48.59	56.09	61.83	53.81	55.73	100					
CrTC-1	50.9	49.1	57.61	62.6	53.81	55.98	93.91	100				
Crub_cbTC	50.9	49.1	57.61	62.6	53.81	55.98	93.91	100	100			
Dgem_cbTC	55.7	53.05	60.71	62.37	55.53	58.44	65.48	65.74	65.74	100		
PcaTC-1	54.43	51.52	52.9	56.31	60.05	61.71	55.33	55.84	55.84	58.25	100	
NA124_218_TC-1	56.96	53.94	57.18	59.85	62.81	65.24	59.9	60.66	60.66	65.41	67.92	100

Table S8: Comparison of identified diterpene type and terpene cyclase clades present in sequencing data if both are available from the same species.

Name	Diterpene type	Represented TC clades
Callistephanus simplex NA124-047[1]	C-type	1-III, 3, 4
Callistephanus simplex NA124-141[1]	C-type	1-III, 3, 4
Acanthogorgia sp. NA124-045[1]	X-type	1-I, 1-III, 1-IV, 3, 4
Victorgorgia sp. SCB-084[1]	C-type, X-type	1-III, 4
Paragorgia cf arborea NA124-195[1]	X-type	1-IV, 1-V, 3
Paragorgia jamesi SCB-216[1]	X-type	1-IV, 3
Chrysogorgia sp. 1 SCB-144[1]	E-type, X-type	1-I, 1-II, 2, 3
Chrysogorgia sp. 2 SCB-157[1]	E-type	1-I, 2, 3
Balticina cf. californica NA124-218[1]	-	1-V
Erythropodium caribaeorum[2]	K-type, B-type (36, 37)	1-II, 1-V, 2, 3
Briareum asbestinum[2]	K-type, B-type (38, 39)	1-II, 1-V, 2
Heliopora coerulea[2]	E-type, X-type (38, 39)	1-I, 1-IV, 2, 3
Dichotella gemmacea[2]	B-type (40)	1-V
Stylatula elongata[2]	B-type (43)	1-V
Sarcophyton trocheliophorum[2]	C-type (45)	1-III
Antillogorgia elisabethae[2]	E-type (58)	1-I, 3
Antillogorgia bipinnata[2]	E-type, C-type (59)	1-I, 1-III, 3

1: Data taken from this work; 2: Data taken from the literature – see cited reference and supplementary file 1 for the source of chemical data and sequencing data, respectively.

Table S9: Population genetic metrics of coral TCs and mitochondrial genes from investigated octocoral species.

		number_sites	trans/tranv	Watterson (θ^w)	nucleotide diversity (pi)	Tajima's D	dN/dS
TC clade	1I	1263	1.925	141.039091	167.7583333	0.82965799	0.227922406
	1II	1236	1.617647	181.632653	183.9047619	0.073648255	0.665153741
	1III	1320	1.676301	142.393088	121.4666667	-0.653433697	0.212881189
	1IV	1188	1.651899	201.12	219.4	0.694513241	0.163847161
	1V	1227	1.992063	124.839507	134.6060606	0.368956268	0.181396701
	2	1284	1.441558	109.316546	104.4771242	-0.188735319	0.572548683
	3	1359	1.216667	29.828672	26.31802721	-0.422610959	0.291384175
	4	1374	1.887755	61.852105	63.11784512	0.073388753	0.136862777
mito	atp6	720	3.619048	16.353529	8.354019494	-1.505534658	0 (dN=0)
	atp8	297	1.941176	8.429654	2.596306894	-2.046994629	undefined (dS=0)
	cox2	801	4.111111	31.021126	18.96262184	-1.222045977	0.08306654
	mutS	3675	Na	NA	NA	NA	1
	nad4	1524	Na	NA	NA	NA	1
	nad4L	312	2.714286	8.773851	3.707966599	-1.713828086	0.101407718
	nad6	780	4.142857	18.222613	9.500789889	-1.480291239	0.072105138

Table S10: primers used in this study

Gene Name	Primer Sequence	Annealing Temperature
NA 124_045 TC1	**FWD**: ccgcgcggcagccatATGTCGTGTAGCAACGAAGT<br>**REV**: cctggtgccgcgcggcagccatATGTCCTCATGTAATAACG	**72**
NA124_045 TC2	**FWD**: gcctggtgccgcgcggcagccatatgATGTCTTGCGATAACGATGT<br>**REV**: gccggatctcagtggtggtggtggtggtgctcgagCTAATCACCTAAAATGTATTCAATT	**57**
NA 124_045 TC3	**FWD**: tgccgcgcggcagccatatgATGTCATGCAGTAAAGAAATTC<br>**REV**: gatctcagtggtggtggtggtggtgctcgagTTAGCTGATGTCCAATTGTG	**58**
NA 124_045 TC4	**FWD**: tggtgccgcgcggcagccatATGGCGTGCAGTAAAGAATT<br>**REV**:gtcgacggagctcgaattcggatccTCATTCTAGCAAAAGATTTGCATGC	**72**
NA 124_045 TC5	**FWD**: tgccgcgcggcagccatatgATGGCTTGCAGTAAACATATTCG<br>**REV**:gatctcagtggtggtggtggtggtgctcgagTCAAAATGCGTAAAGCATTTTCATC	**62**
NA 124_47 TC2	**FWD**: cggcctggtgccgcgcggcagccatATGTCCTGTAGCAAGGTAGTTCGATATCCAAATG<br>**REV**: tcgacggagctcgaattcggatccCTATGTAAACTGTCCAGATGCTGGAATCACGCCGG	**72**
NA 124_47 TC3	**FWD**:gcctggtgccgcgcggcagccatatgATGTCCTCATGTAATAACGC<br>**REV**: cagccggatctcagtggtggtggtggtggtgctcgagTCAACTGCACAGCATATCAA	**59**
NA 124_47 TC4	**FWD**: tggtgccgcgcggcagccatATGGCGTGCTCCAAAGAATTG<br>**REV**: cggagctcgaattcggatccTCAGAGATTTCCATGCATATTGACATCGCCGC	**72**
NA 124_141 TC1	**FWD**: tggtgccgcgcggcagccatATGGCGTGCTCCAAAGAACT<br>**REV**: gcttgtcgacggagctcgaattcggatccTCAGGCTCTCAAAAGATTTGCAC	**72**
NA 124_141 TC4	**FWD**: cctggtgccgcgcggcagccatATGTCCTCATGTAATAACGCCGTACGTGTCCC<br>**REV**: aagcttgtcgacggagctcgaattcggatccTCAACTGCACAGCATATCAACGACGTTGG	**72**
NA 124_141 TC5	**FWD**: tggtgccgcgcggcagccatATGGCGTGCTCCAAAGAATT<br>**REV**: cgcaagcttgtcgacggagctcgaattcggatccTCAGAGATTTCCATGCATATTGAC	**72**
NA 124_195 TC1	**FWD**: tgccgcgcggcagccatATGTCGTGCAGCAAAGAAAT<br>**REV**: cggagctcgaattcggatccTTATACTGGAATGAAATGTCCACGTGG	**72**
NA 124_195 TC3	**FWD**: tgccgcgcggcagccatatgATGGCTTGCAGTAAACACGTTCG<br>**REV**: gatctcagtggtggtggtggtggtgctcgagTCAAAATGCATAAAGCCTTTTCATC	**62**
NA 124_195 TC5 Exon 1	**FWD**:gcctggtgccgcgcggcagccatatgATGTCGATGGACAGGAAAATCTACGTTCC<br><br>**REV**: GTAACGGAATTCAGGAATTGTACGACCTTTGTTGCTGCATCGTCGAAATCAACTG	**69**
NA 124_195 TC5 Exon 2	**FWD**: GTTGATTTCGACGATGCAGCAACAAAGGTCGTACAATTCCTGAATTCC<br>**REV:** ggatctcagtggtggtggtggtggtgctcgagTTAATCTAAGCCATAAAGAAAAACCATC	**65**
NA 124_218 TC1	**FWD**:tgccgcgcggcagccatatgATGTCAAGCCTGCACAC<br>**REV**:gatctcagtggtggtggtggtggtgctcgagTTATTTACTCAATCCATAAAGAGCAATC	**60**
NA 124_218 TC2	**FWD**:tgccgcgcggcagccatatgATGTGGCGCACCGATGTT<br>**REV**:gatctcagtggtggtggtggtggtgctcgagTCATGGGATACTGAATTGTCTATCTAC	**63**

SCB 84 TC1	**FWD**: tgccgcgcggcagccatATGTCGTGTAGCAAGGAAGTTC<br>**REV**: cggagctcgaattcggatccCTATTTACTAAGTAAACTAAGTAAACTGTCCACAGACCGA	**72**
SCB 84 TC2	**FWD**: gcgcggcagccatATGTCGTGTAGCAAGGCAGT<br>**REV**: cggagctcgaattcggatccCTATTCATTAAGTAAACTAAGTACACTGTCCACAGAC	**72**
SCB 84 TC3	**FWD**: gcctggtgccgcgcggcagccatatgATGCGAGTGTACAATTCATG<br>**REV**: cagccggatctcagtggtggtggtggtggtgctcgagCTAAATTATACTGCAACCGTAC	**58**
SCB 84 TC5	**FWD**:tggtgccgcgcggcagccatATGCATAAGCACAACTTGAT<br>**REV**: cttgtcgacggagctcgaattcggatccTTAATTAAGTAAATTGTCAACTGCACG	**72**
SCB216 TC1	**FWD**: tgccgcgcggcagccatatgATGGCTTGCAGTAAACACGTTCG<br>**REV**: gatctcagtggtggtggtggtggtgctcgagTCAAAATGCATAAAGCCTTTTCATC	**62**
SCB 144 TC1	**FWD**: tggtgccgcgcggcagccatATGGCGTGCAGTAAACCGGA<br>**REV**: gcaagcttgtcgacggagctcgaattcggatccTCATGTTGGTACAAAATGTCCACACG	**72**
SCB 144 TC3	**FWD**: tgccgcgcggcagccatatgATGTCGTGCAGCAAGCC<br>**REV**: gatctcagtggtggtggtggtggtgctcgagTTATTTATCCAGTGTTTTTATTAACTGTCC	**62**
SCB 144 TC4	**FWD**: ccgcgcggcagccatATGGCGTACAGTAAACCAAT<br>**REV**: cggagctcgaattcggatccTCATACAACGTTATATAATCCATCCTC	**72**
SCB 144 TC7 Exon 1	**FWD**: tggtgccgcgcggcagccatATGCTGTCCACGTATGTTGT<br>**REV**: AACAGCGCCTCCAAAGCATGTCACCTTCAGTCACATTGGC	**72**
SCB 144 TC7 Exon 2	**FWD**: GCCAATGTGACTGAAGGTGACATGCTTTGGAGGCGCTGTTT<br>**REV**: cggagctcgaattcggatccCTAATTTTTAAACAGACCTTCTCCATCAGT	**72**
SCB 157 TC2	**FWD**: cgcgcggcagccatATGGCGTACAGTAAGCCAGA<br>**REV**: cggagctcgaattcggatccTCATGTTGGCACAAAATGTC	**72**
SCB 157 TC3	**FWD**: tgccgcgcggcagccatatgATGTCGTGCAGTAAACCG<br>**REV**: gatctcagtggtggtggtggtggtgctcgagTTATTCATCTAACACAAGTGTTTTTATTAA	**61**
SCB 157 TC5	**FWD**: ggcagccatATGGCGTATAGTAAACCAATACATGCTCCAAGTGTATGGGTAAA<br>**REV:** gtggtggtgctcgagTCATACAACGTTATACAATCCATCTTCTTTGTATGCAGATATTTT	**72**
SCB 157 TC6	**FWD**: gcctggtgccgcgcggcagccatatgATGGCATACAGTAAACCAATACAT<br>**REV**: ggatctcagtggtggtggtggtggtgctcgagTTATATGTCATACAATCCATCTTTTTGG	**61**
SCB 157 TC8	**FWD**: gcctggtgccgcgcggcagcgcctggATGGCATACAGTATCCCAG<br>**REV**: gccggatctcagtggtggtggtggtggtgctcgagTTATATGTCATAAAATCCATCATCC	**59**

Supplementary Figures

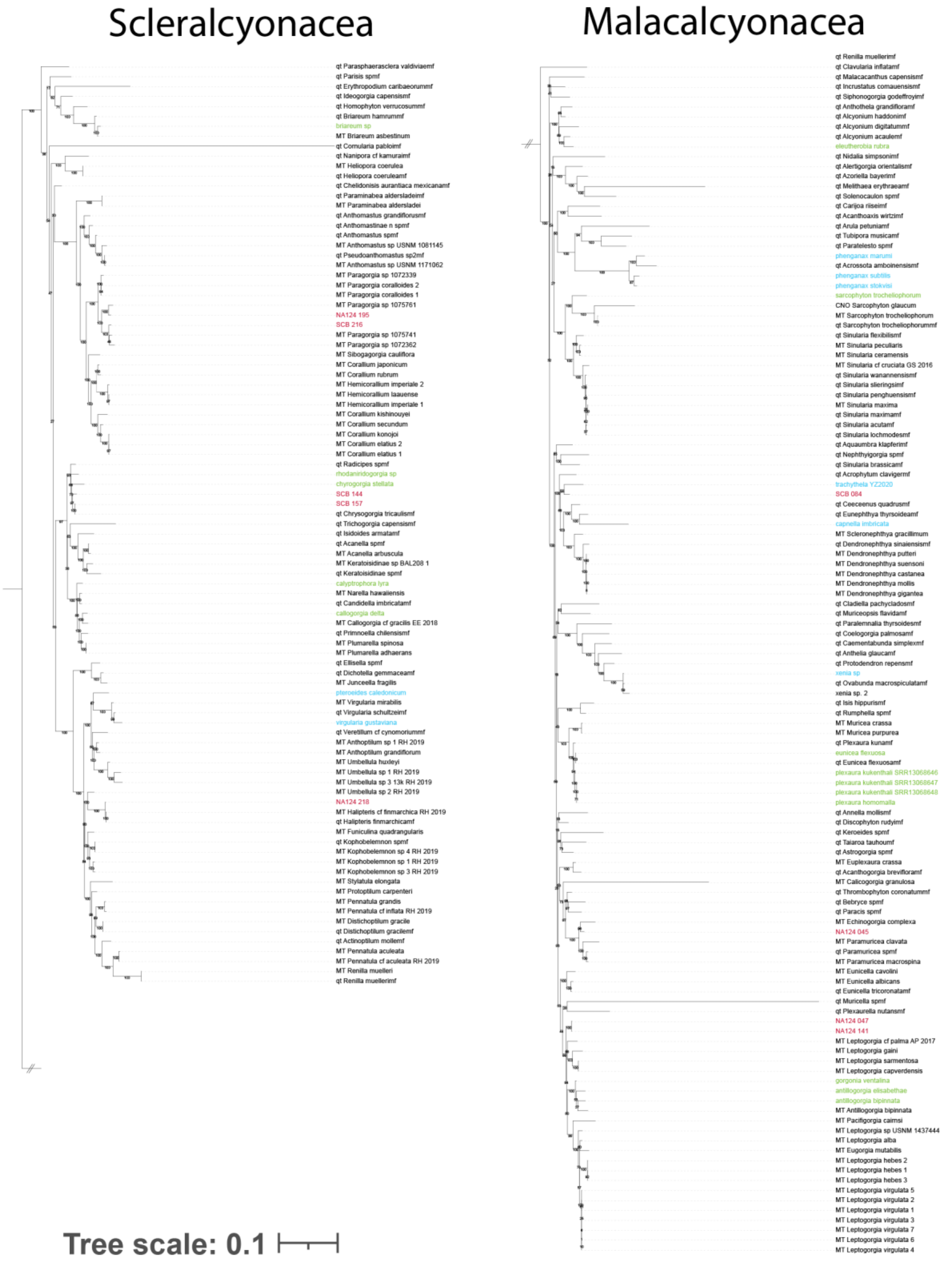

Figure S1: Phylogenomic tree constructed with octocoral mitochondrial genes. Black entries are taken from a published mitogenome database for octocorals (61), green entries are sourced from publicly available genomic data (supplementary file 1), blue entries are sourced from publicly available transcriptomic data (supplementary file 1), red entries are sourced from samples analyzed in this work (Table S1).

Acanthogorgia sp. - NA124-045

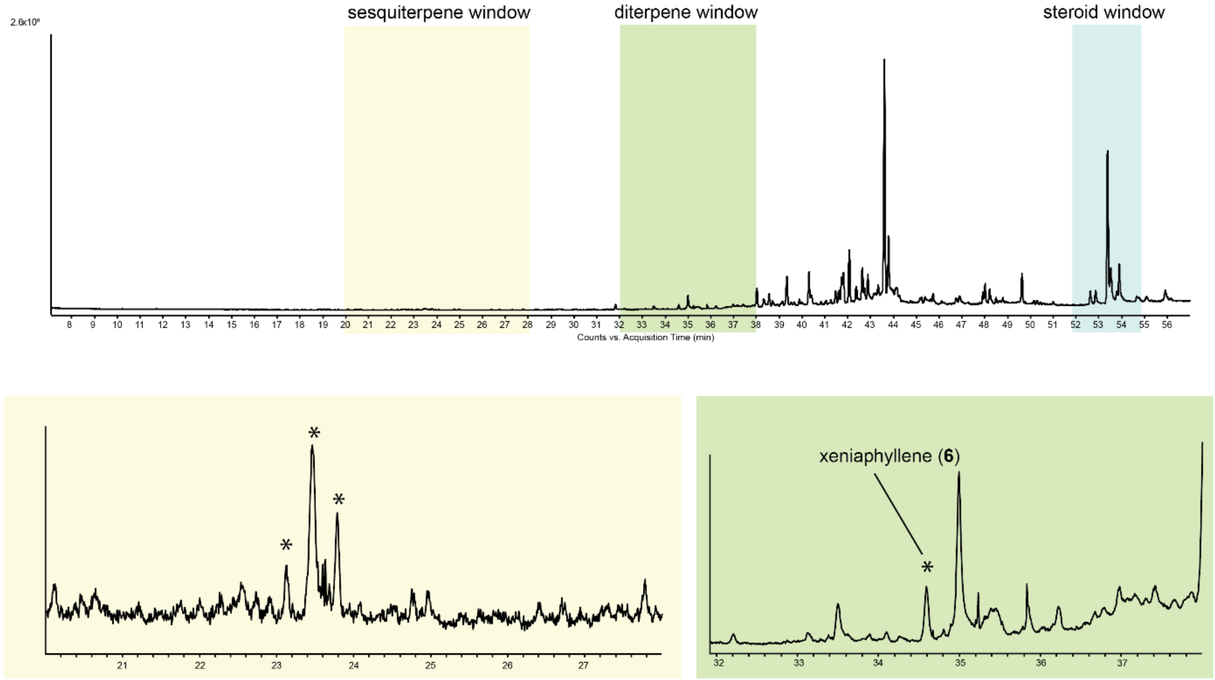

Figure S2: GCMS analysis of hexane extract derived from *Acanthogorgia sp*. NA124-045. Top: Complete TIC. Bottom: Magnified time windows for sesqui- and diterpene detection. Signals labeled with asterisks represent terpene hydrocarbons.

Callistephanus simplex - NA124-047

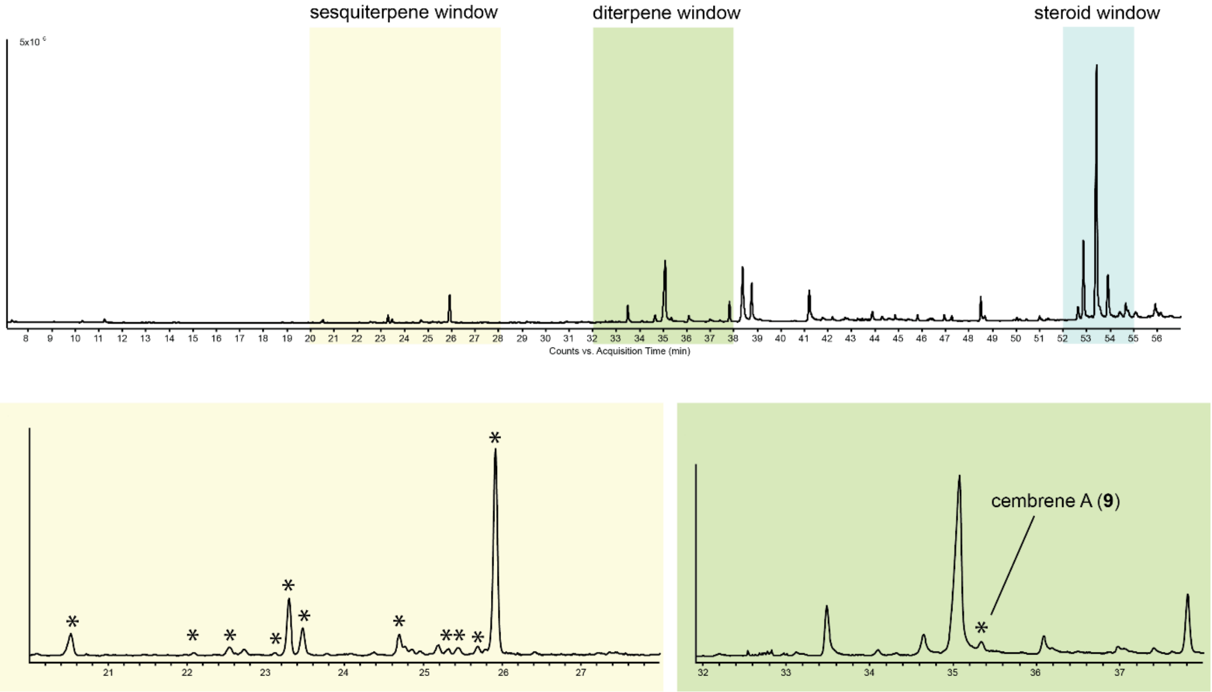

Figure S3: GCMS analysis of hexane extract derived from *Callistephanus simplex* NA124-047. Top: Complete TIC. Bottom: Magnified time windows for sesqui- and diterpene detection. Signals labeled with asterisks represent terpene hydrocarbons.

Callistephanus simplex - NA124-141

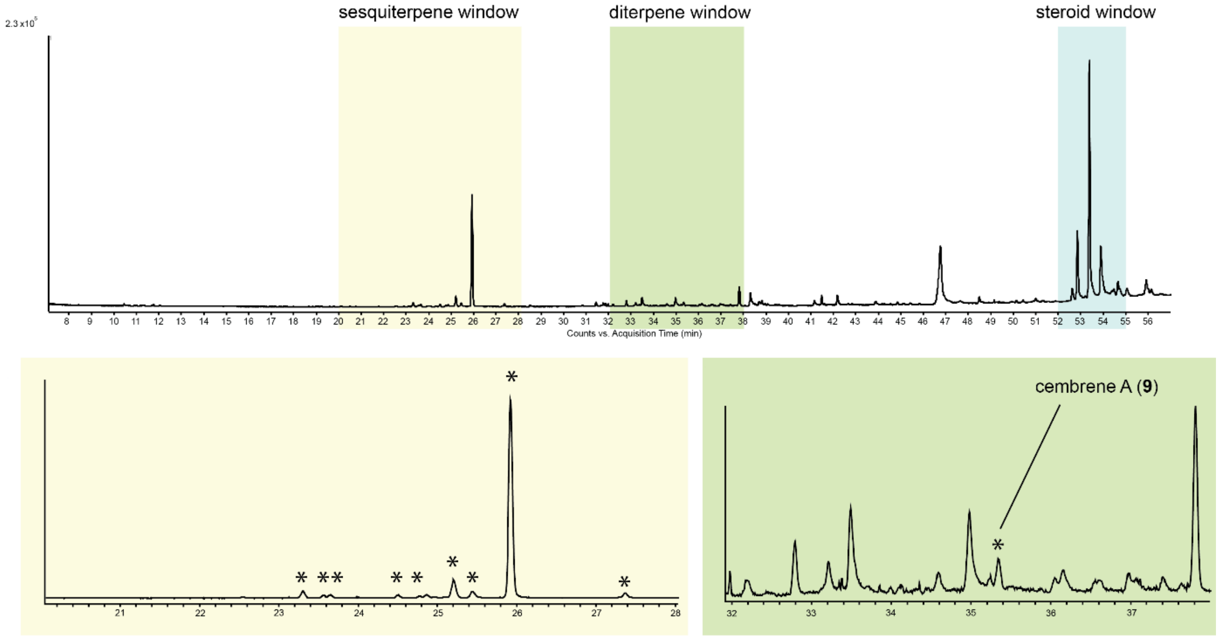

Figure S4: GCMS analysis of hexane extract derived from *Callistephanus simplex* NA124-141. Top: Complete TIC. Bottom: Magnified time windows for sesqui- and diterpene detection. Signals labeled with asterisks represent terpene hydrocarbons.

Paragorgia cf. *arborea* - NA124-195

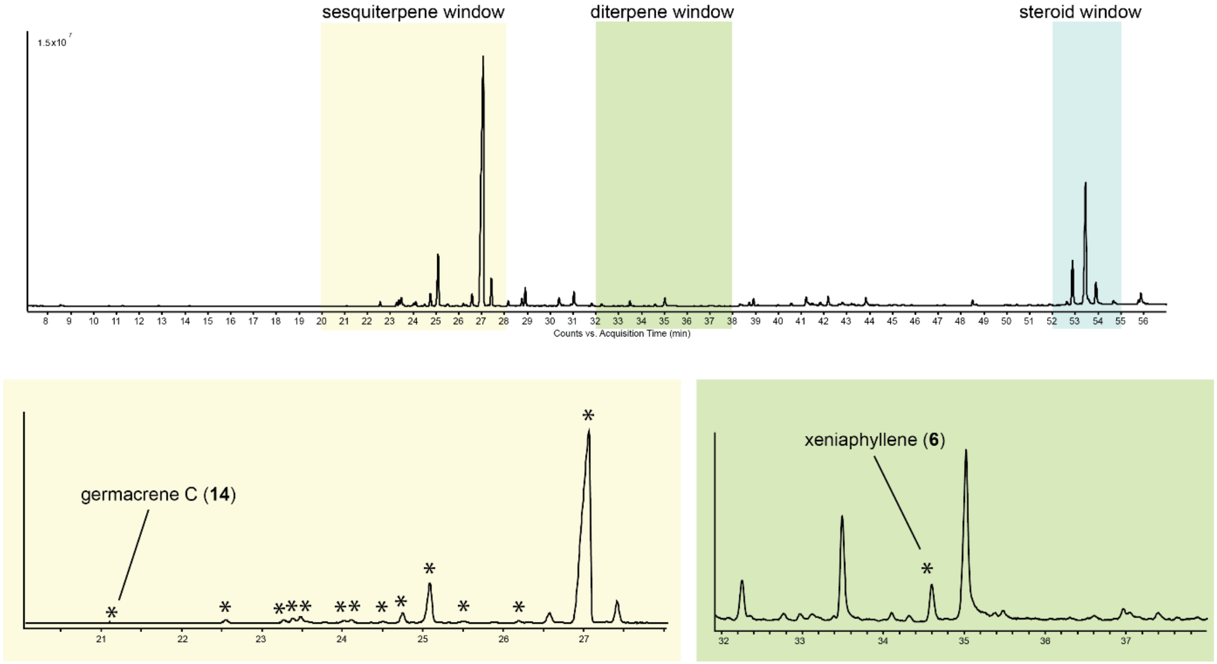

Figure S5: GCMS analysis of hexane extract derived from *Paragorgia* cf. *arborea* NA124-195. Top: Complete TIC. Bottom: Magnified time windows for sesqui- and diterpene detection. Signals labeled with asterisks represent terpene hydrocarbons.

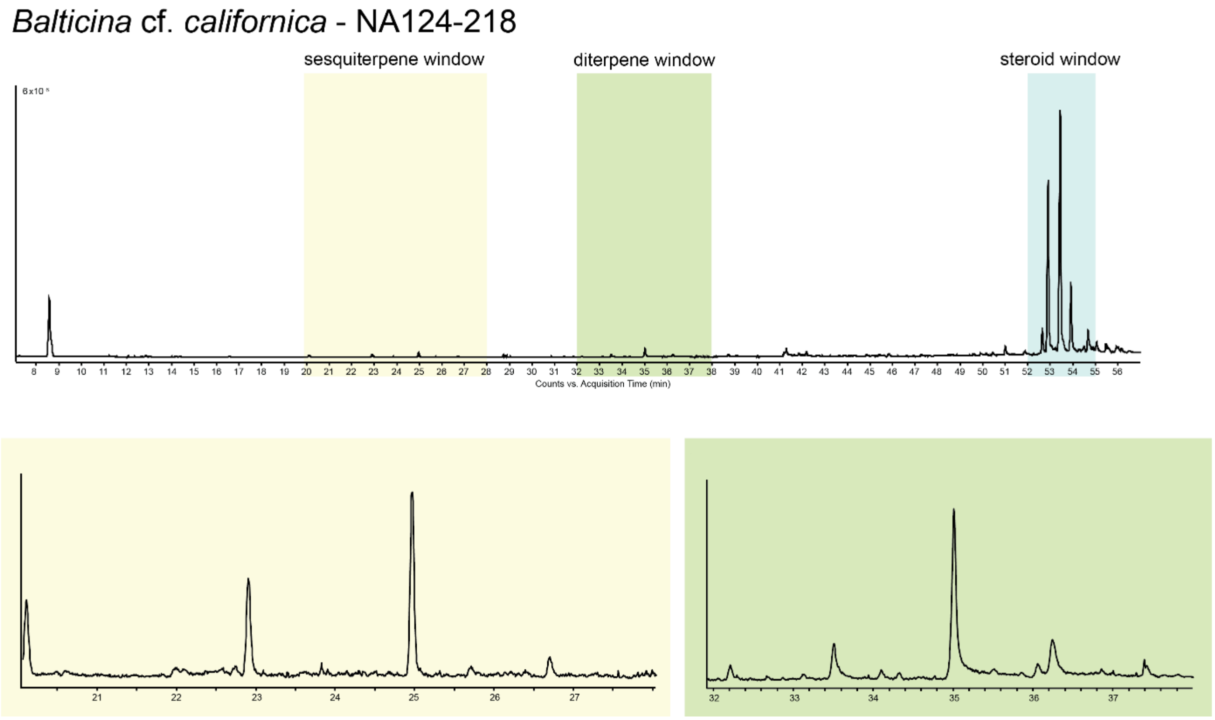

Figure S6: GCMS analysis of hexane extract derived from *Balticina* cf. *californica* NA124-218. Top: Complete TIC. Bottom: Magnified time windows for sesqui- and diterpene detection.

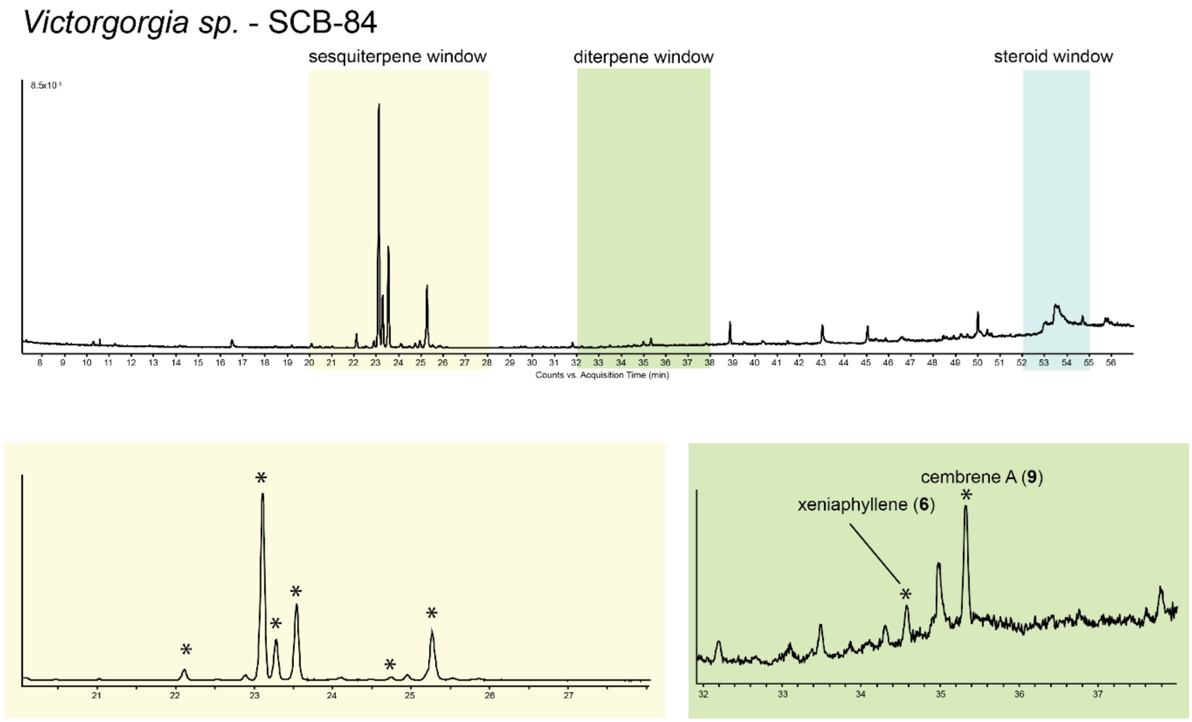

Figure S7: GCMS analysis of hexane extract derived from *Victorgorgia sp*. SCB-084. Top: Complete TIC. Bottom: Magnified time windows for sesqui- and diterpene detection. Signals labeled with asterisks represent terpene hydrocarbons.

Chrysogorgia sp. - SCB-144

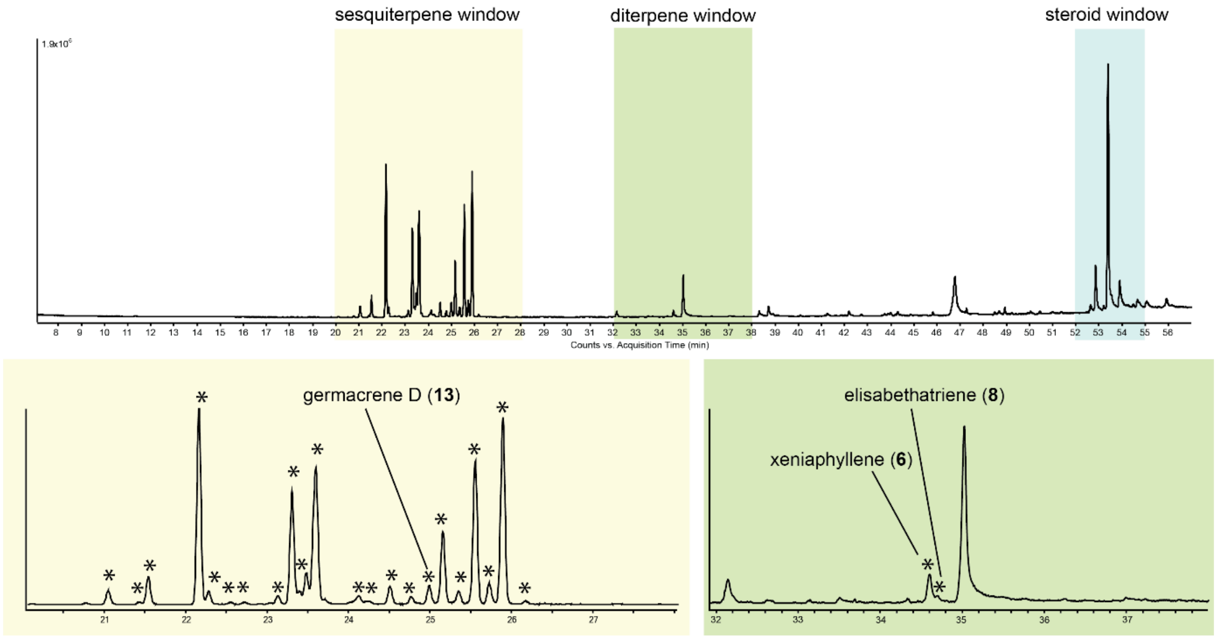

Figure S8: GCMS analysis of hexane extract derived from *Chrysogorgia sp*. SCB-144. Top: Complete TIC. Bottom: Magnified time windows for sesqui- and diterpene detection. Signals labeled with asterisks represent terpene hydrocarbons.

Chrysogorgia sp. - SCB-157

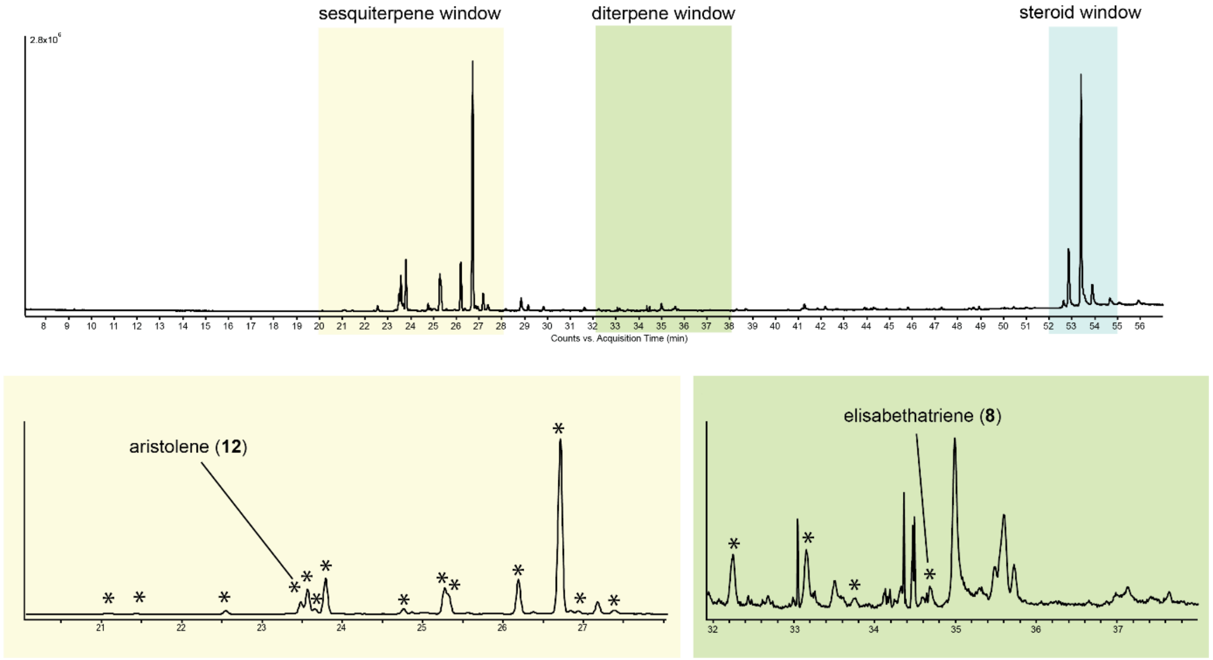

Figure S9: GCMS analysis of hexane extract derived from *Chrysogorgia sp*. SCB-157. Top: Complete TIC. Bottom: Magnified time windows for sesqui- and diterpene detection. Signals labeled with asterisks represent terpene hydrocarbons.

Paragorgia jamesi - SCB-216

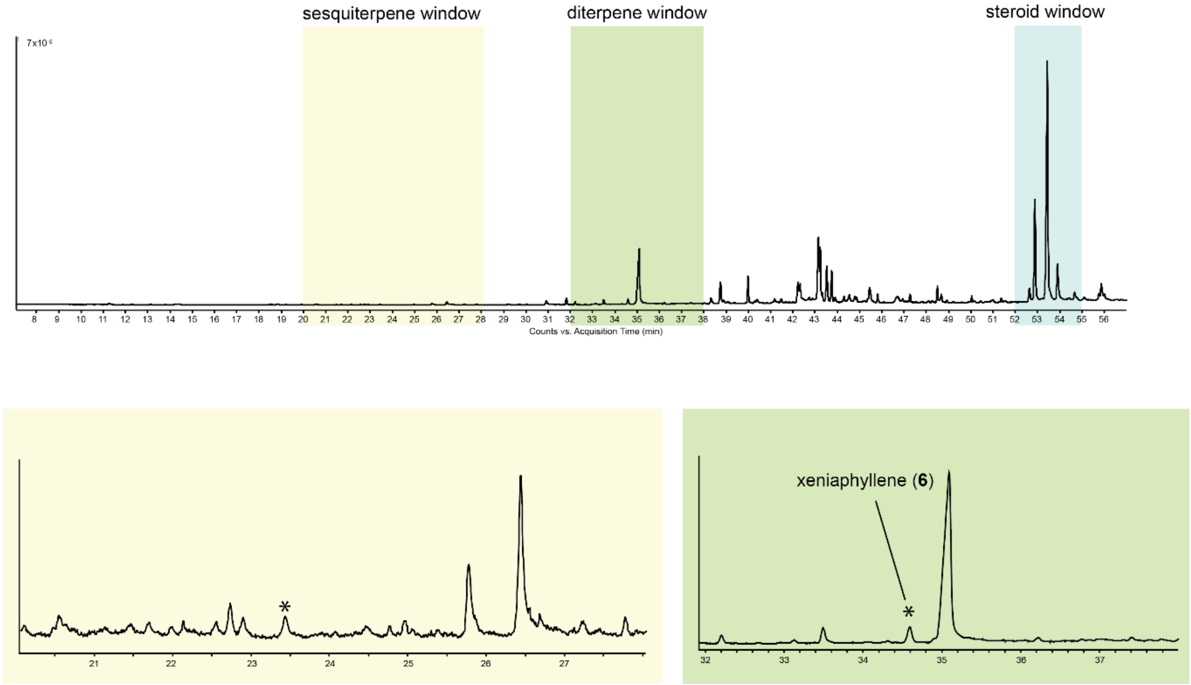

Figure S10: GCMS analysis of hexane extract derived from *Paragorgia jamesi* SCB-216. Top: Complete TIC. Bottom: Magnified time windows for sesqui- and diterpene detection. Signals labeled with asterisks represent terpene hydrocarbons.

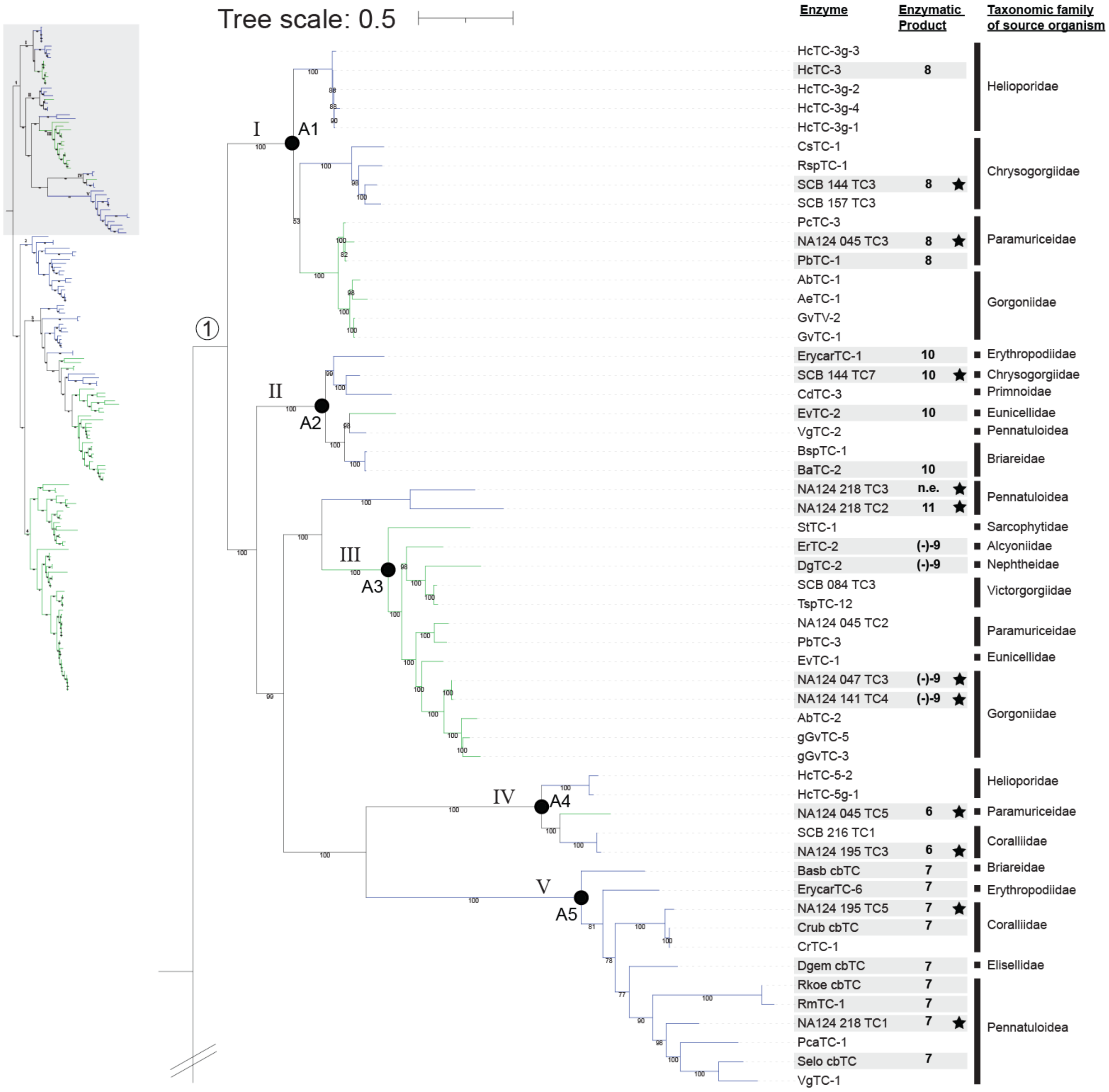

Figure S11: Phylogenetic tree of octocoral TC amino acid sequences. The four parts of the tree are labeled by numbers in circles. The subclades of part 1 are labeled with roman numbers. Green = sequences from malacalcyonaceans; blue = sequences from scleralcyonaceans. Bootstrap values are shown. The taxonomic family of the source organism is designated on the right. Enzymes characterized in this work are marked with a star. Abbreviations: m, multiple products; n.a., inactive protein; n.e., not expressed protein; ST, unknown sesquiterpene. Filled circles designate the ancestral nodes (A1-5) that were functionally characterized (Figure S35-39). The figure is continued on the next two pages.

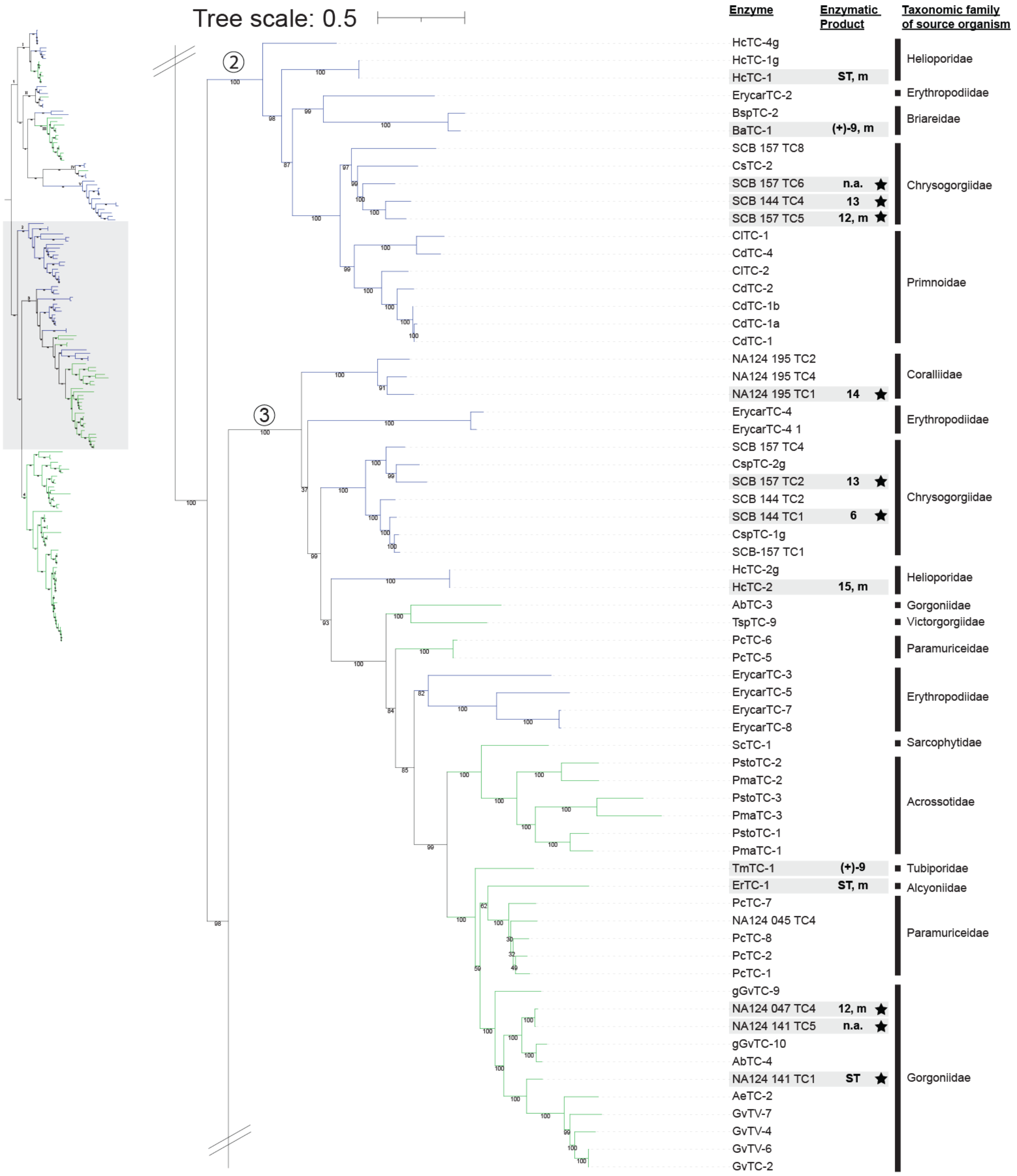

Figure S11 continued.

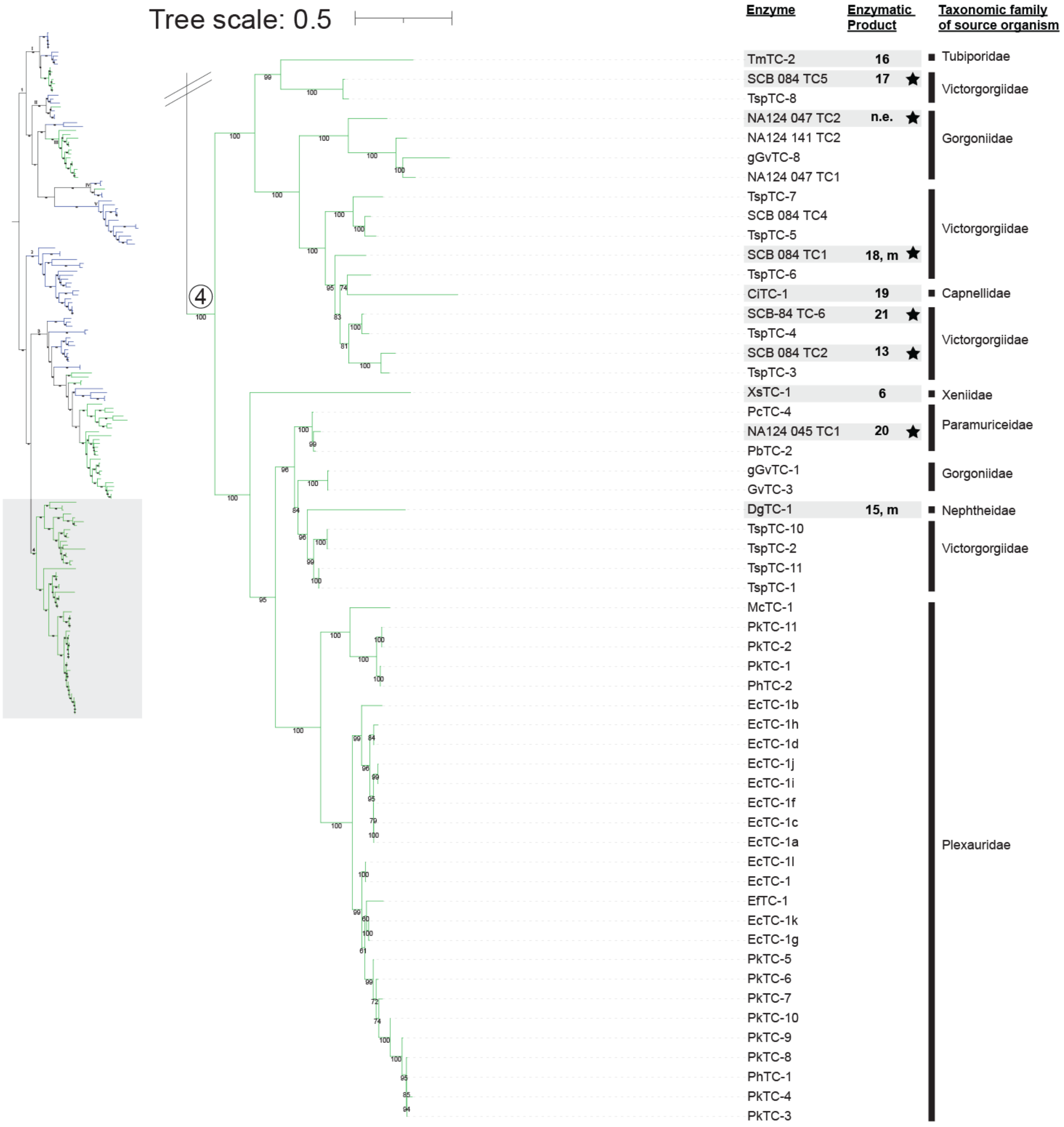

Figure S11 continued.

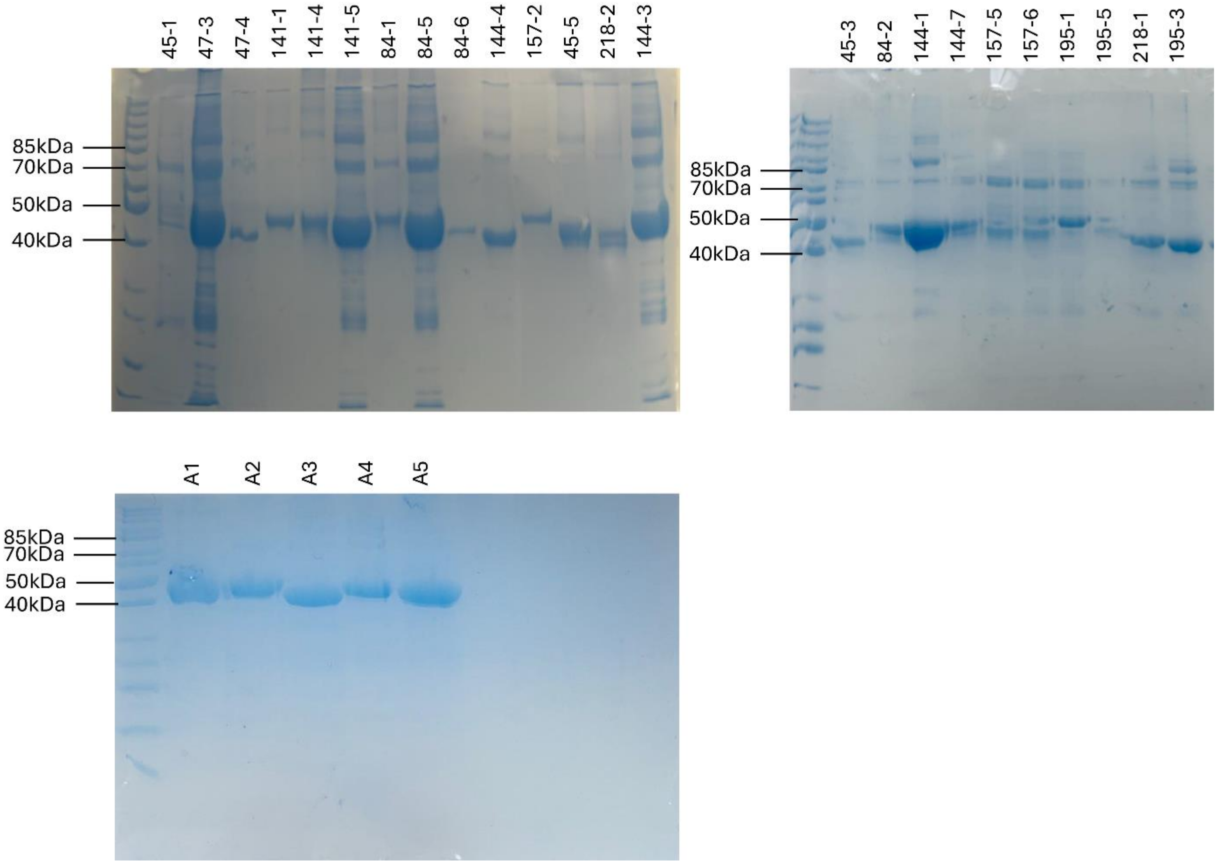

Figure 12: SDS-PAGE analysis of heterologously produced enzymes.

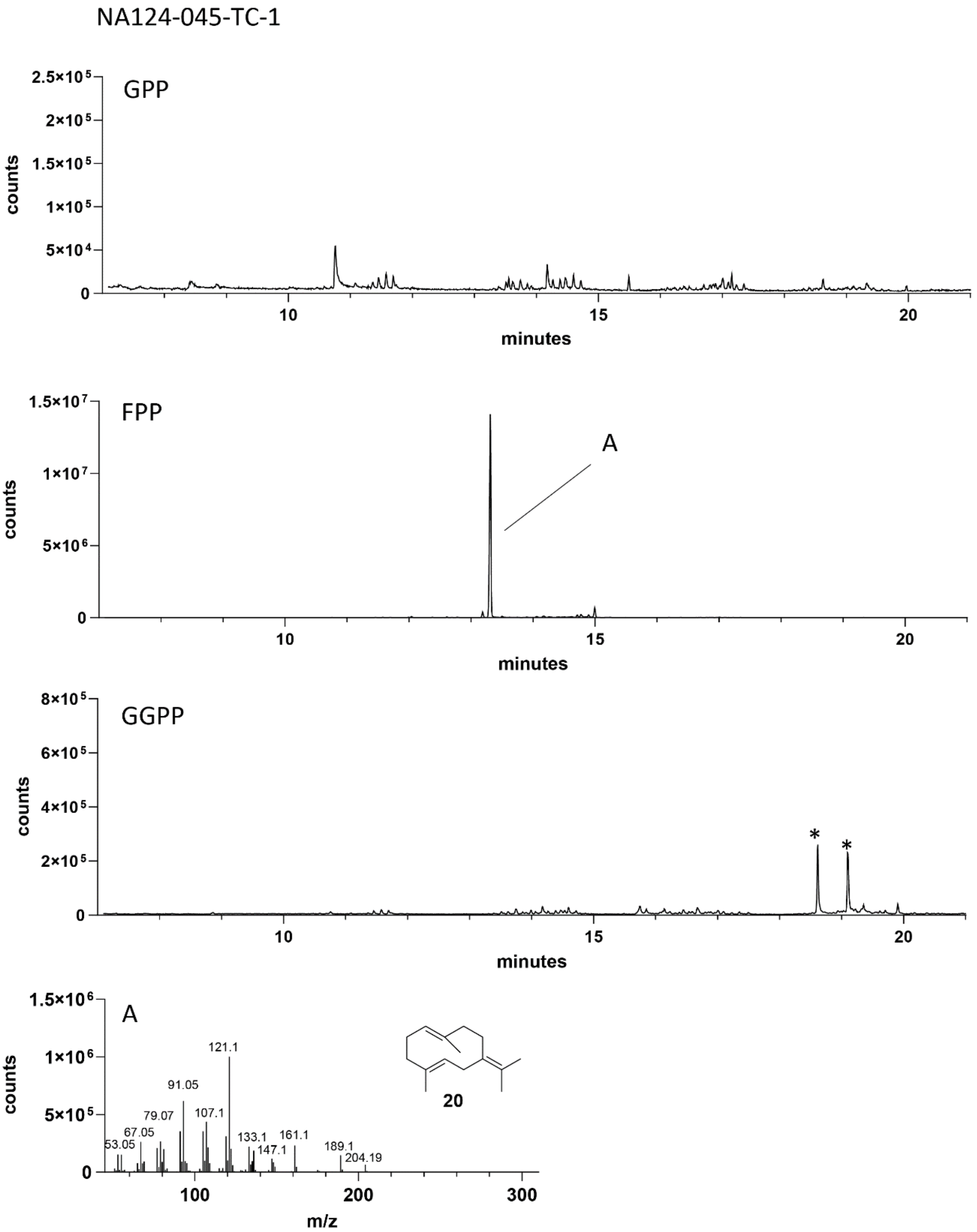

Figure S13: GCMS total ion chromatograms of NA124-045-TC-1 in vitro incubations with GPP (top), FPP (middle), and GGPP (bottom). Mass spectra of enzymatically produced terpenes are shown. Signals marked with asterisks are non-enzymatic degradation products of substrates. The identity of **20** has been verified using an authentic standard.

NA124-45-TC-3

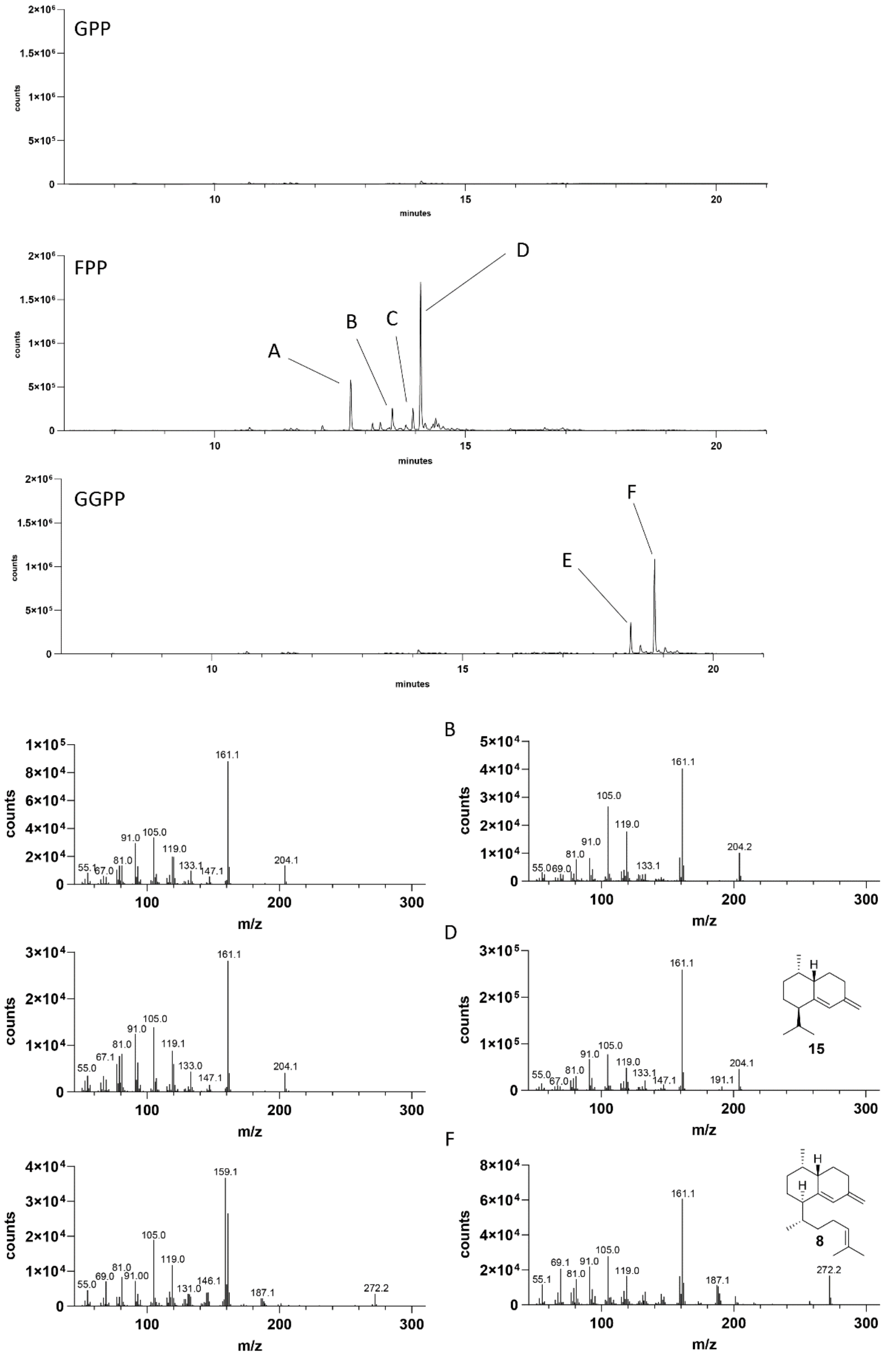

Figure S14: GCMS total ion chromatograms of NA124-045-TC-3 in vitro incubations with GPP (top), FPP (middle), and GGPP (bottom). Mass spectra of enzymatically produced terpenes are shown. The identities of **8** and **15** have been verified using authentic standards.

NA124-045-TC-5

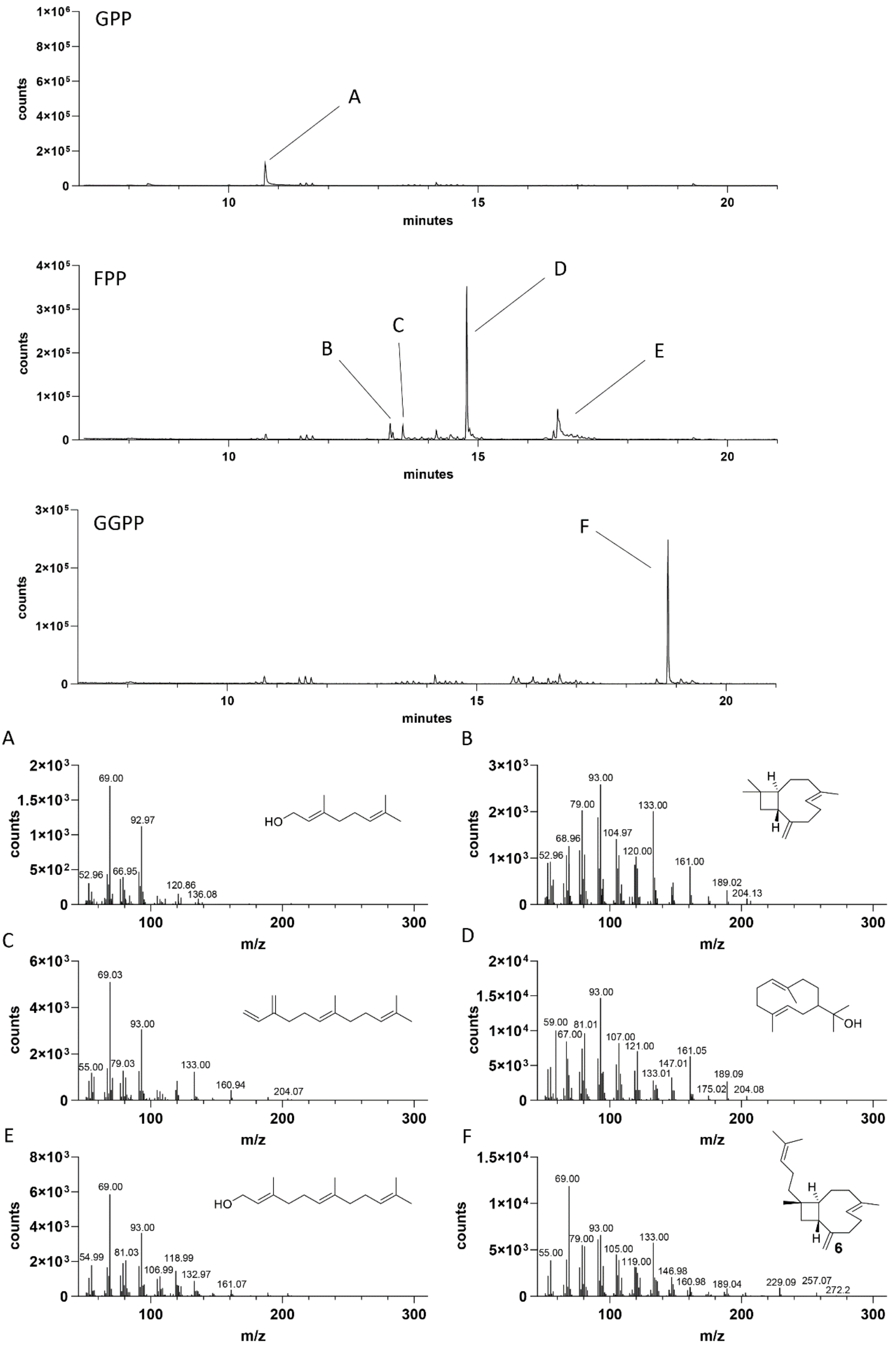

Figure S15: GCMS total ion chromatograms of NA124-045-TC-5 in vitro incubations with GPP (top), FPP (middle), and GGPP (bottom). Mass spectra of enzymatically produced terpenes are shown. **6** was verified using an authentic standard, the other shown structures are best hits from the NIST database.

NA124-047-TC-3

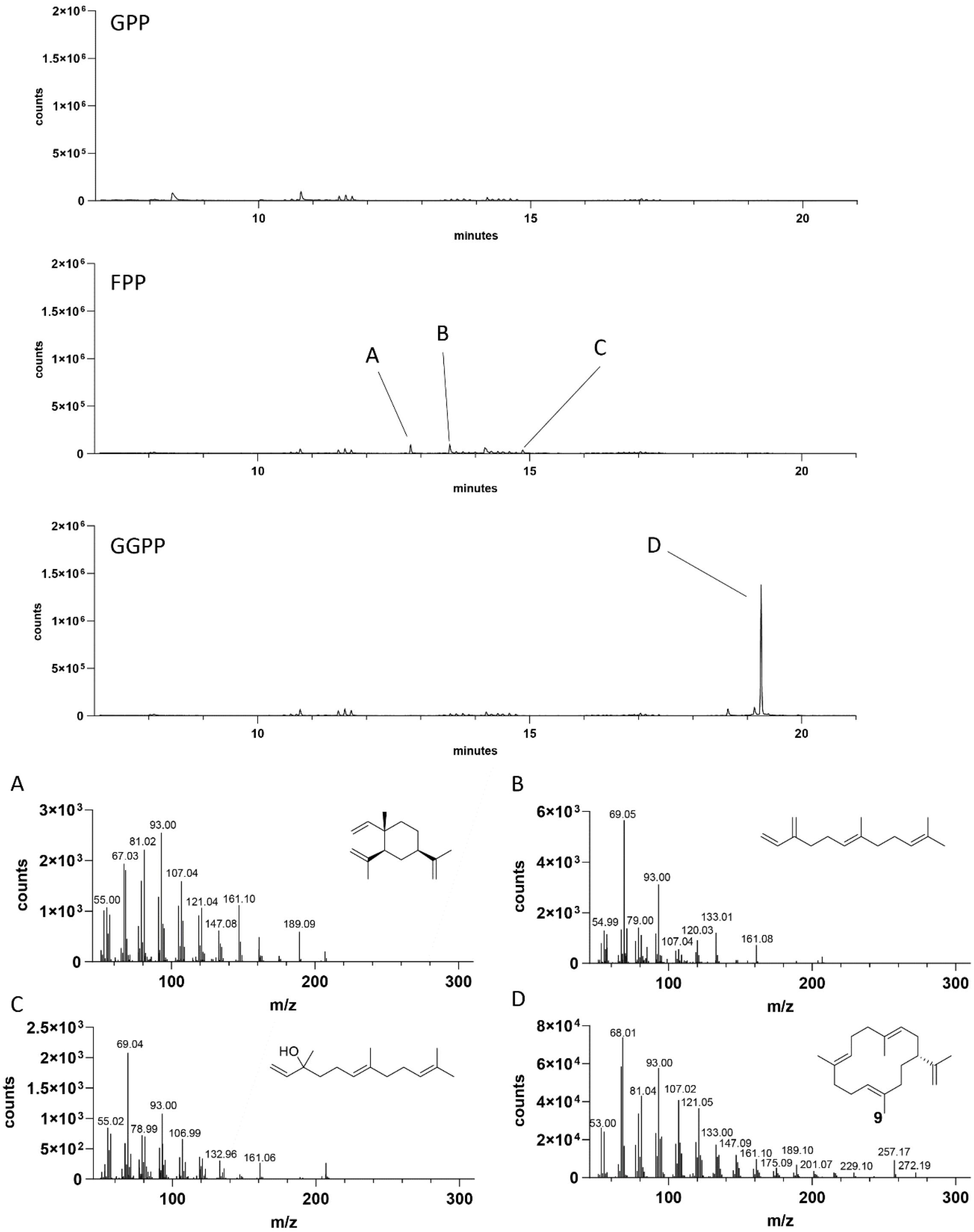

Figure S16: GCMS total ion chromatograms of NA124-047-TC-3 in vitro incubations with GPP (top), FPP (middle), and GGPP (bottom). Mass spectra of enzymatically produced terpenes are shown. **9** was verified using an authentic standard, the other shown structures are best hits from the NIST database.

NA124-47-TC-4

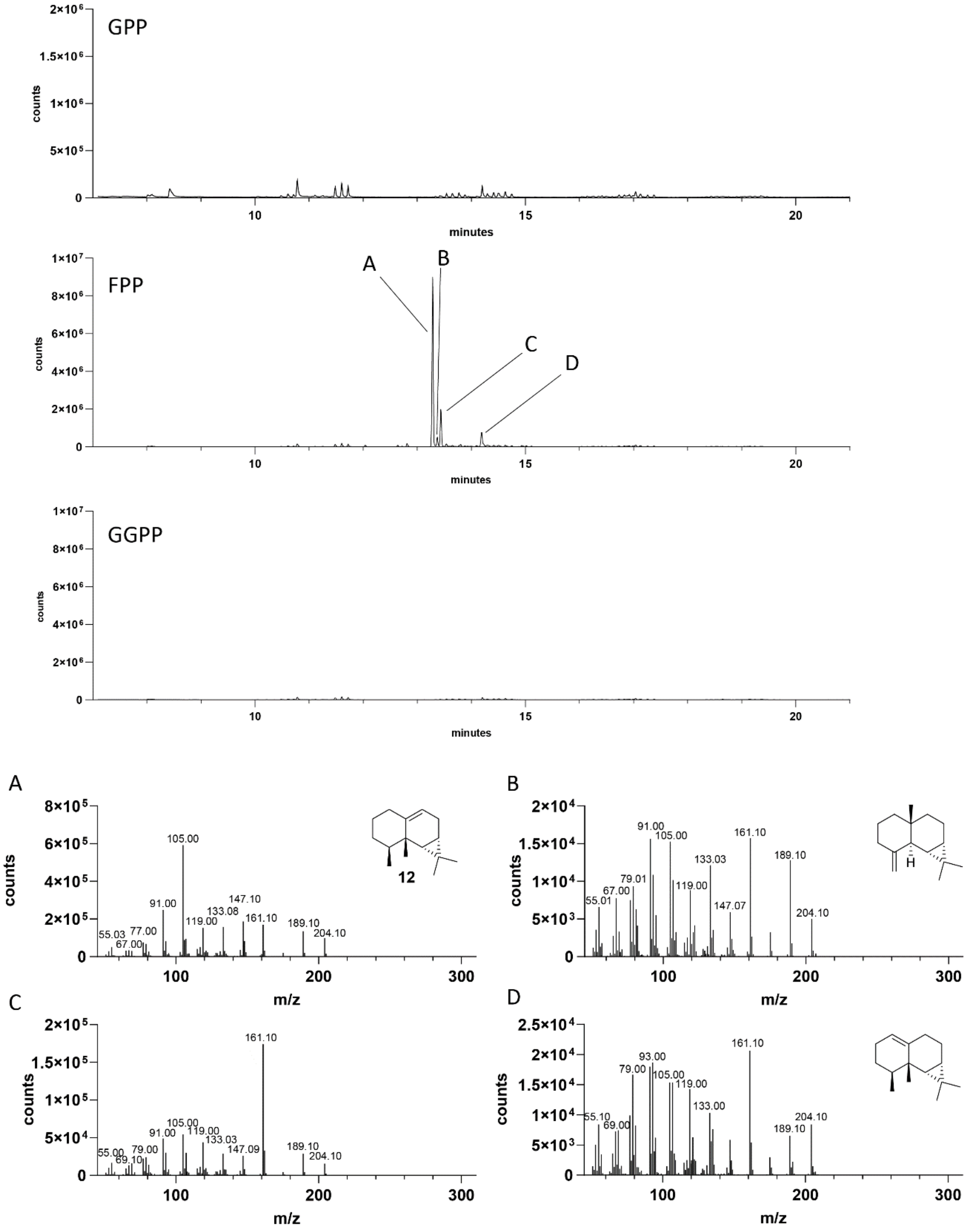

Figure S17: GCMS total ion chromatograms of NA124-047-TC-4 in vitro incubations with GPP (top), FPP (middle), and GGPP (bottom). Mass spectra of enzymatically produced terpenes are shown. **12** was verified using an authentic standard, the other shown structures are best hits from the NIST database.

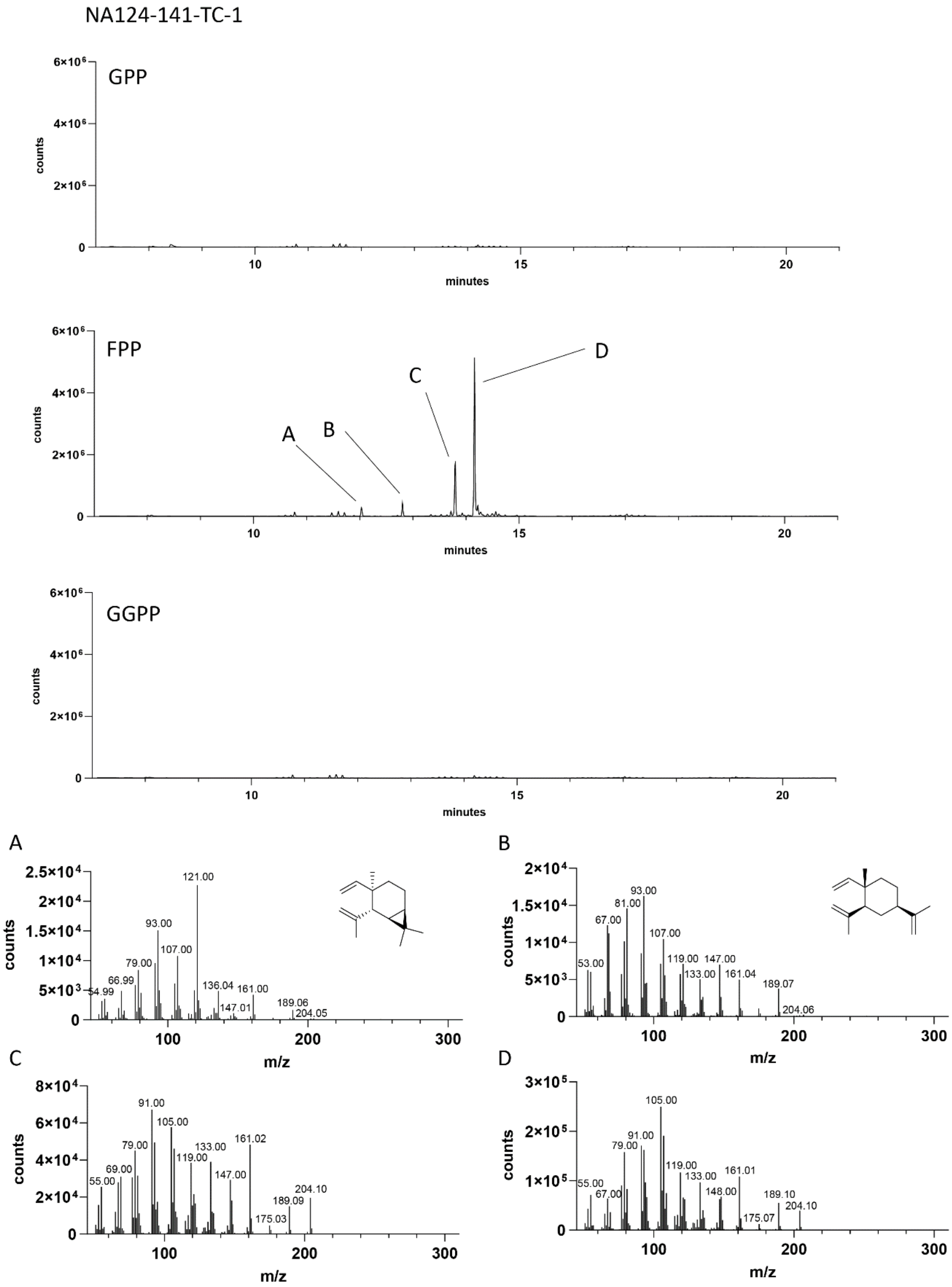

Figure S18: GCMS total ion chromatograms of NA124-141-TC-1 in vitro incubations with GPP (top), FPP (middle), and GGPP (bottom). Mass spectra of enzymatically produced terpenes are shown. The shown structures are best hits from the NIST database.

NA124-141-TC-4

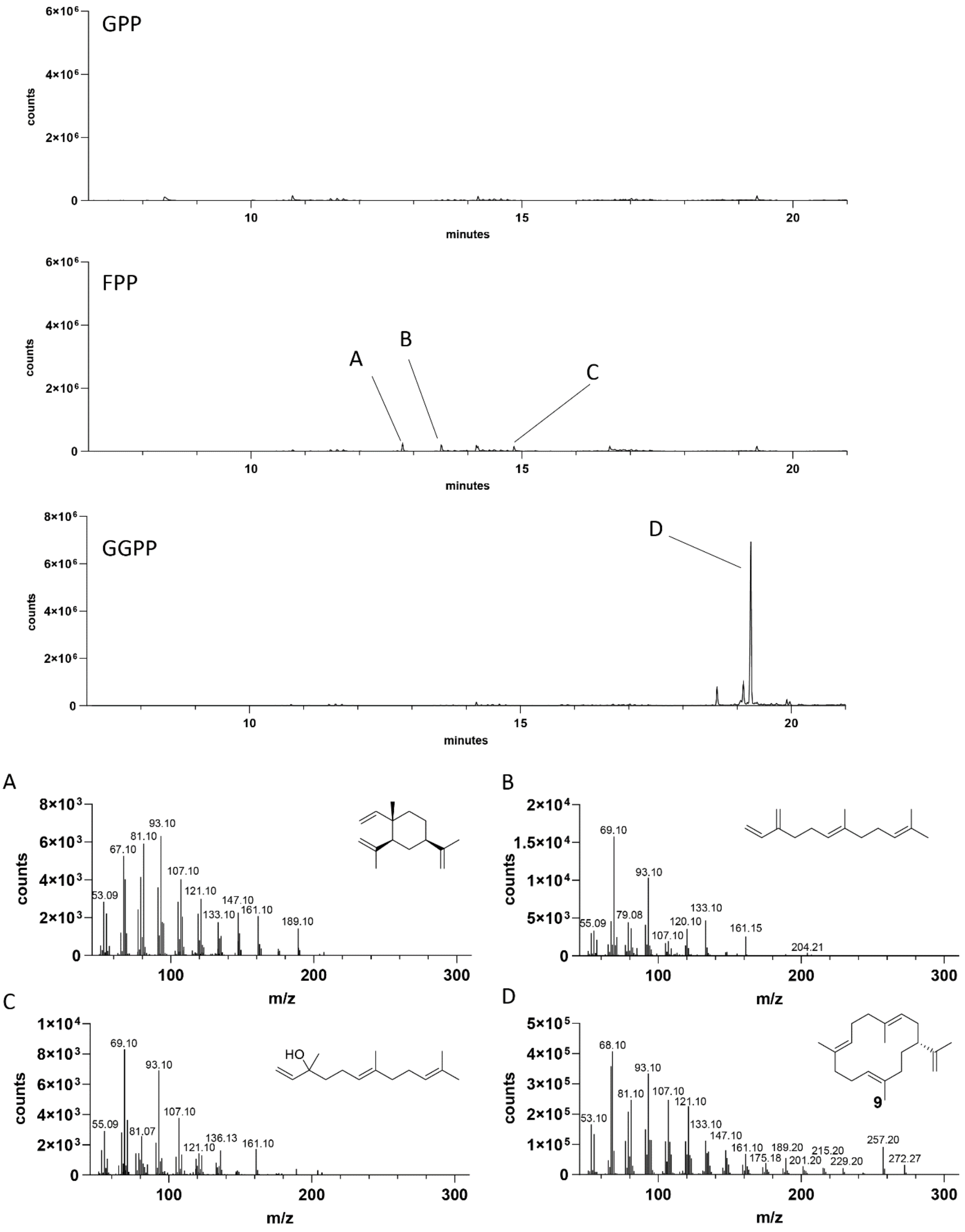

Figure S19: GCMS total ion chromatograms of NA124-141-TC-4 in vitro incubations with GPP (top), FPP (middle), and GGPP (bottom). Mass spectra of enzymatically produced terpenes are shown. Mass spectra of enzymatically produced terpenes are shown. **9** was verified using an authentic standard, the other shown structures are best hits from the NIST database.

NA124-195-TC-1

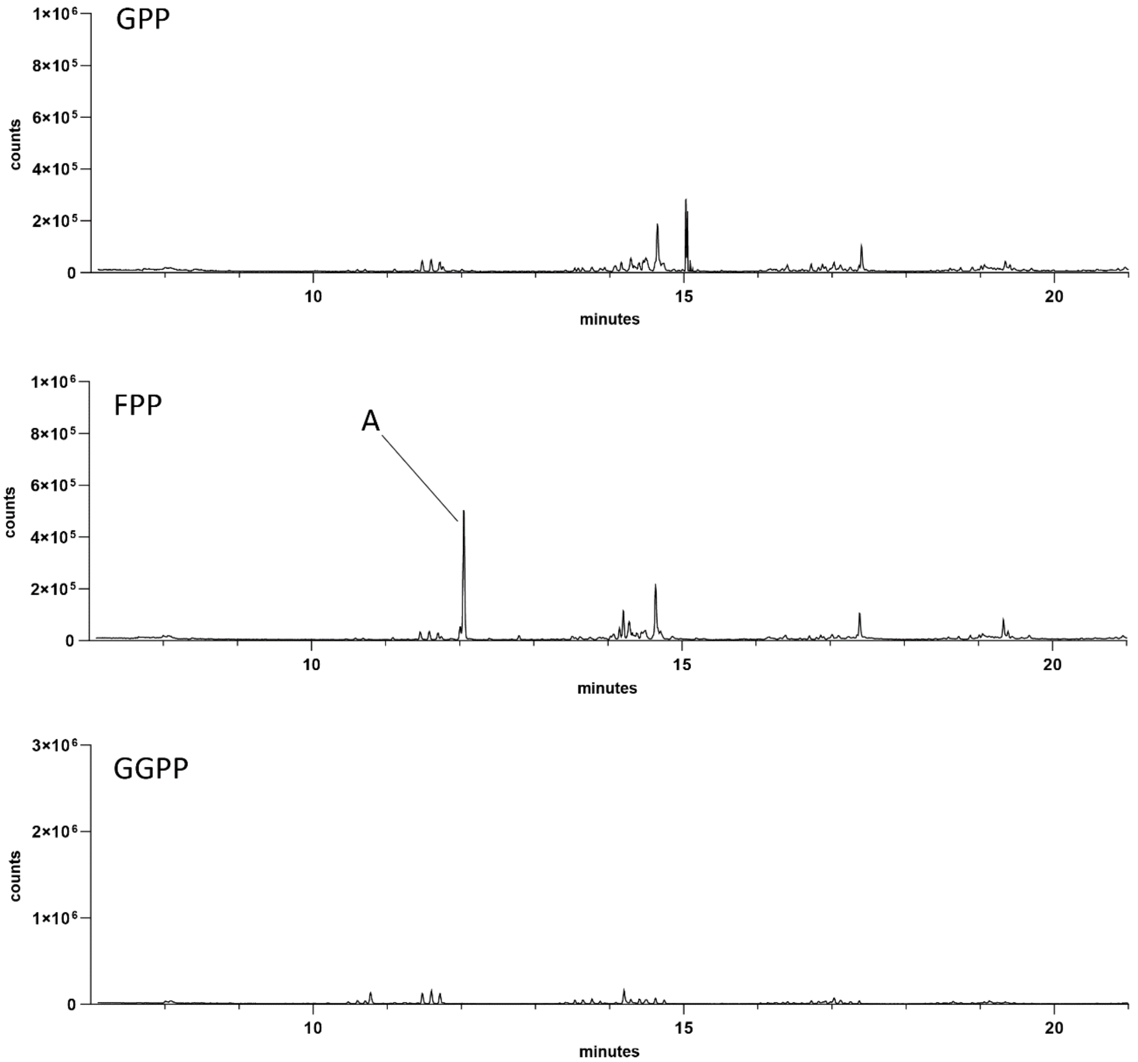

A

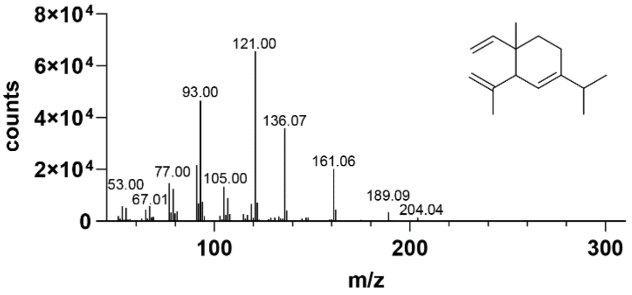

Figure S20: GCMS total ion chromatograms of NA124-15-TC-1 in vitro incubations with GPP (top), FPP (middle), and GGPP (bottom). Mass spectra of enzymatically produced terpenes are shown. The structure of compound A (d-elemene) was determined by a NIST database hit and is the thermal cope rearrangement product of the enzymatically produced compound germacrene C (**14**).

NA124-195-TC-3

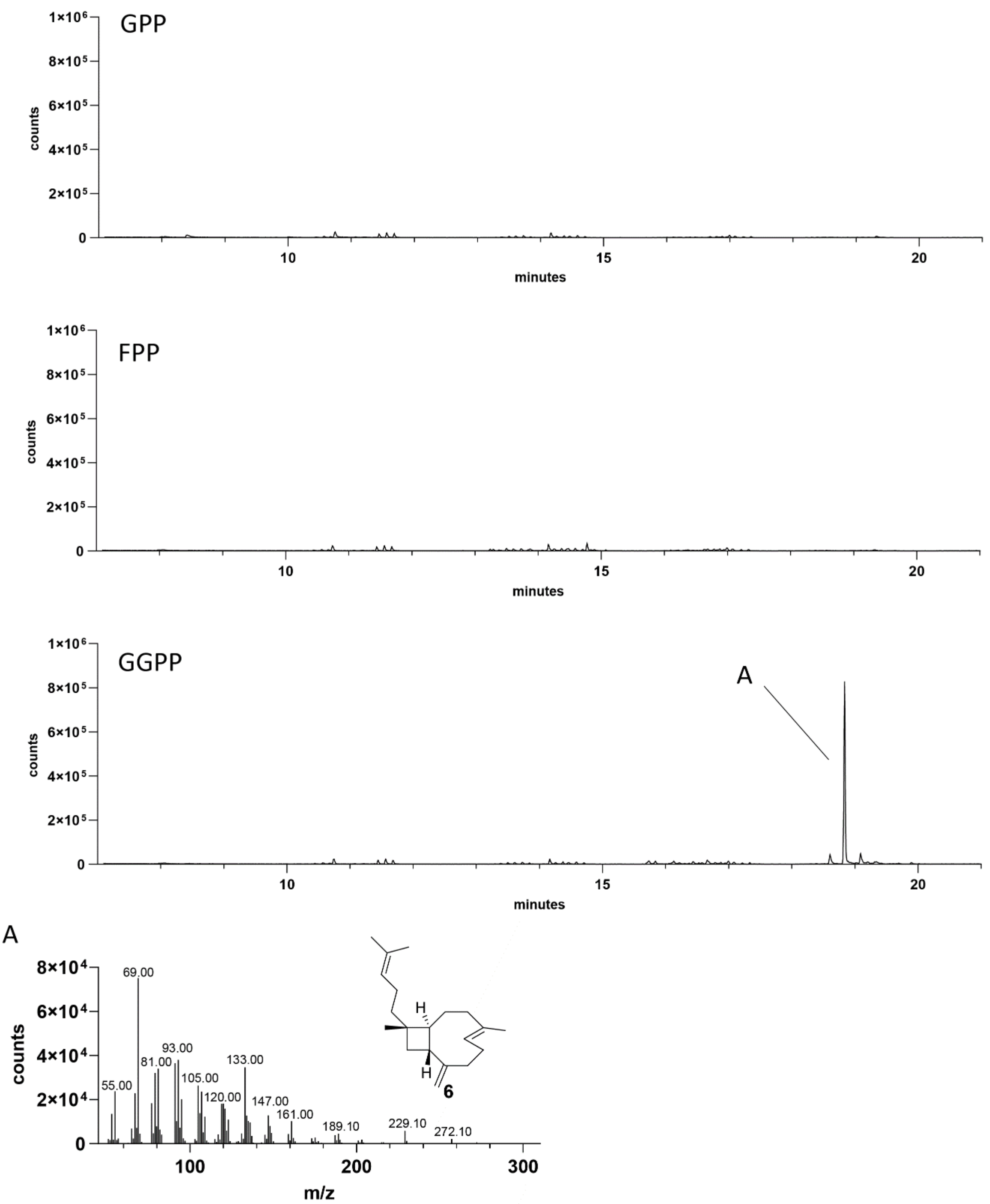

Figure S21: GCMS total ion chromatograms of NA124-195-TC-3 in vitro incubations with GPP (top), FPP (middle), and GGPP (bottom). Mass spectra of enzymatically produced terpenes are shown. **6** was verified using an authentic standard.

NA124-195-TC-5

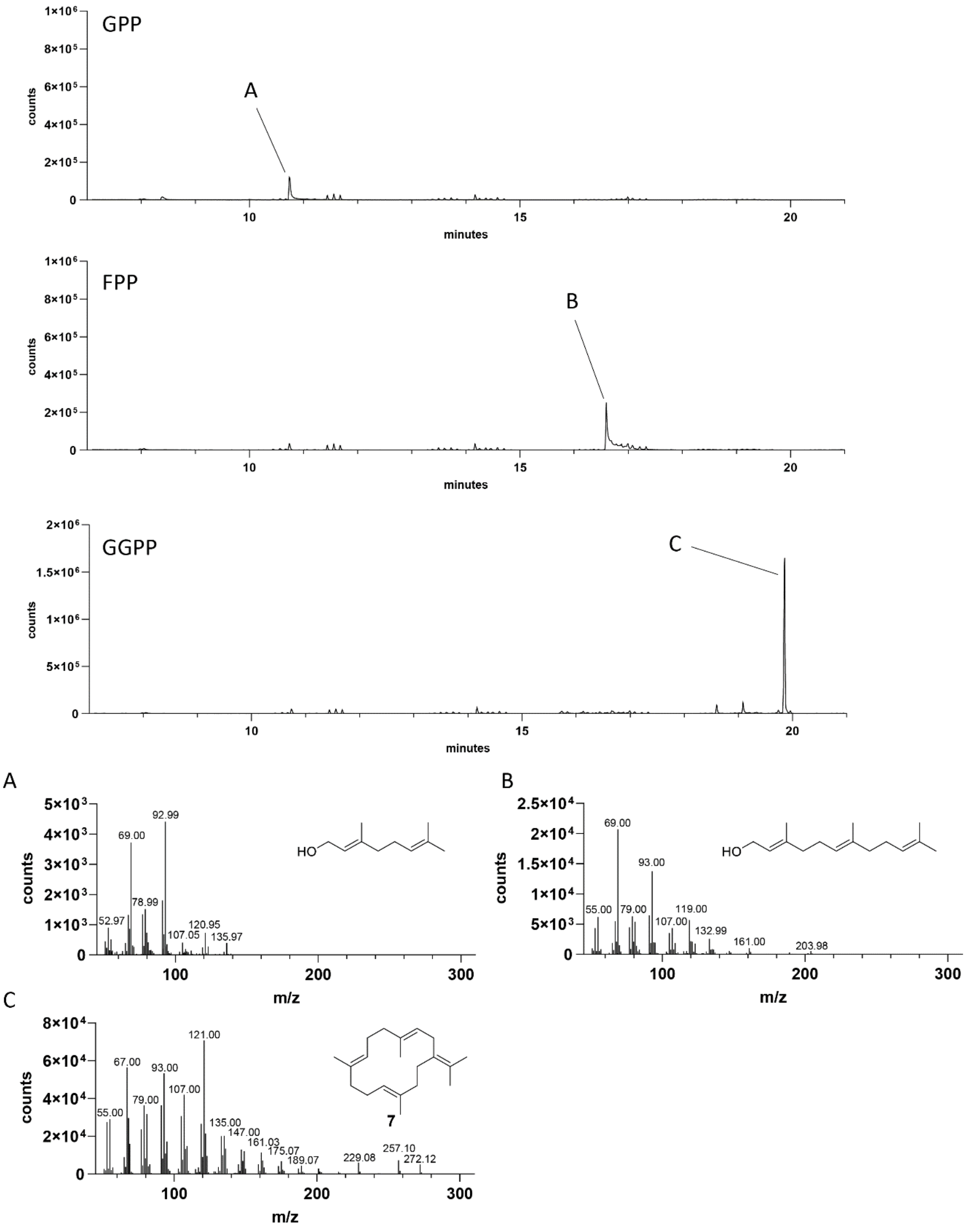

Figure S22: GCMS total ion chromatograms of NA124-195-TC-5 in vitro incubations with GPP (top), FPP (middle), and GGPP (bottom). Mass spectra of enzymatically produced terpenes are shown. **7** was verified using an authentic standard, the other shown structures are best hits from the NIST database.

SCB-84-TC-1

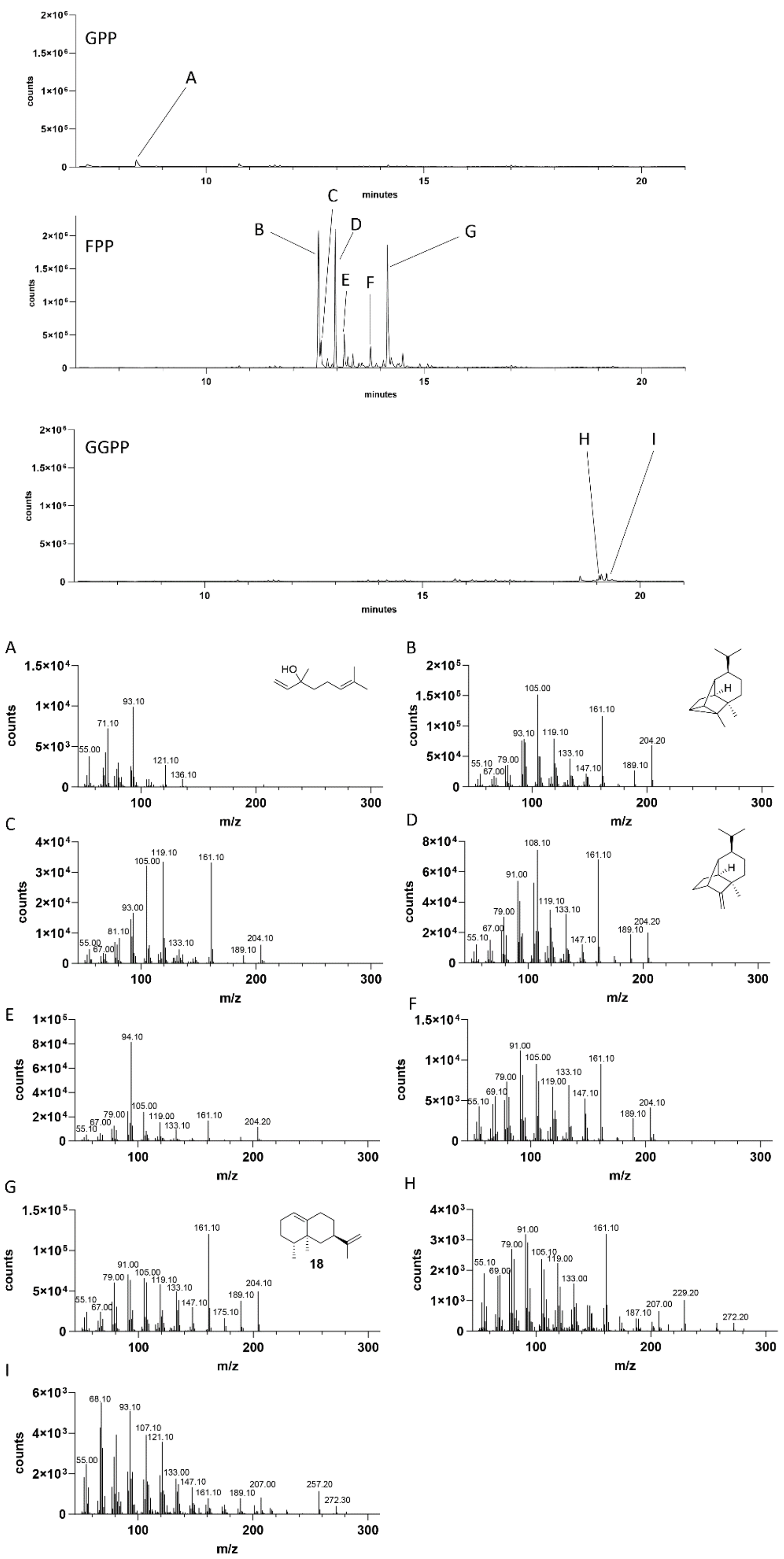

Figure S23: GCMS total ion chromatograms of SCB-084-TC-1 in vitro incubations with GPP (top), FPP (middle), and GGPP (bottom). Mass spectra of enzymatically produced terpenes are shown. **18** was verified using an authentic standard, the other shown structures are best hits from the NIST database.

SCB-84-TC-2

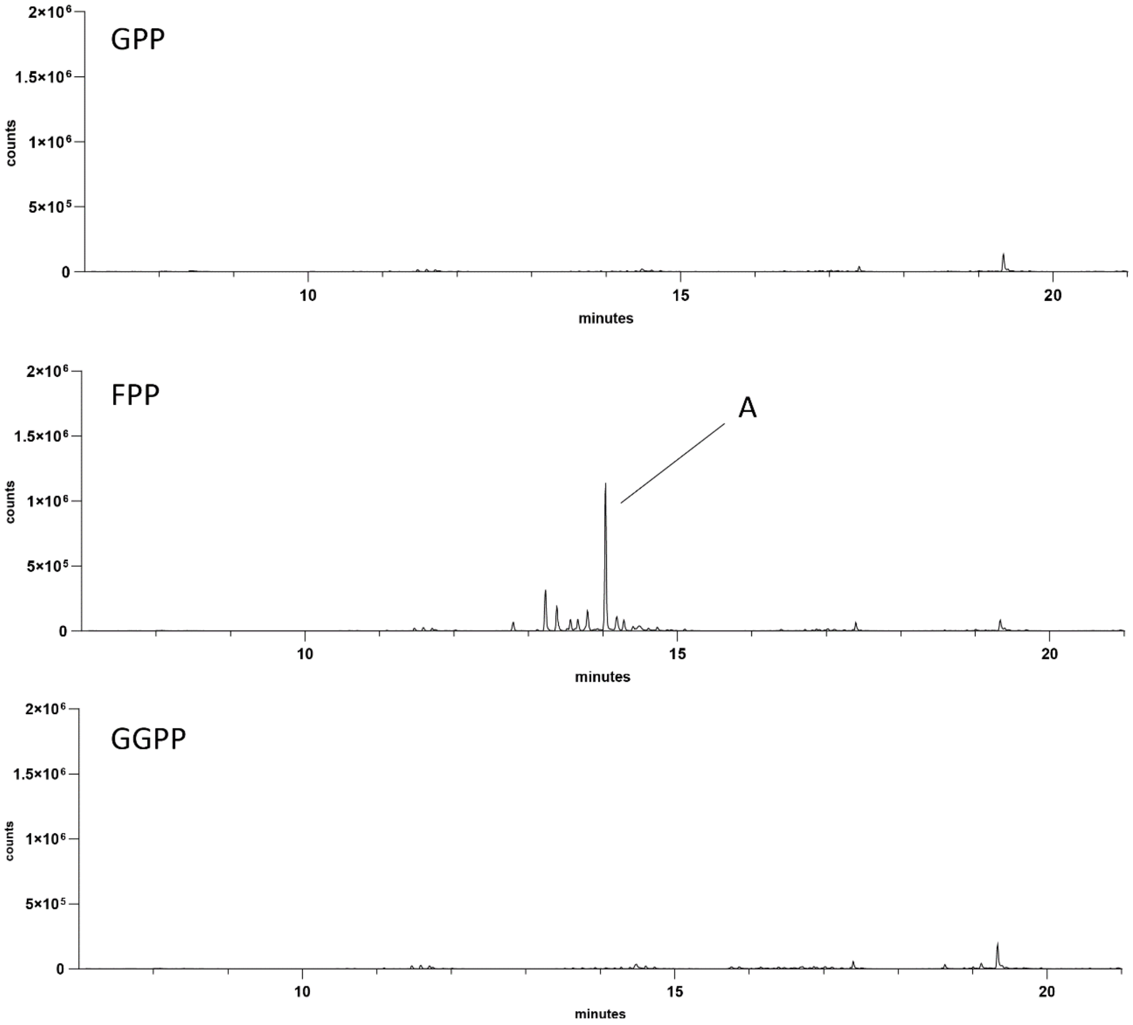

A

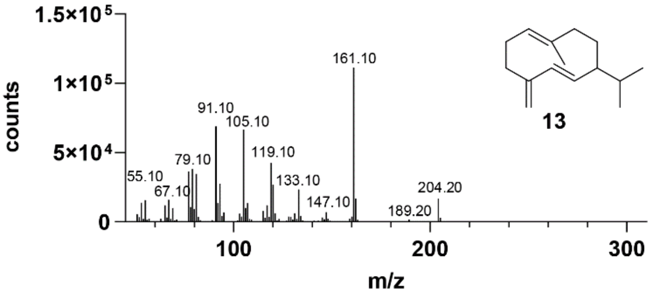

Figure S24: GCMS total ion chromatograms of SCB-084-TC-2 in vitro incubations with GPP (top), FPP (middle), and GGPP (bottom). Mass spectra of enzymatically produced terpenes are shown. **13** was verified using an authentic standard.

SCB-84-TC-5

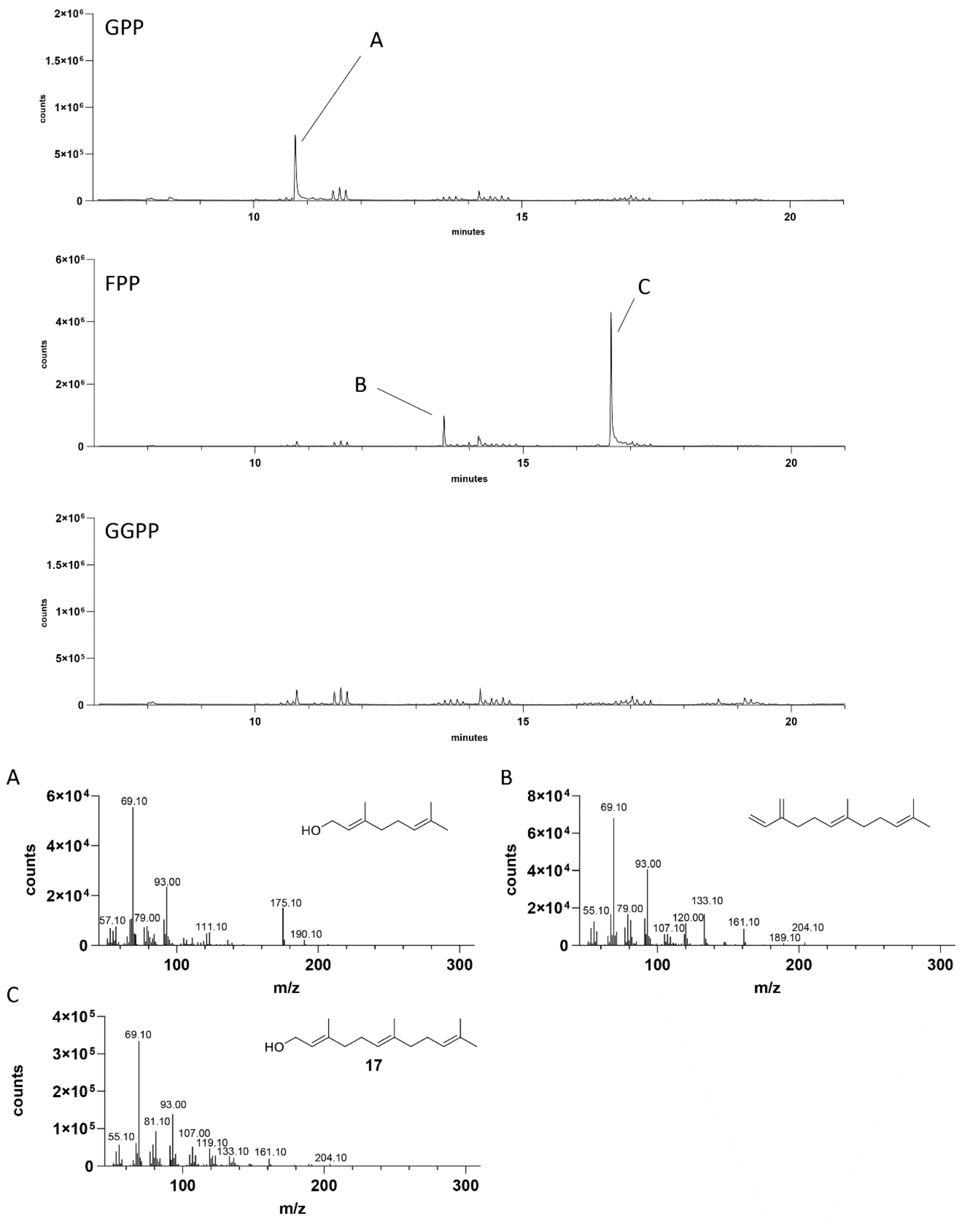

Figure S25: GCMS total ion chromatograms of SCB-084-TC-5 in vitro incubations with GPP (top), FPP (middle), and GGPP (bottom). Mass spectra of enzymatically produced terpenes are shown. **17** and the other shown structures are best hits from the NIST database.

SCB-84-TC-6

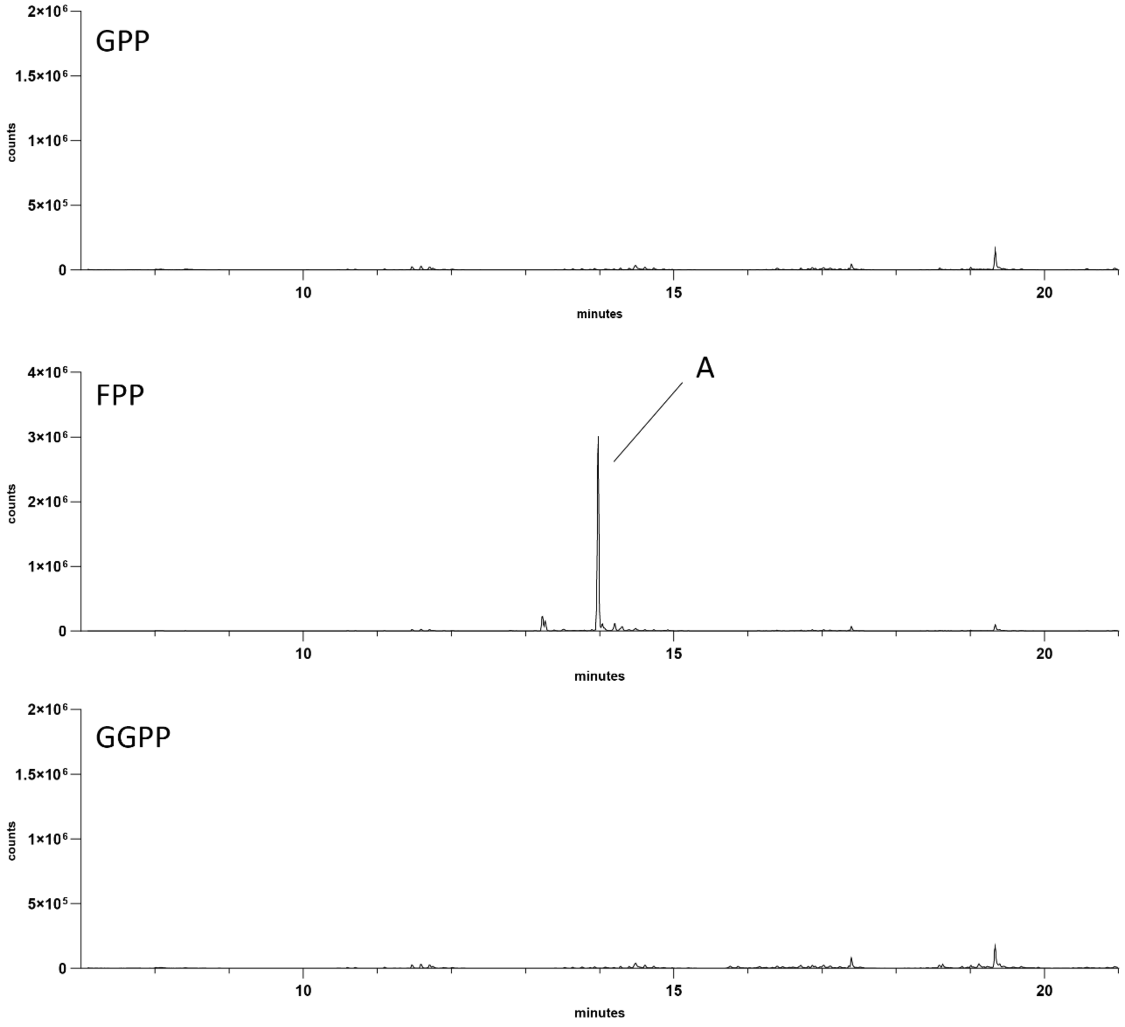

A

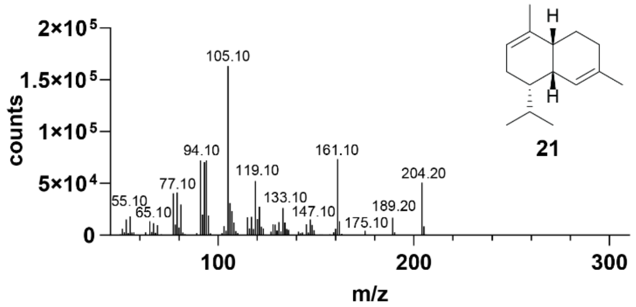

Figure S26: GCMS total ion chromatograms of SCB-084-TC-6 in vitro incubations with GPP (top), FPP (middle), and GGPP (bottom). Mass spectra of enzymatically produced terpenes are shown. The structure of **21** was verified by NMR structure elucidation of purified compound from a large-scale in-vitro assay.

SCB-144-TC-1

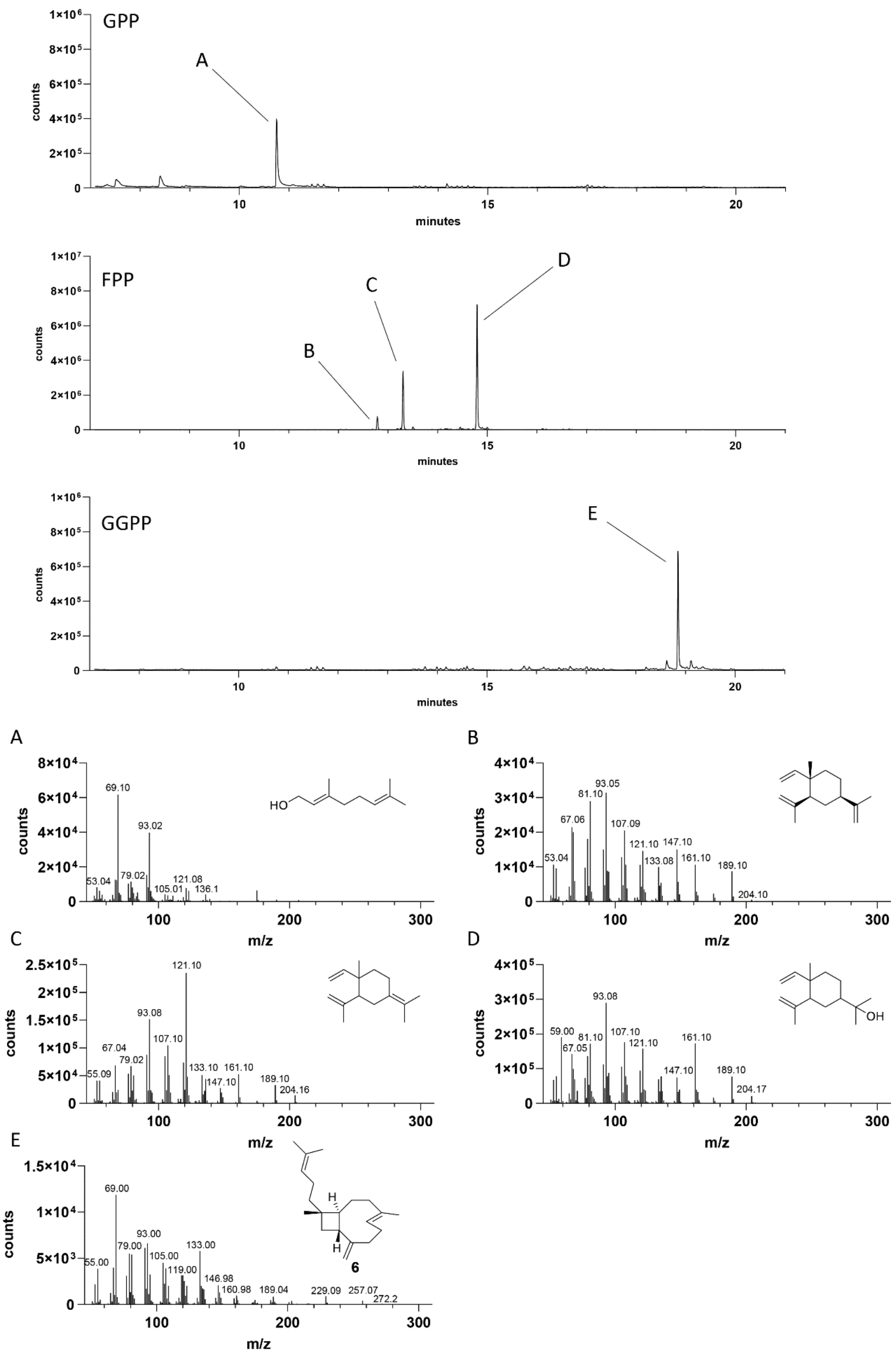

Figure S27: GCMS total ion chromatograms of SCB-144-TC-1 in vitro incubations with GPP (top), FPP (middle), and GGPP (bottom). Mass spectra of enzymatically produced terpenes are shown. **6** was verified using an authentic standard, the other shown structures are best hits from the NIST database.

SCB-144-TC-3

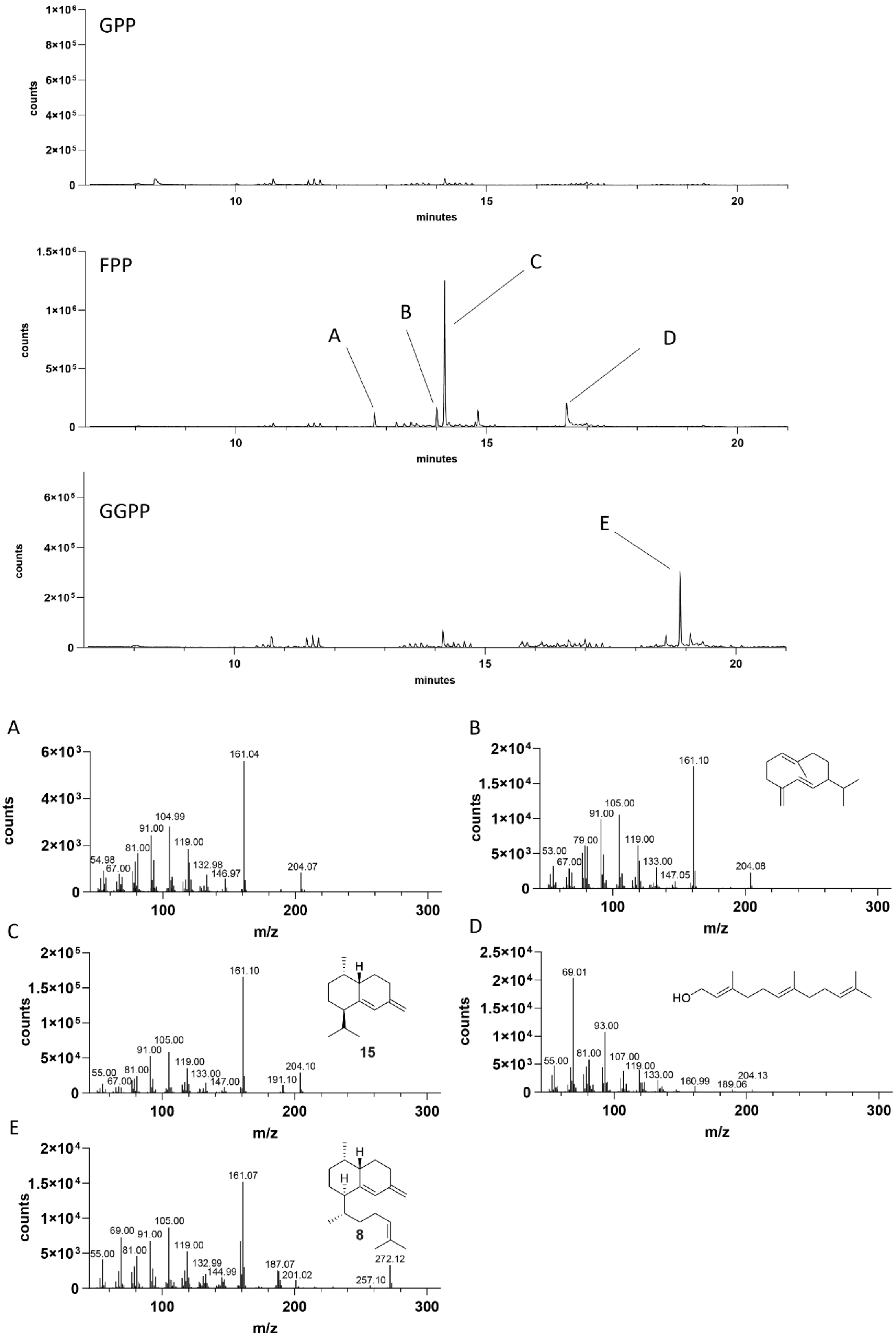

Figure S28: GCMS total ion chromatograms of SCB-144-TC-3 in vitro incubations with GPP (top), FPP (middle), and GGPP (bottom). Mass spectra of enzymatically produced terpenes are shown. The identity of **8** and **15** has been verified using authentic standards.

SCB-144-TC-4

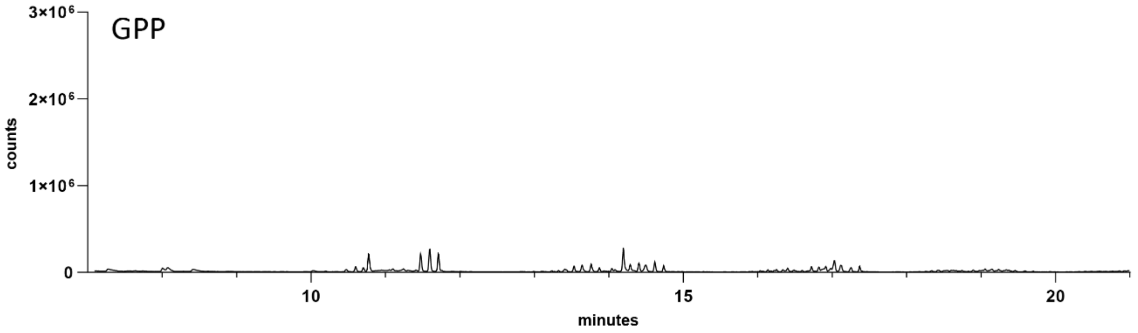

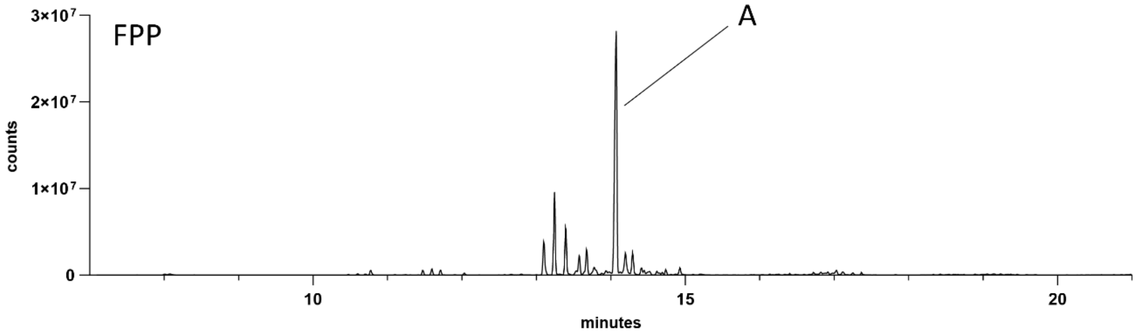

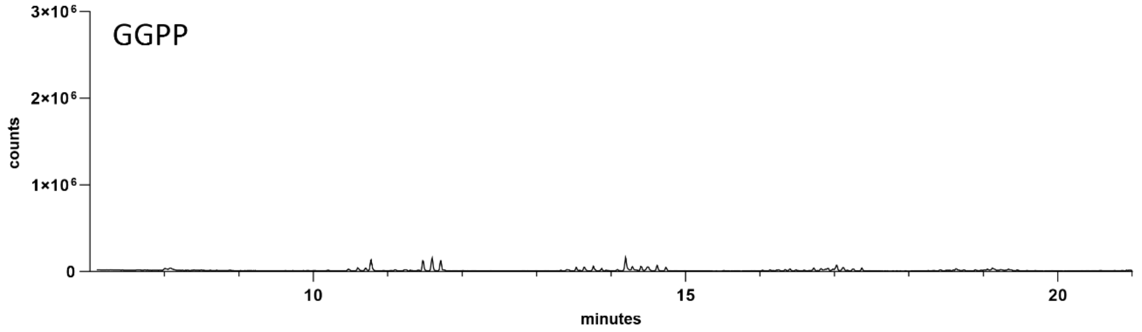

A

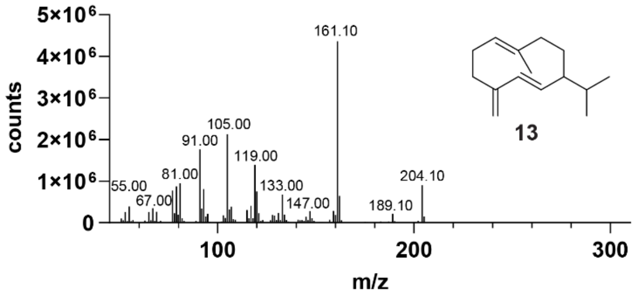

Figure S29: GCMS total ion chromatograms of SCB-144-TC-4 in vitro incubations with GPP (top), FPP (middle), and GGPP (bottom). Mass spectra of enzymatically produced terpenes are shown. The structure of **13** has been verified using an authentic standard.

SCB-144-TC-7

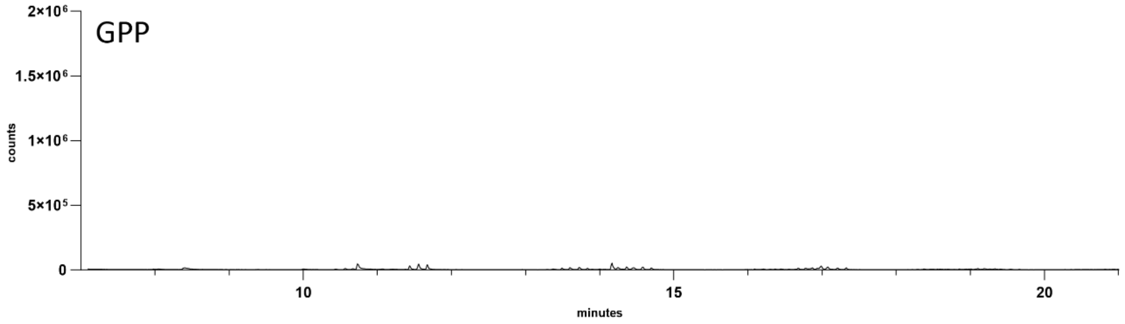

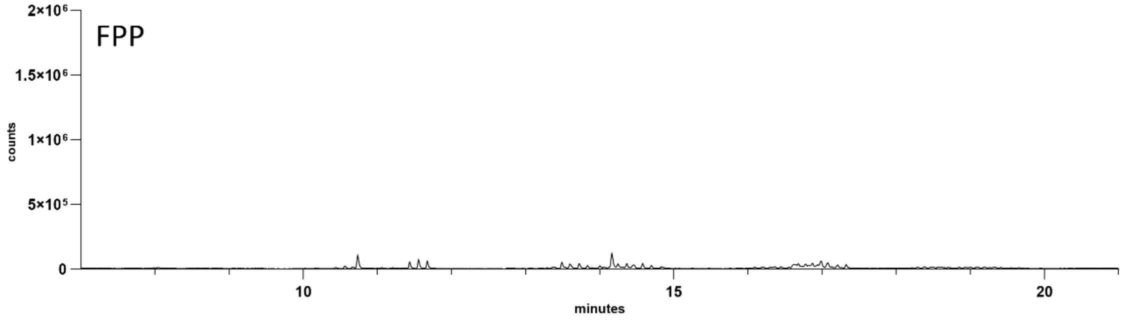

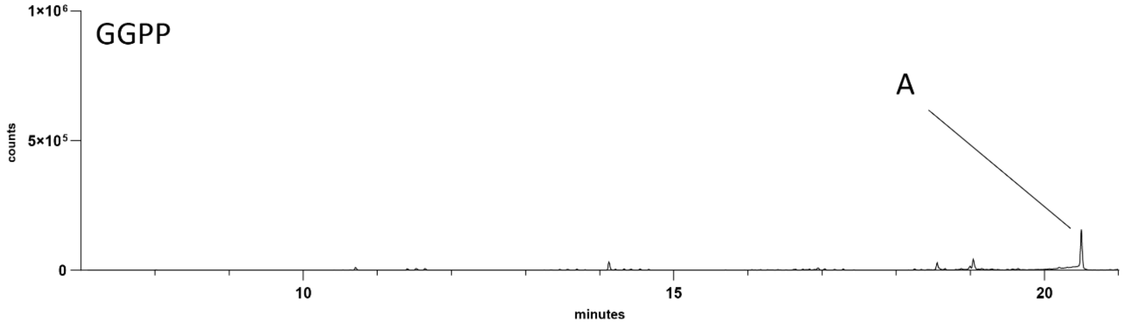

A

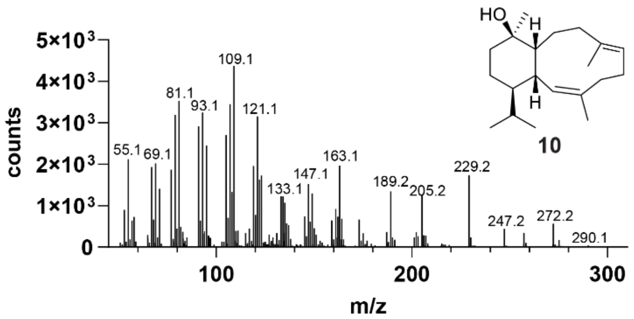

Figure S30: GCMS total ion chromatograms of SCB-144-TC-7 in vitro incubations with GPP (top), FPP (middle), and GGPP (bottom). Mass spectra of enzymatically produced terpenes are shown. The identity of **10** was verified using an authentic standard.

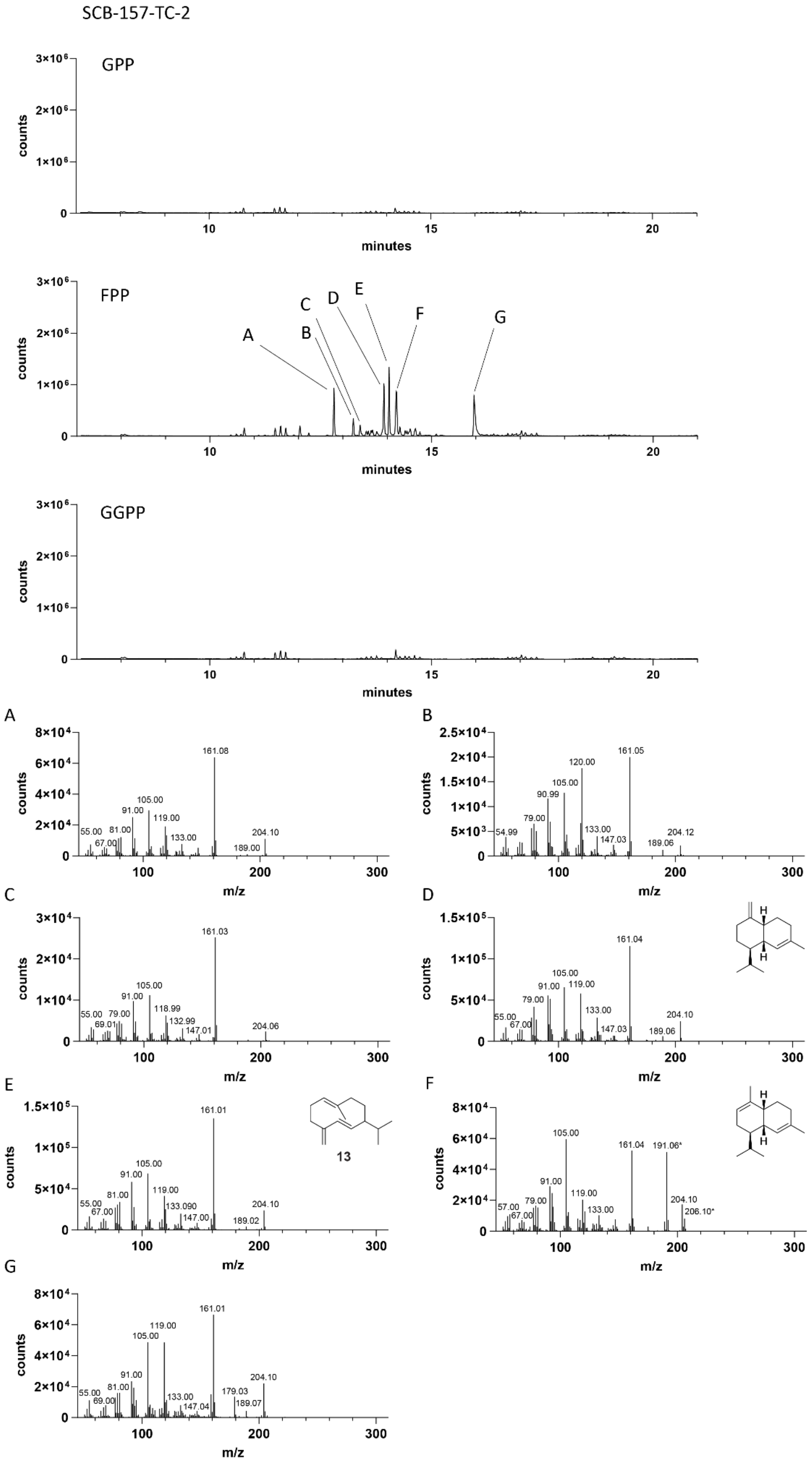

Figure S31: GCMS total ion chromatograms of SCB-157-TC-2 in vitro incubations with GPP (top), FPP (middle), and GGPP (bottom). Mass spectra of enzymatically produced terpenes are shown. The identity of **13** was proven using an authentic standard, the other shown structures are best hits from the NIST database.

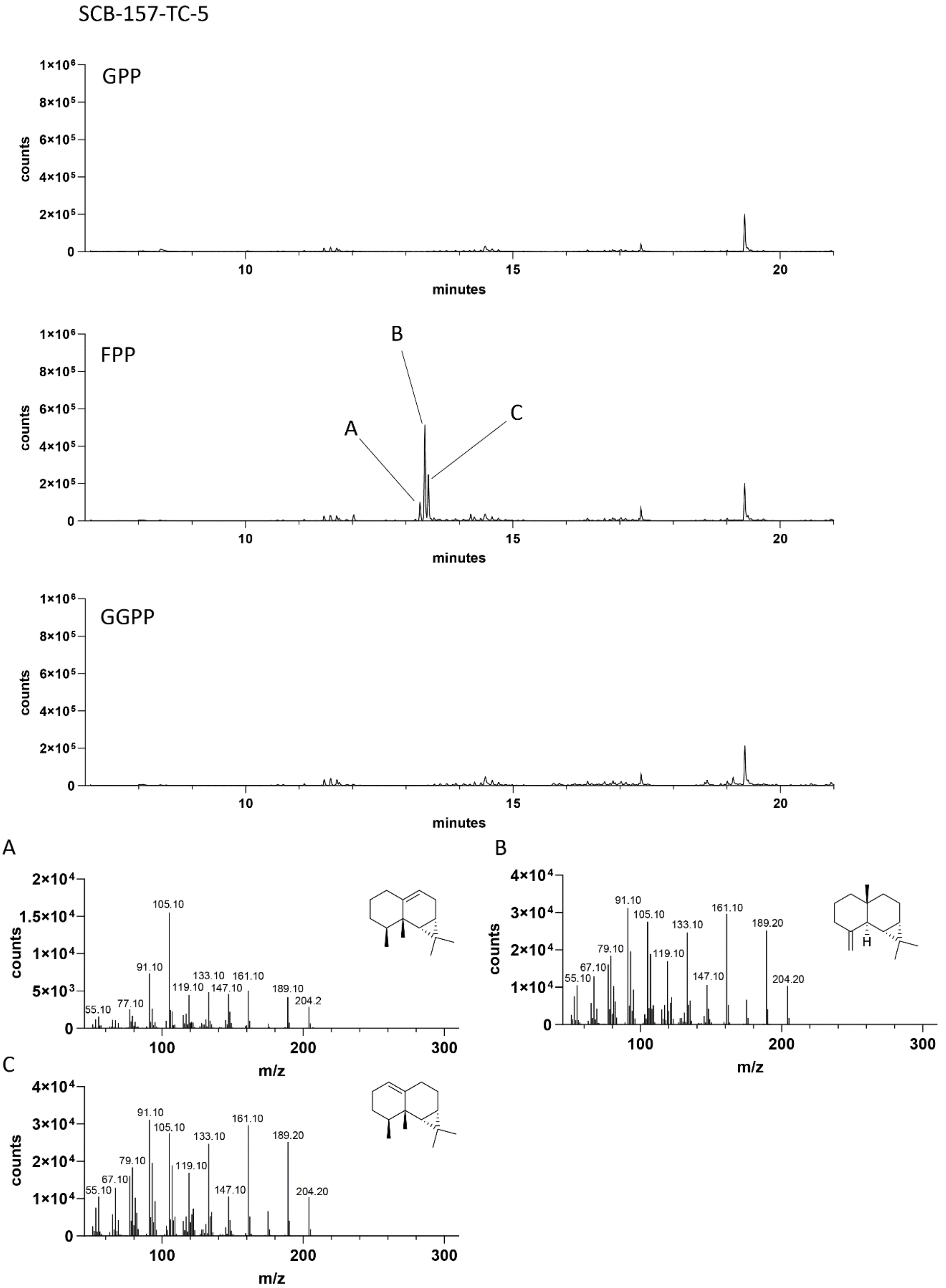

Figure S32: GCMS total ion chromatograms of SCB-157-TC-5 in vitro incubations with GPP (top), FPP (middle), and GGPP (bottom). Mass spectra of enzymatically produced terpenes are shown. The shown structures are best hits from the NIST database.

SCB-218-TC-1

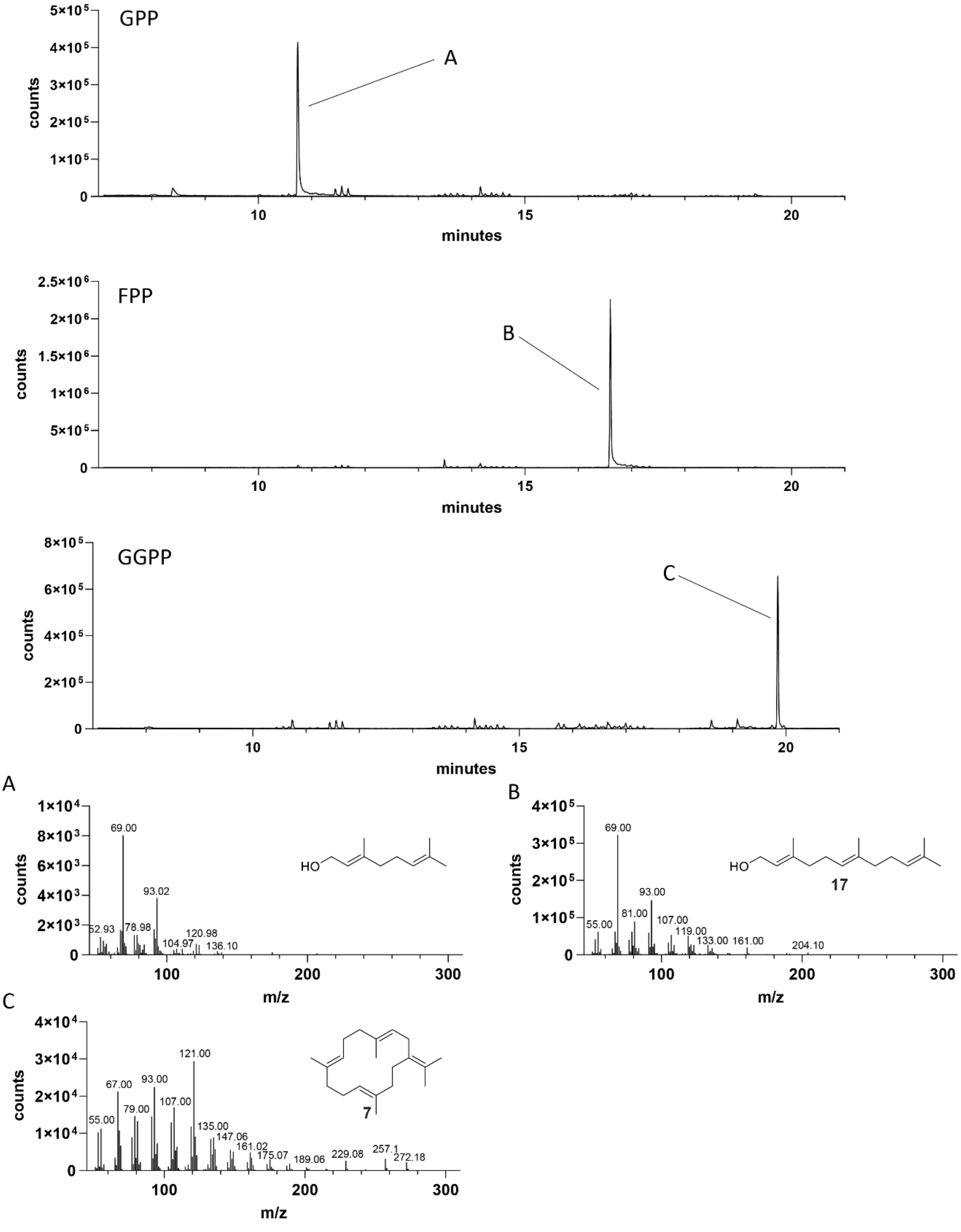

Figure S33: GCMS total ion chromatograms of SCB-218-TC-1 in vitro incubations with GPP (top), FPP (middle), and GGPP (bottom). Mass spectra of enzymatically produced terpenes are shown. The identity of **7** was proven using an authentic standard, the other shown structures are best hits from the NIST database.

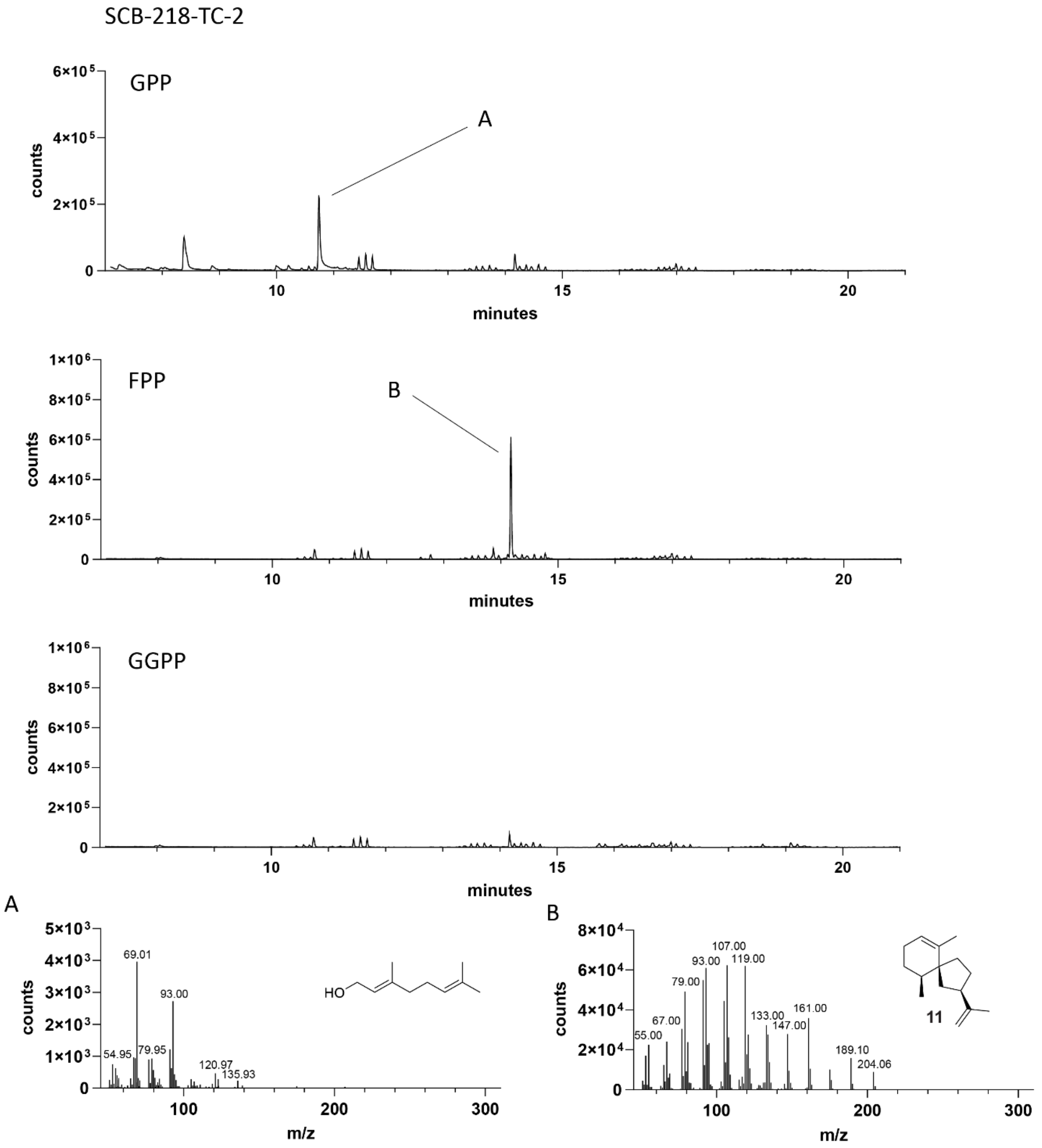

Figure S34: GCMS total ion chromatograms of SCB-218-TC-2 in vitro incubations with GPP (top), FPP (middle), and GGPP (bottom). Mass spectra of enzymatically produced terpenes are shown. The structure of **11** was elucidated by NMR spectroscopy of purified material from a large-scale in vitro assay.

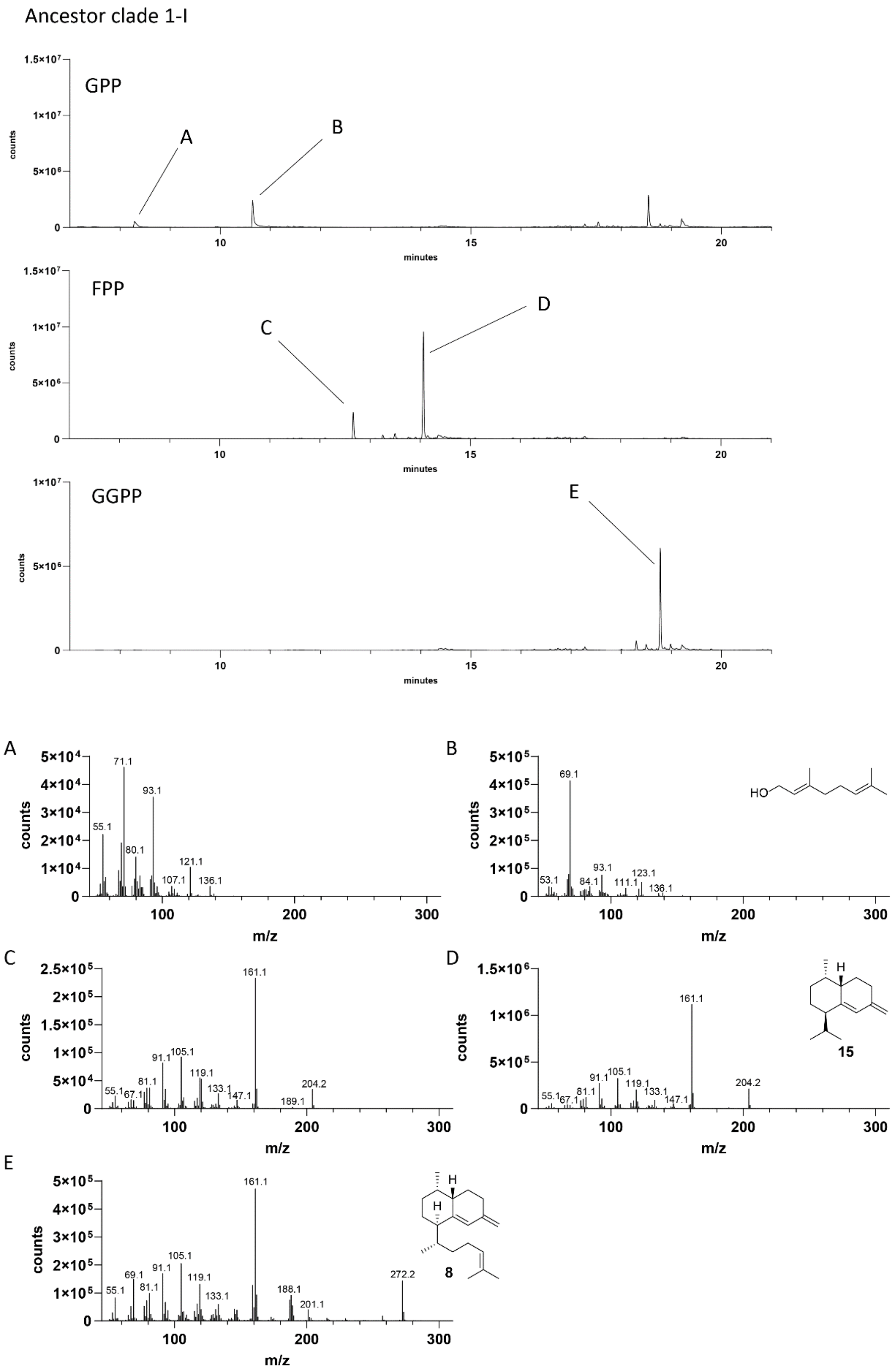

Figure S35: GCMS total ion chromatograms of in vitro incubations of the clade 1-I ancestor with GPP (top), FPP (middle), and GGPP (bottom). Mass spectra of enzymatically produced terpenes are shown. The identity of **8** and **15** was proven using an authentic standard, the other shown structures are best hits from the NIST database.

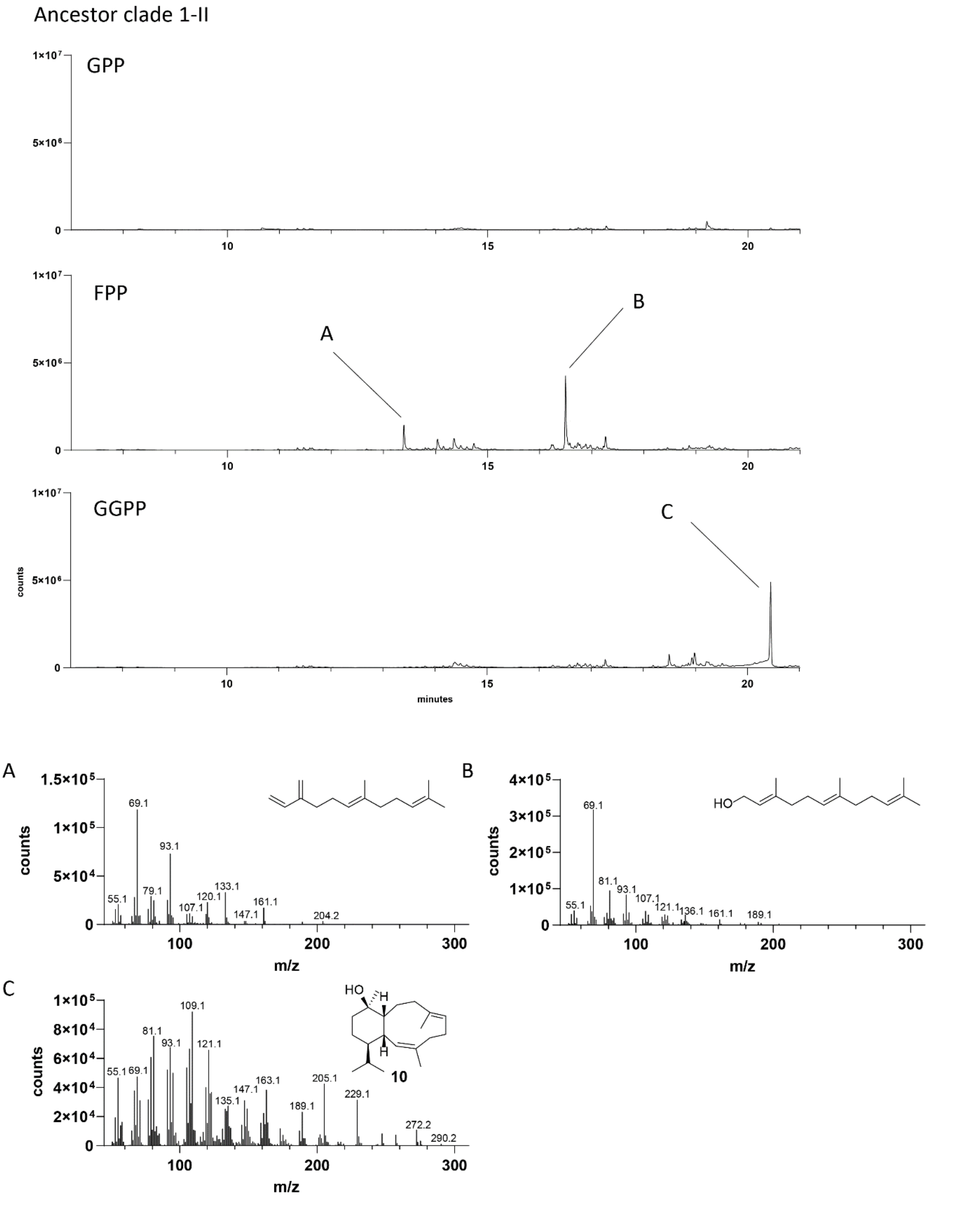

Figure S36: GCMS total ion chromatograms of in vitro incubations of the clade 1-II ancestor with GPP (top), FPP (middle), and GGPP (bottom). Mass spectra of enzymatically produced terpenes are shown. The identity of **10** was proven using an authentic standard, the other shown structures are best hits from the NIST database.

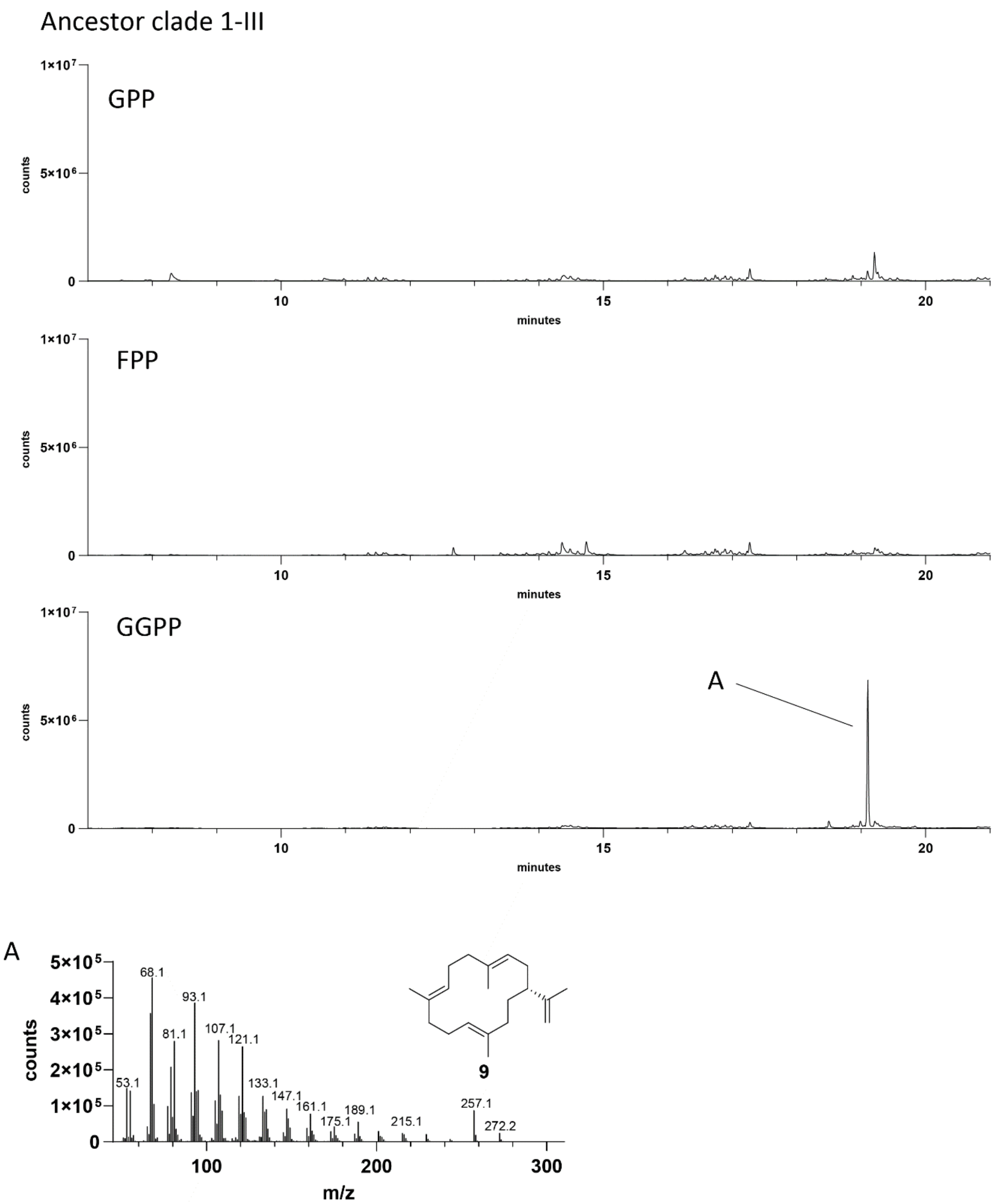

Figure S37: GCMS total ion chromatograms of in vitro incubations of the clade 1-III ancestor with GPP (top), FPP (middle), and GGPP (bottom). Mass spectra of enzymatically produced terpenes are shown. The identity of **9** was proven using an authentic standard.

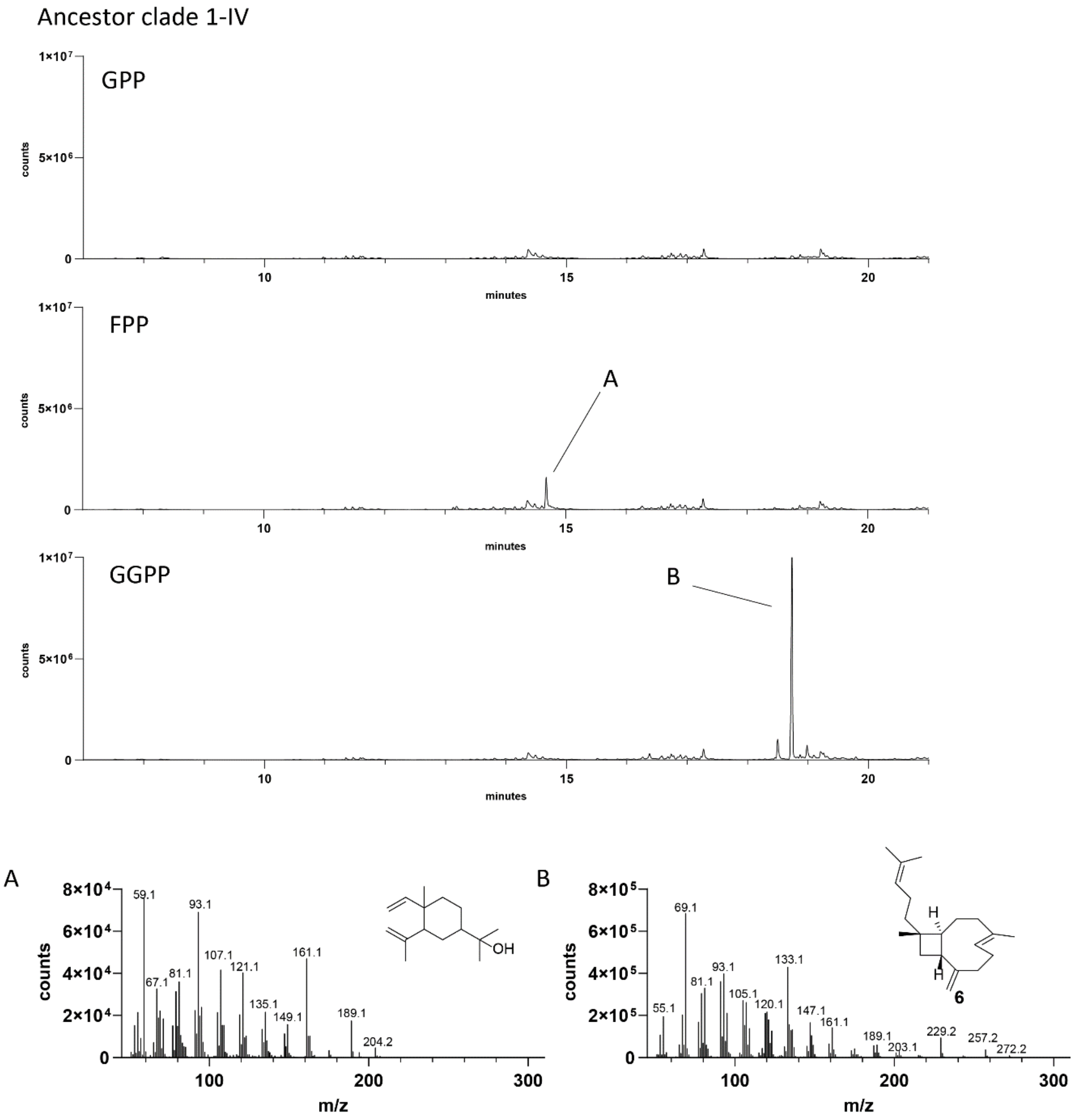

Figure S38: GCMS total ion chromatograms of in vitro incubations of the clade 1-IV ancestor with GPP (top), FPP (middle), and GGPP (bottom). Mass spectra of enzymatically produced terpenes are shown. The identity of **6** was proven using an authentic standard, the other shown structures are best hits from the NIST database.

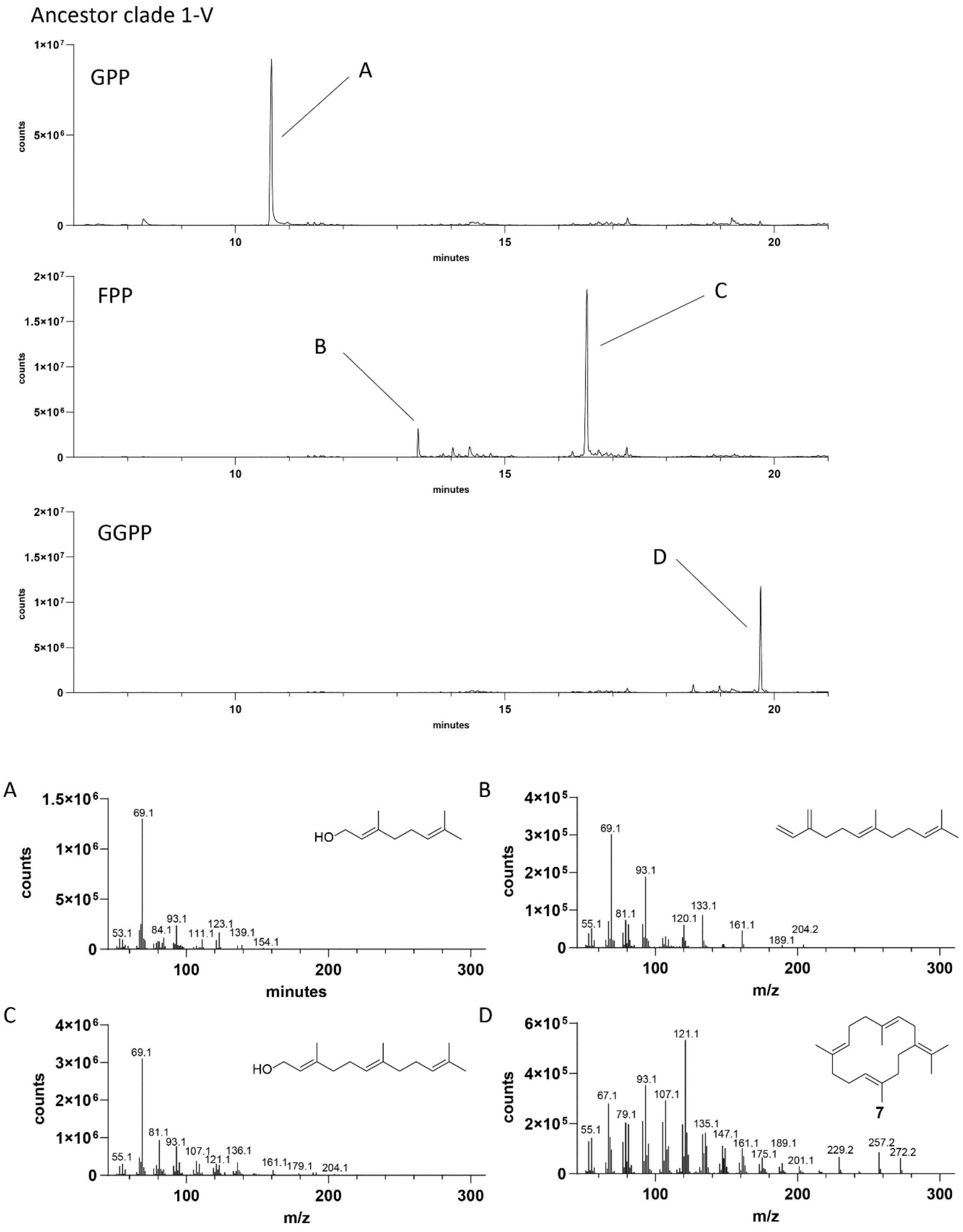

Figure S39: GCMS total ion chromatograms of in vitro incubations of the clade 1-V ancestor with GPP (top), FPP (middle), and GGPP (bottom). Mass spectra of enzymatically produced terpenes are shown. The identity of **7** was proven using an authentic standard, the other shown structures are best hits from the NIST database.

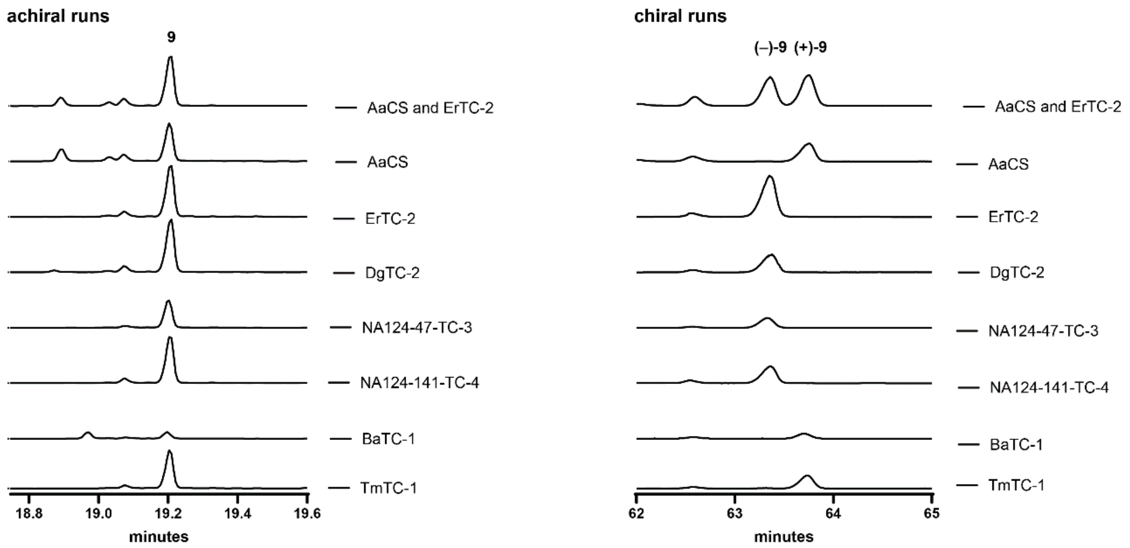

Figure S40: Determination of the absolute configuration of enzymatically obtained **9**. Left: Runs on achiral stationary phase; right: the same samples analyzed on a homochiral stationary phase. The absolute configuration of cembrene A produced by ErTC-2 and AaCS was elucidated previously as (-)-**9** and (+)-**9**, respectively.*(14, 29)*

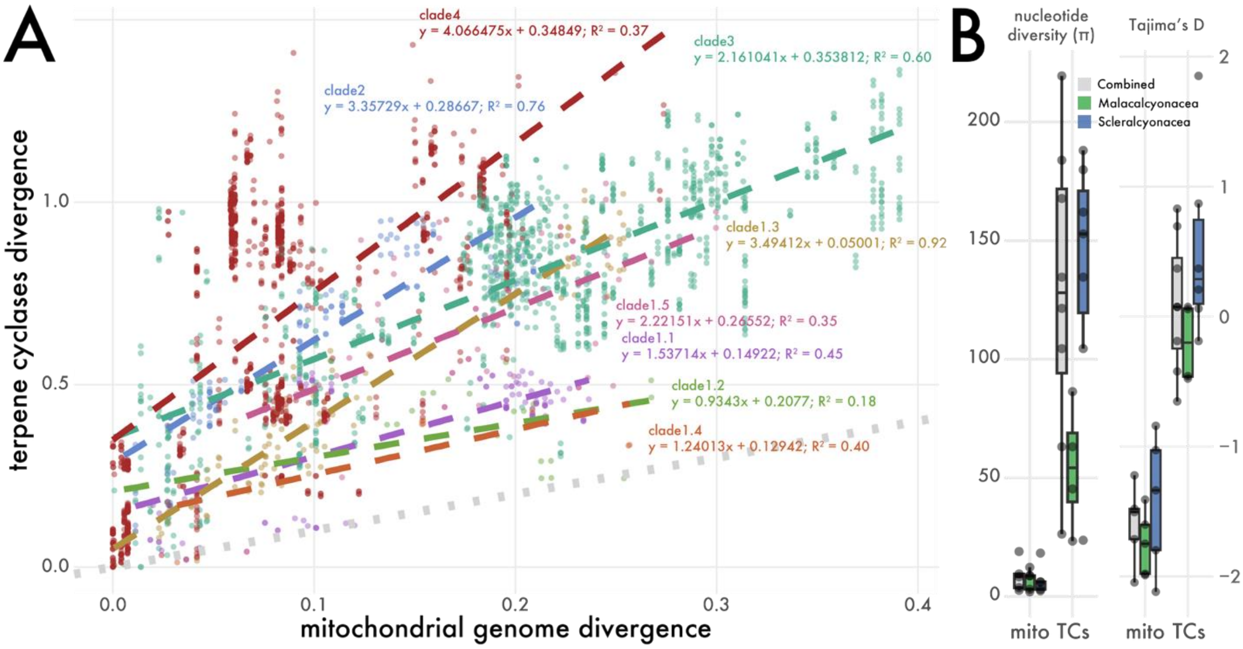

Figure S41 Evolutionary divergence and population genetic patterns of octocoral terpene cyclase (TC) genes. **A)** Pairwise nucleotide divergence of TC genes relative to mitochondrial genomes across octocoral taxa. Points are colored by TC clade (clades 1–4), with clade 1 subdivided into functionally characterized subclades (1-I to 1-V). Dashed lines show linear regressions for each clade; slope and adjusted R^2 values indicate relative evolutionary rates. The grey dashed line (slope = 1.0) reflects the expected divergence based on mitochondrial phylogeny. **B)** Nucleotide diversity (π) and Tajima's D for TC genes (clades 1–4 and subclades 1-I to 1-V) and mitochondrial genes, shown for all taxa and separated by order. Mitochondrial metrics provide a neutral baseline for comparison.

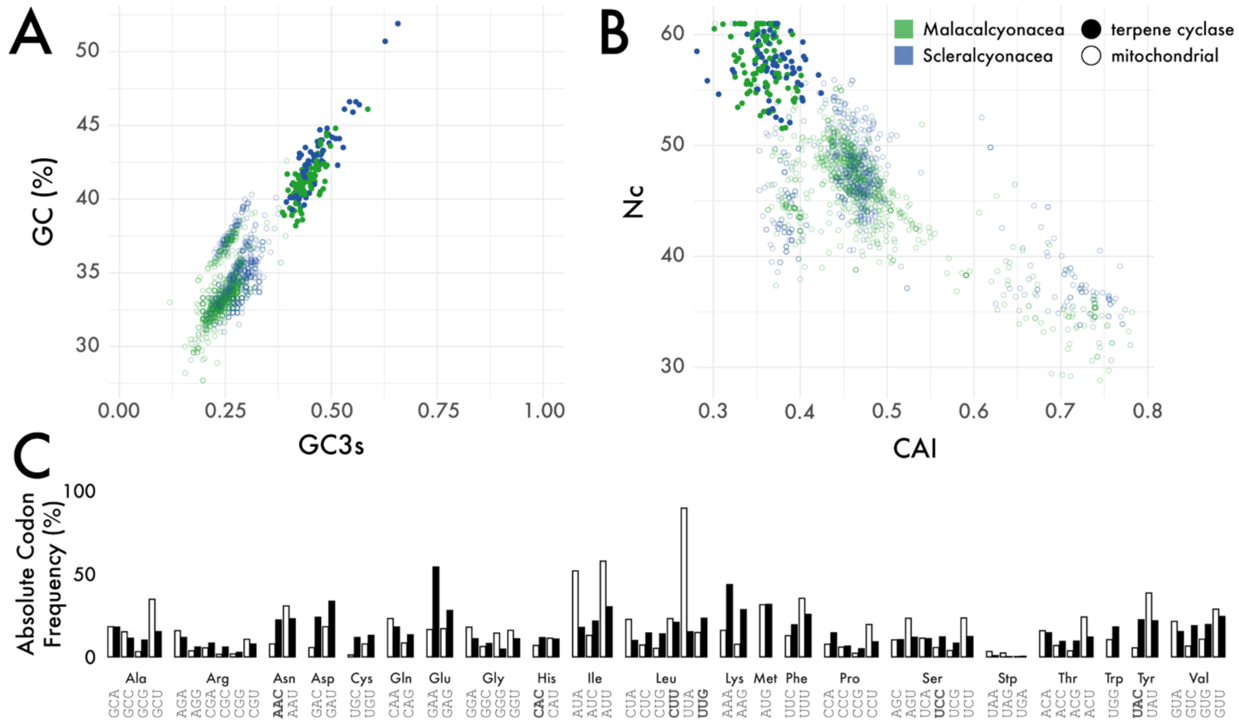

Figure S42: Codon composition and codon bias metrics in octocoral terpene cyclase (TC) genes. **A)** Neutrality plot showing the relationship between third-position GC content (GC_3) and overall GC content. Points are colored by taxonomic order (Malacalcyonacea in green, Scleralcyonacea in blue). TC genes exhibit a strong positive linear relationship between GC_3 and overall GC content, consistent with mutational bias influencing codon composition. However, these genes are systematically elevated in both overall GC and GC_3 relative to typical mitochondrial values, suggesting gene-specific differences in mutational pressures or base composition constraints. **B)** Relationship between effective codon number (Nc) and codon adaptation index (CAI). Lower Nc values correspond to stronger codon bias, while higher CAI values indicate increased codon usage bias. TC genes cluster in the high Nc, low CAI region, consistent with weak codon usage bias and relaxed synonymous site constraints. This pattern is expected for genes evolving under lineage-specific mutational biases or with codon usage patterns not strongly shaped by translational selection. **C)** Absolute codon frequencies for TC and mitochondrial genes, shown for all codons and grouped by encoded amino acid. Bars are colored by gene group (TC or mitochondrial). Mitochondrial genes serve as a comparison, highlighting differences in synonymous codon usage and overall codon composition between gene groups.

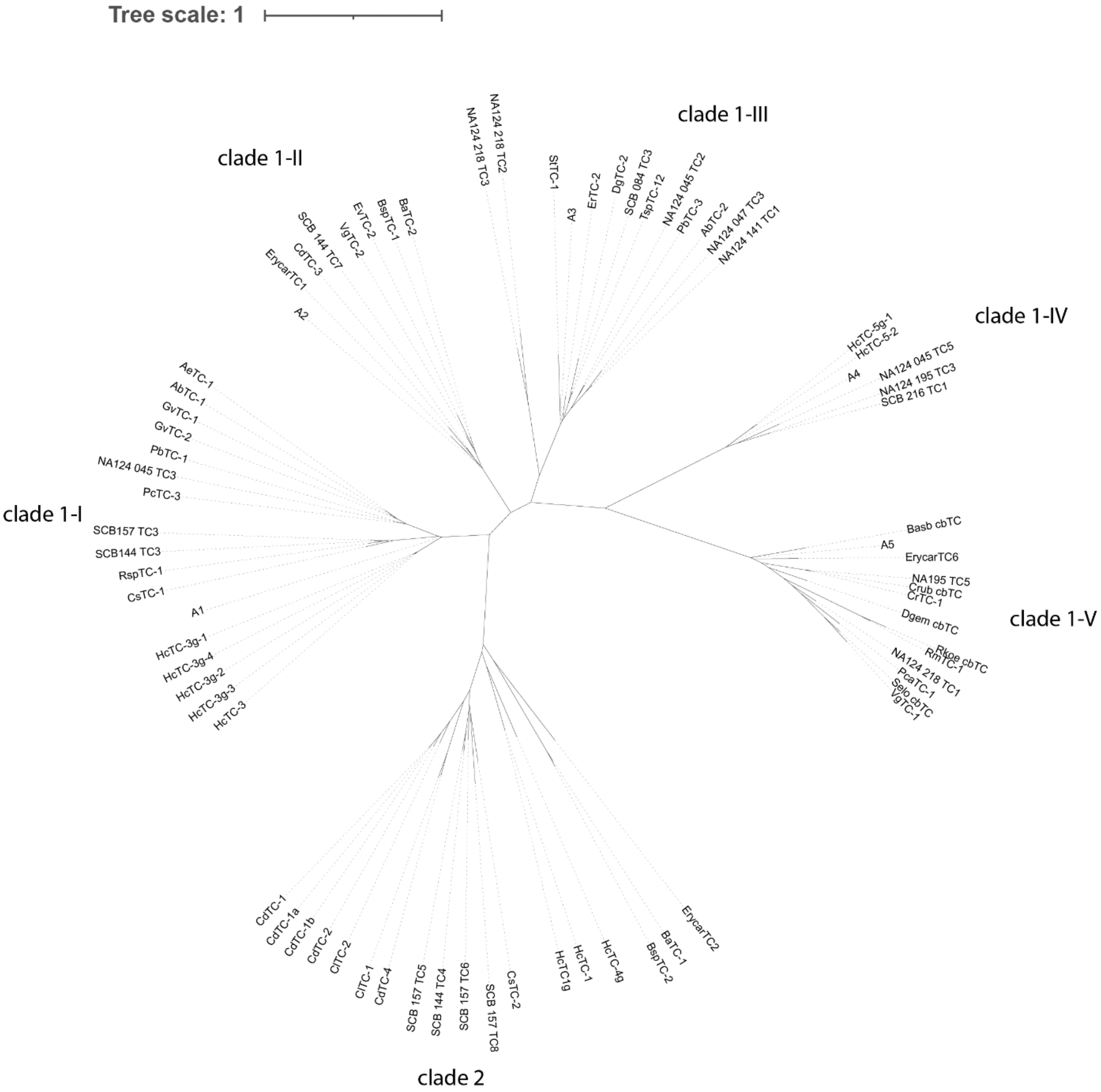

Figure S43: Partial tree including clades 1 and 2 to calculate ancestral sequences.

NMR spectra

11

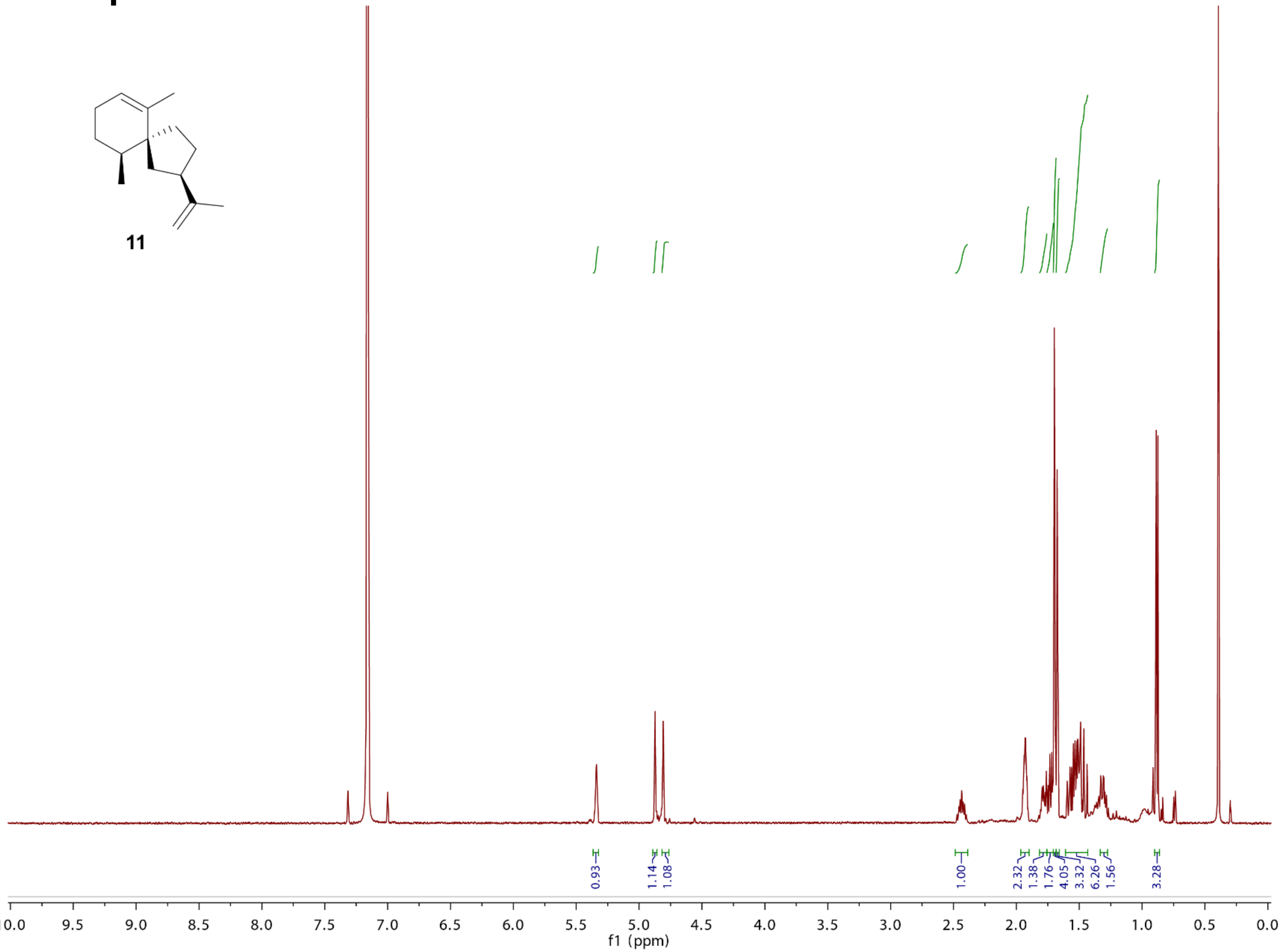

^{1}H-NMR spectrum (500 MHz) of **11** in C_6D_6.

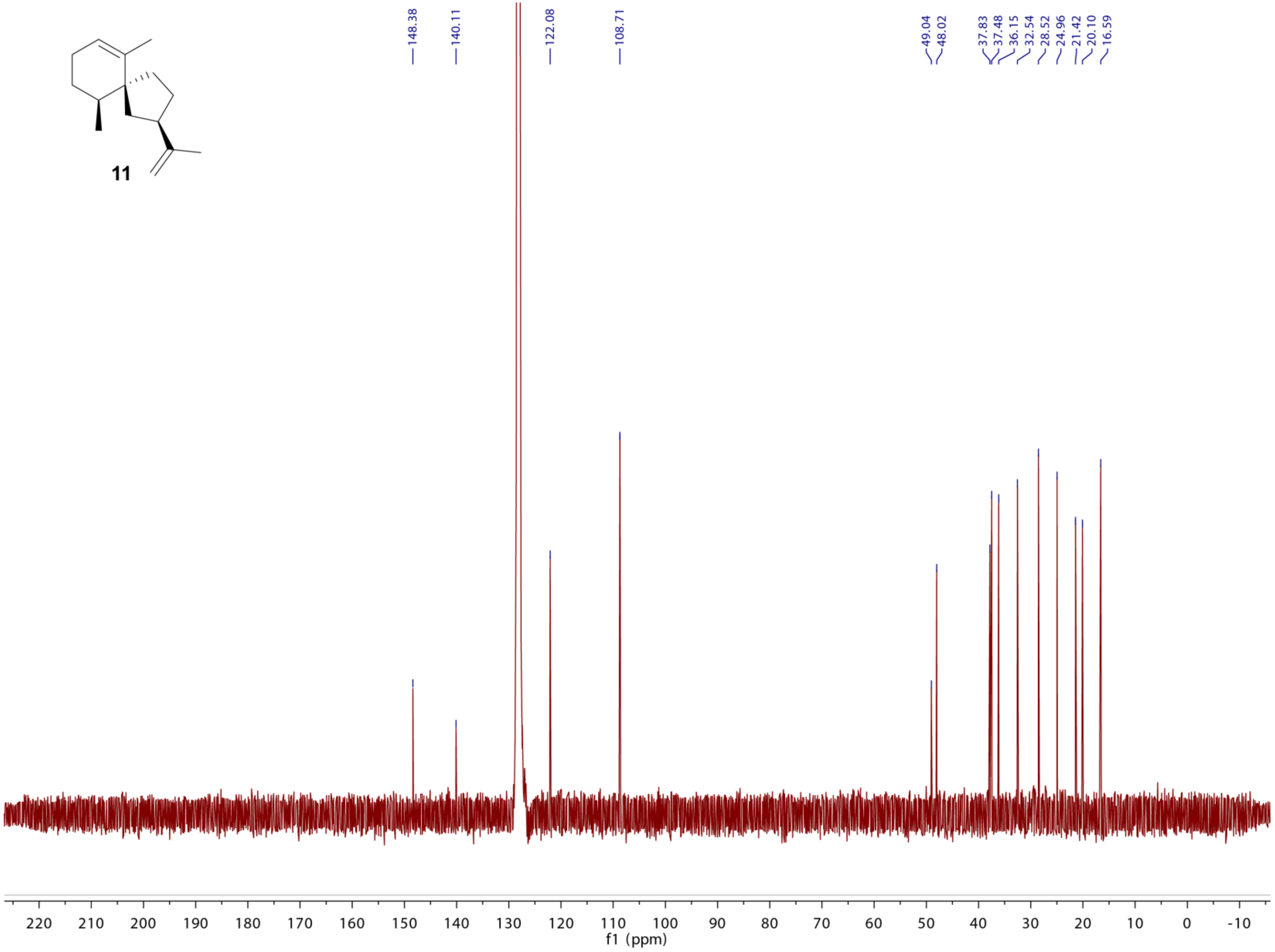

^{13}C-NMR spectrum (125 MHz) of **11** in C_6D_6.

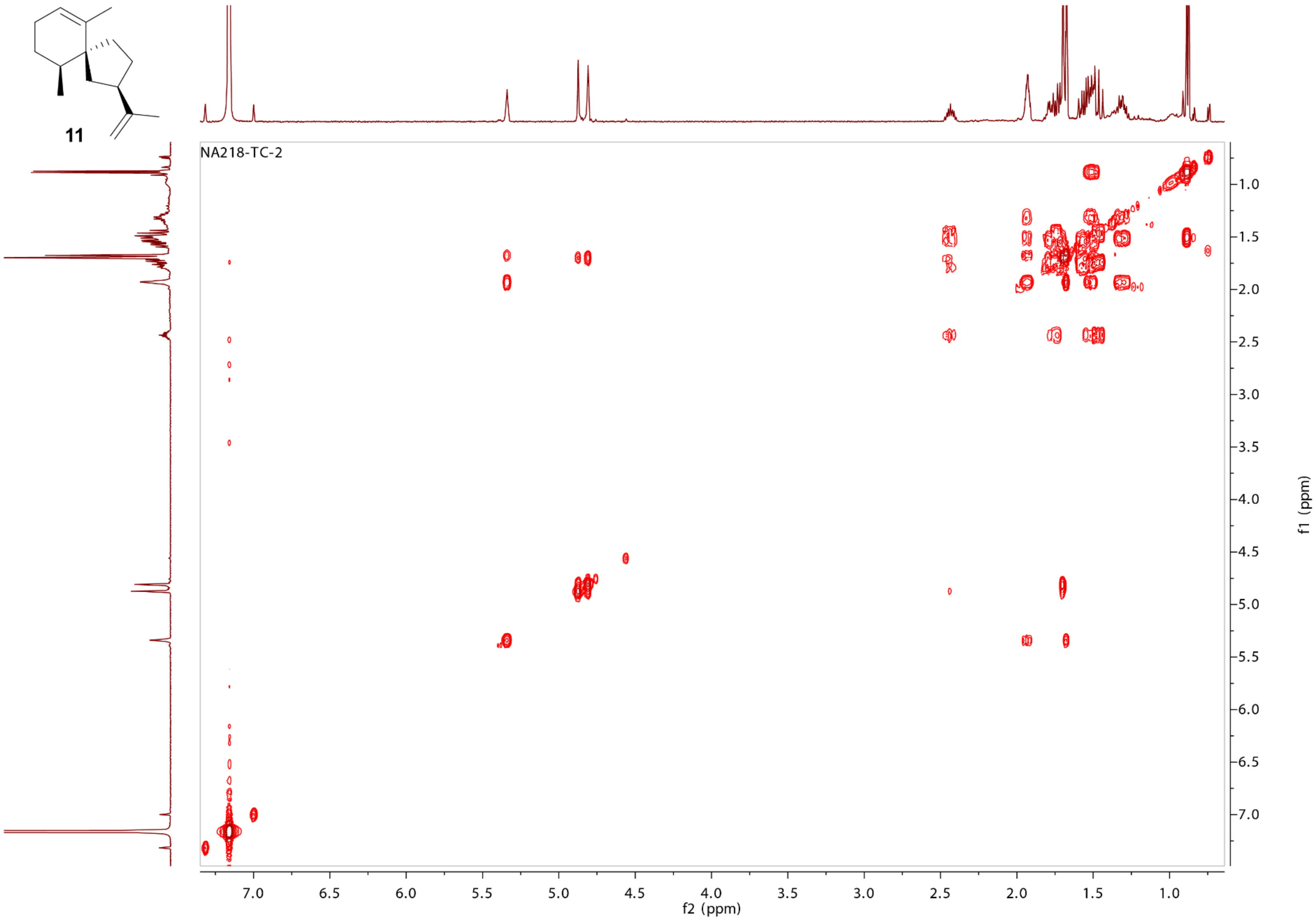

$^{1}H,^{1}H$-COSY spectrum (500 MHz) of **11** in C_6D_6.

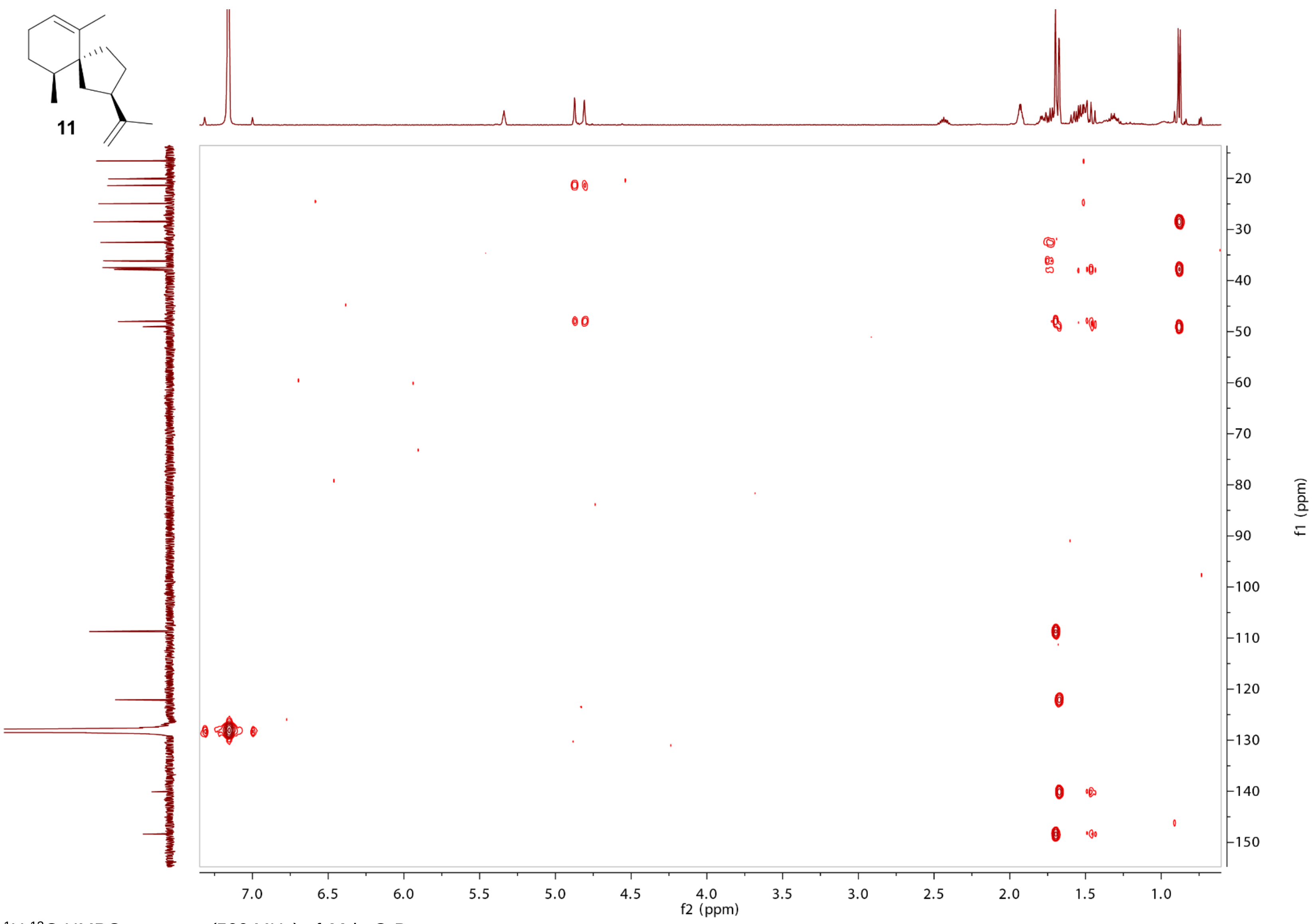

^{1}H,^{13}C-HMBC spectrum (500 MHz) of **11** in C_6D_6.

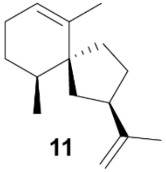

11

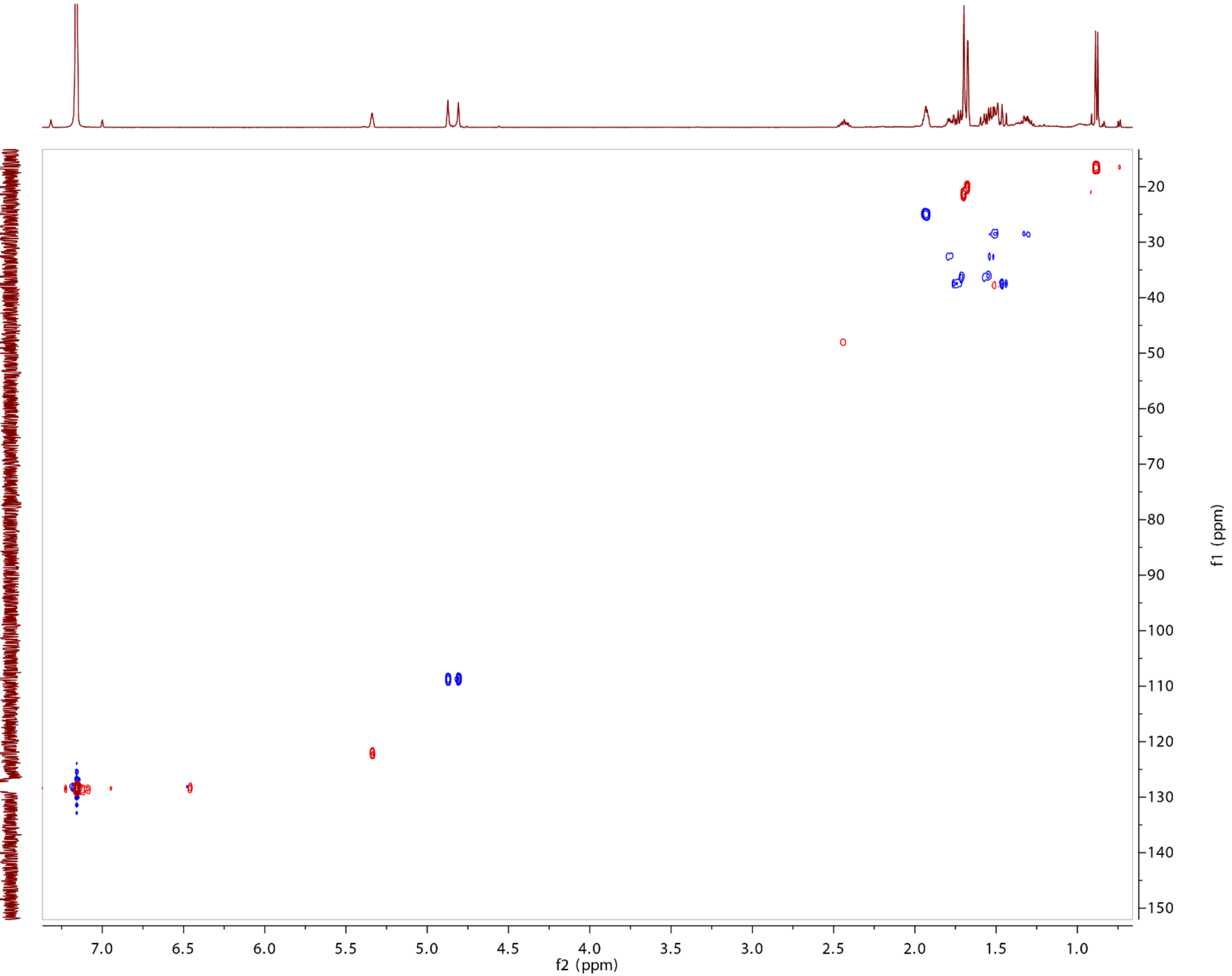

$^{1}H,^{13}C$-HSQC spectrum (500 MHz) of **11** in C_6D_6.

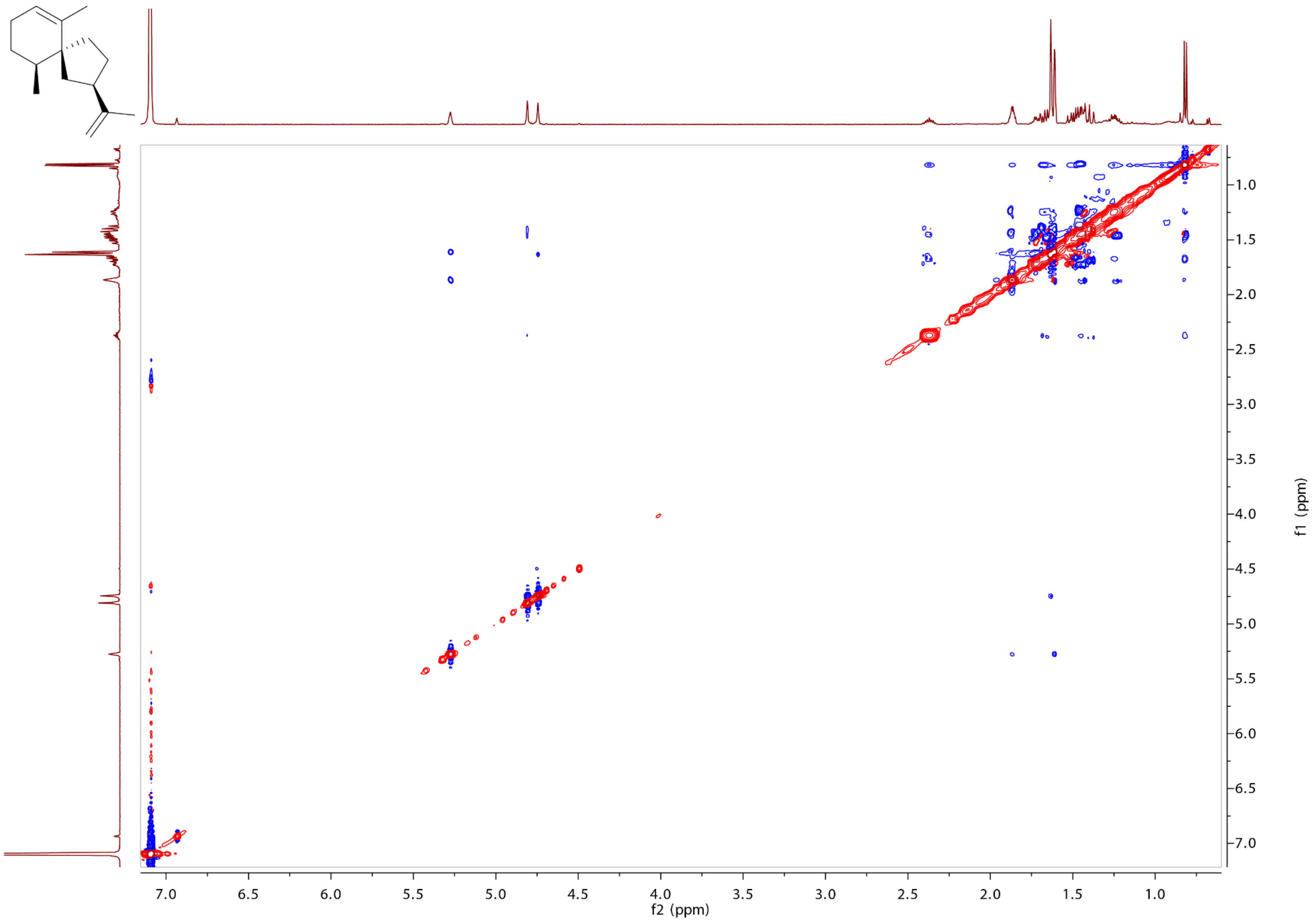

^{1}H,^{1}H-NOESY spectrum (500 MHz) of **11** in C_6D_6.

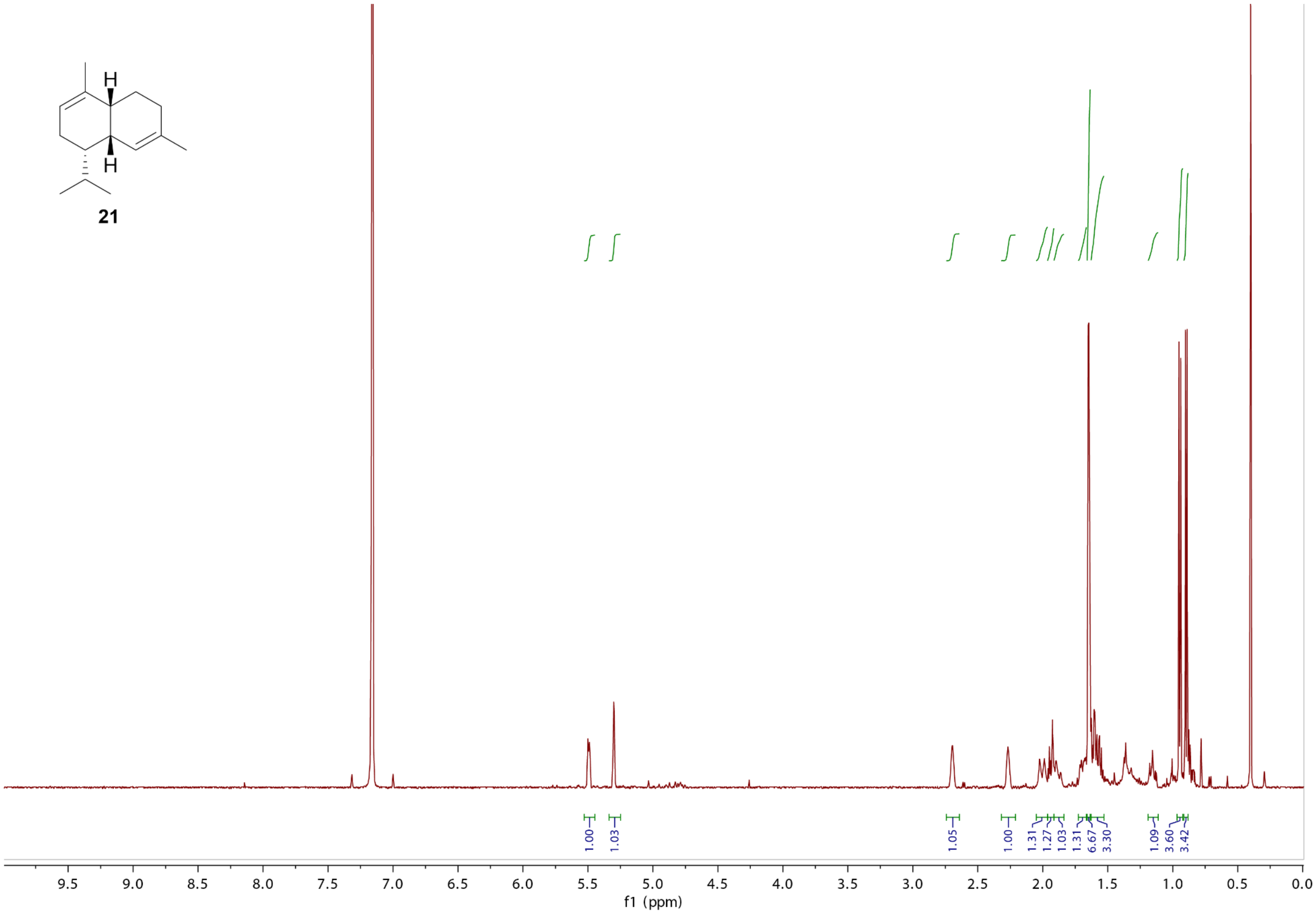

21

^{13}C-NMR spectrum (125 MHz) of **21** in C_6D_6.

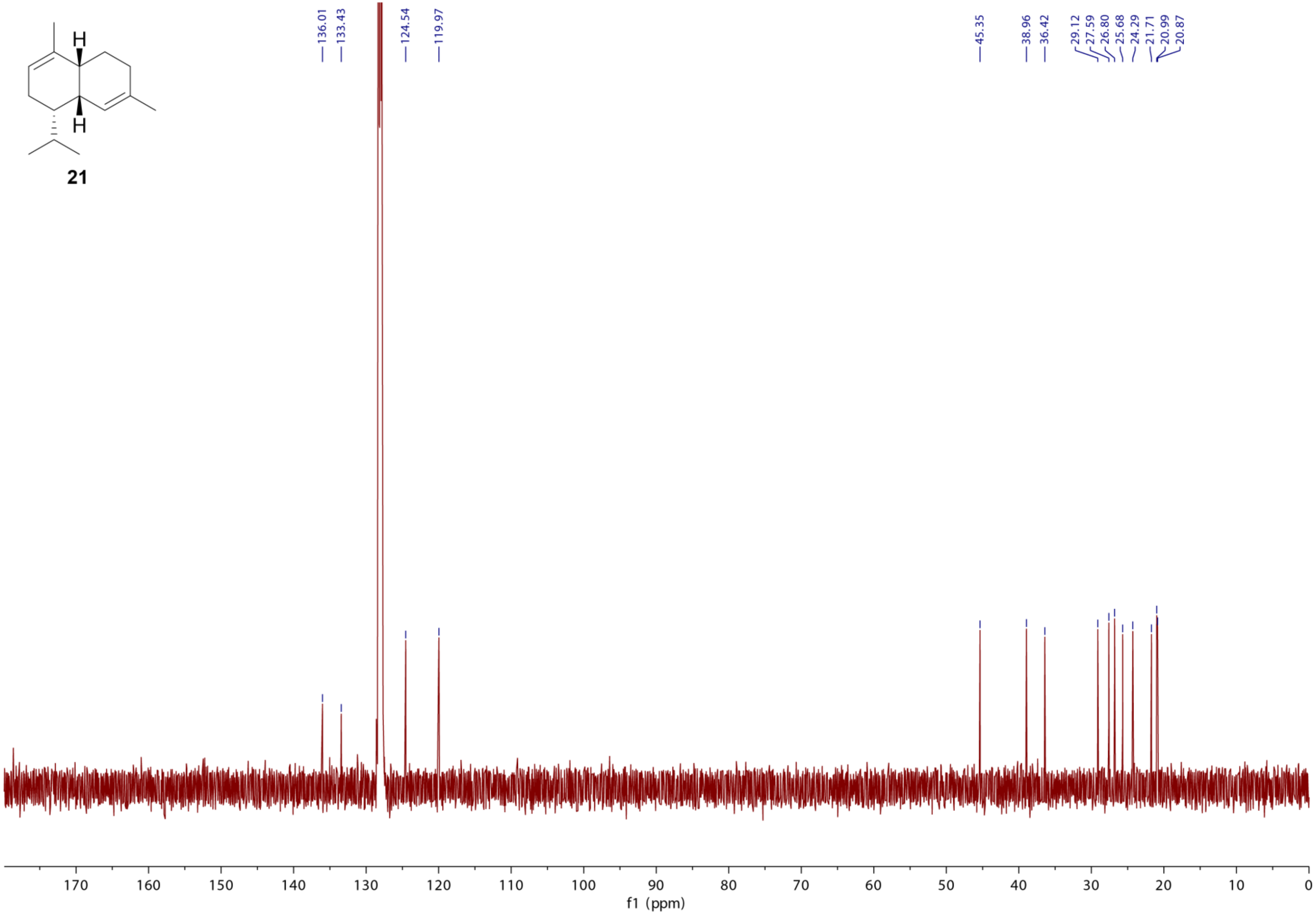

^{13}C-NMR spectrum (125 MHz) of **21** in C_6D_6.

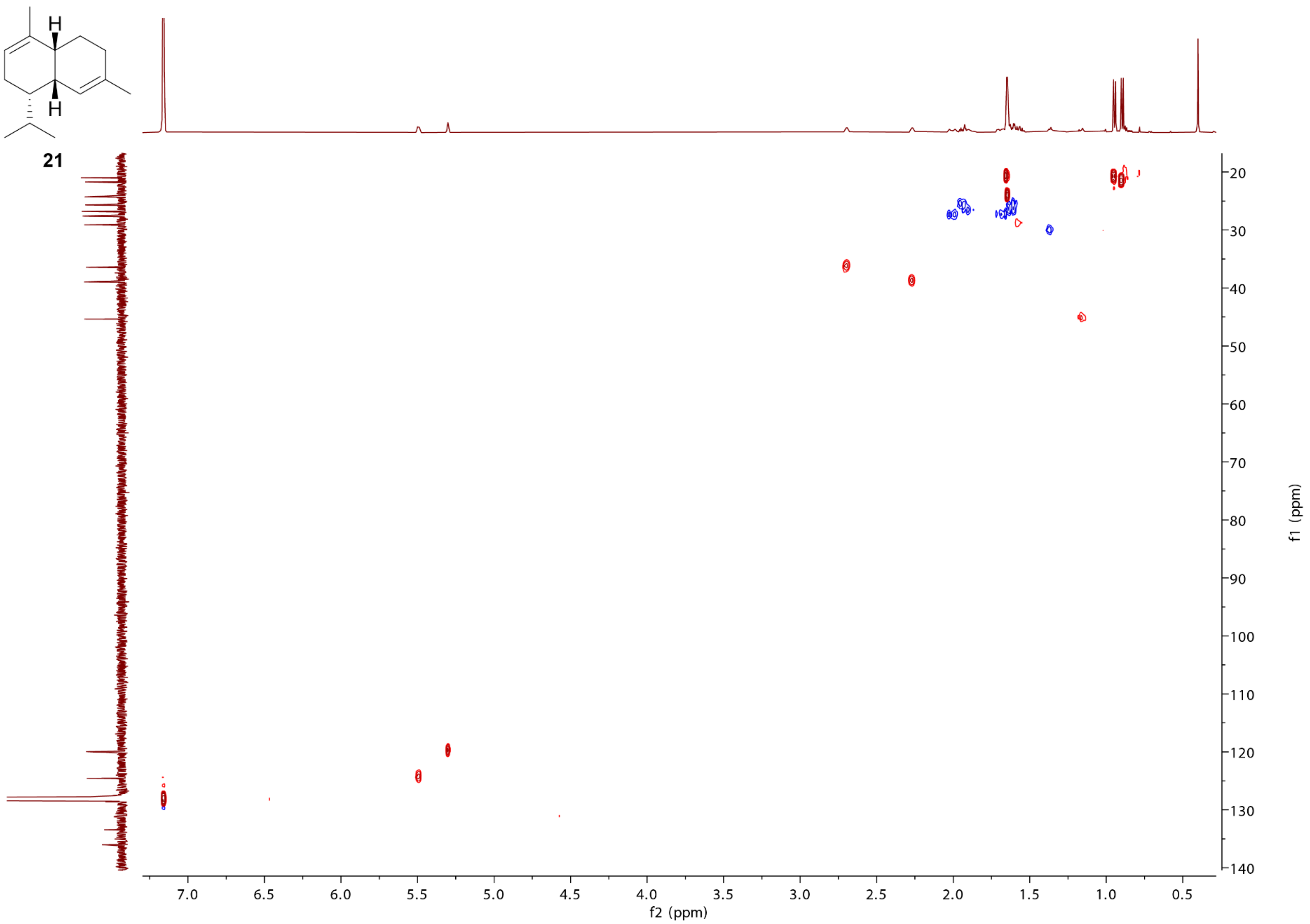

^{1}H,^{13}C-HSQC spectrum (500 MHz) of **21** in C_6D_6.

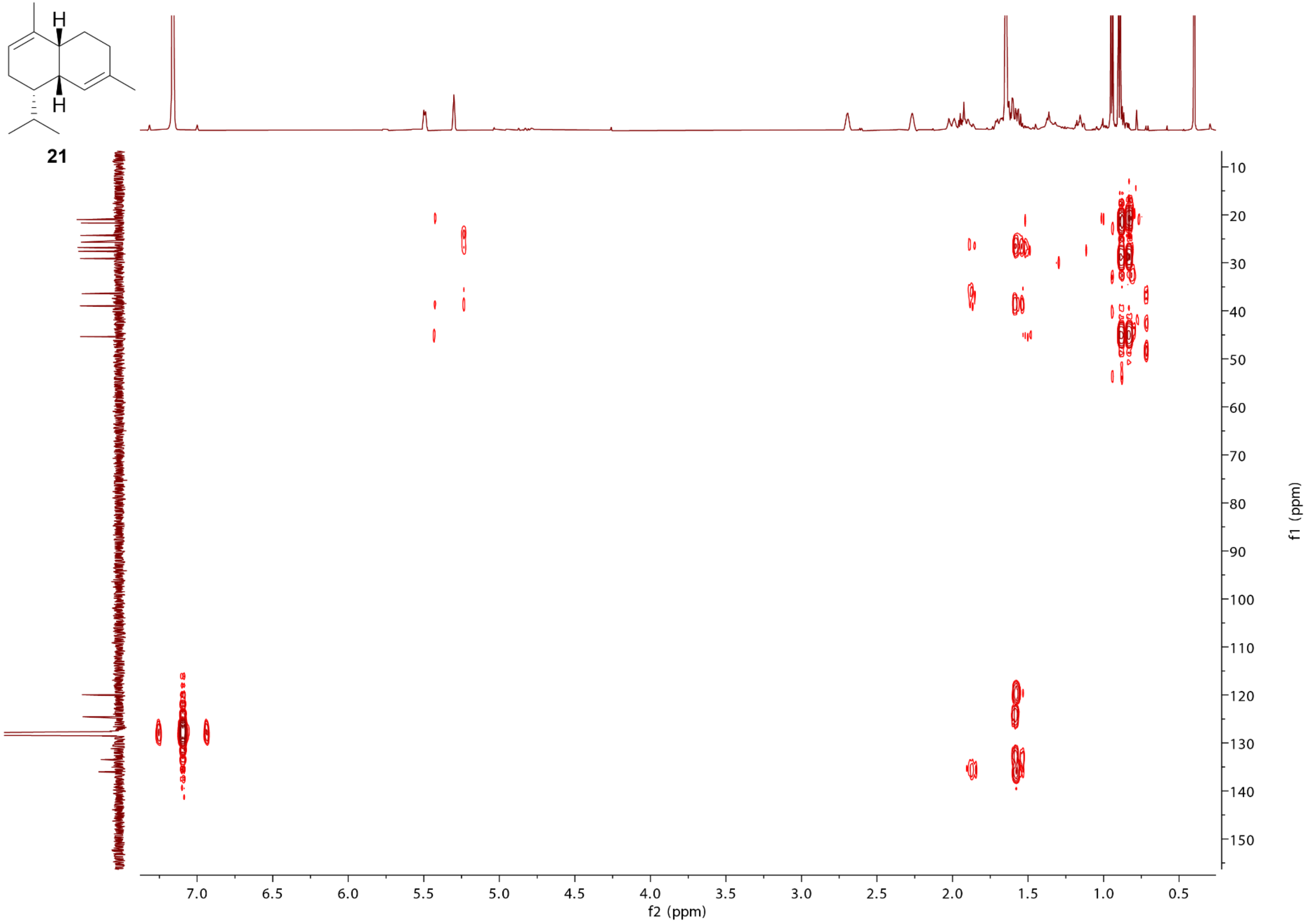

^{1}H,^{13}C-HMBC spectrum (500 MHz) of **21** in C_6D_6.

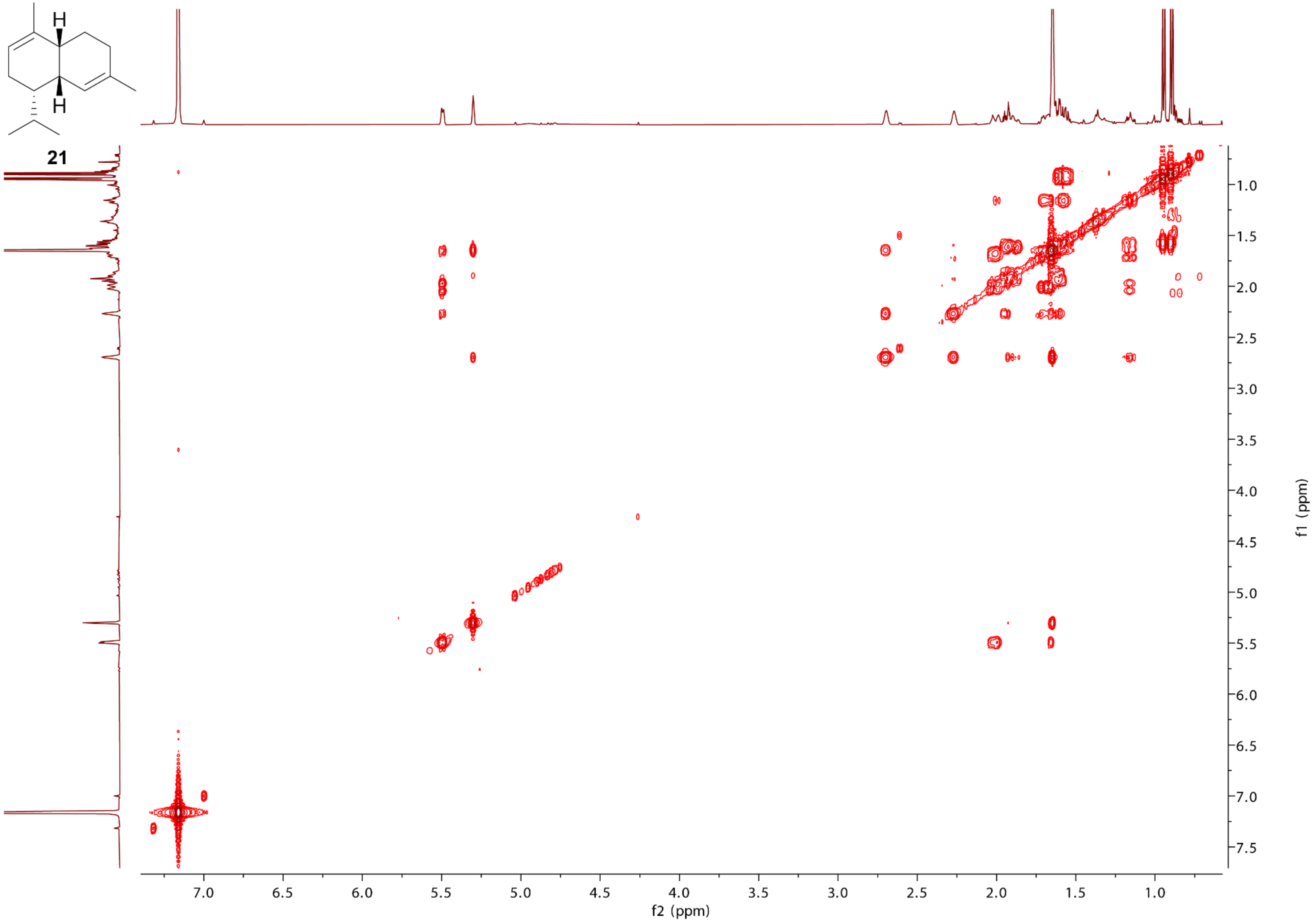

$^{1}H,^{1}H$-COSY spectrum (500 MHz) of **21** in C_6D_6.

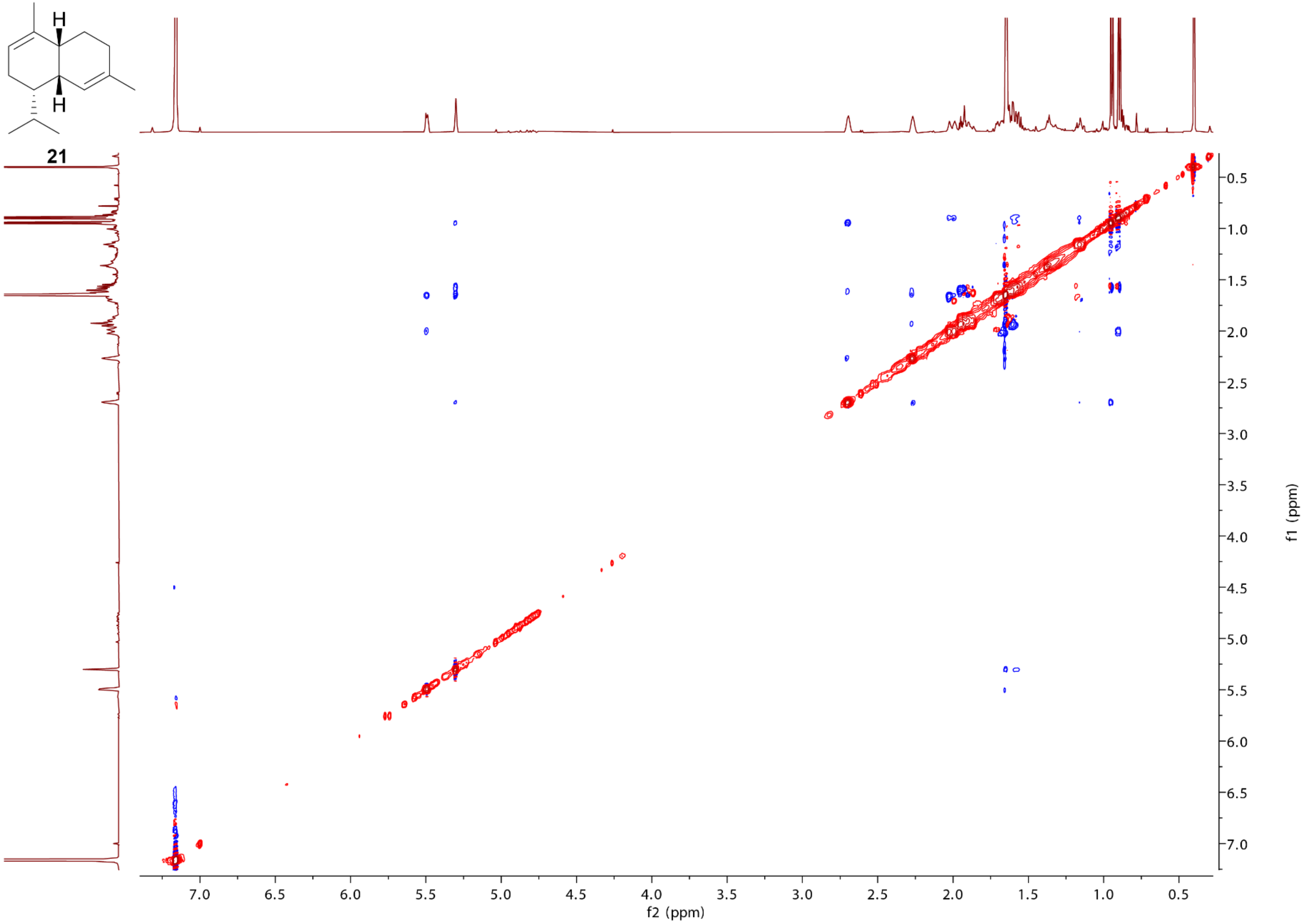

^{1}H,^{1}H-NOESY spectrum (500 MHz) of **21** in C_6D_6.

Supplementary References

1. A. B. Woodside, Z. Huang, C. D. Poulter, Trisammonium geranyl diphosphate. *Org. Synth.* **66**, 211 (1988).

2. V. J. Davisson, A. B. Woodside, C. D. Poulter, "Synthesis of allylic and homoallylic isoprenoid pyrophosphates" in *Methods in Enzymology*, Steroids and Isoprenoids, Part A., (Academic Press, 1985), pp. 130–144.

3. F. M. Bayer, M. Grasshoff, J. Verseveldt, Eds., *Illustrated trilingual glossary of morphological and anatomical terms applied to Octocorallia* (Brill, 2023).

4. C. S. McFadden, *et al.*, Limitations of mitochondrial gene barcoding in Octocorallia. *Mol. Ecol. Resour.*. **11**, 19–31 (2011).

5. A. Larsson, AliView: a fast and lightweight alignment viewer and editor for large datasets. *Bioinformatics* **30**, 3276–3278 (2014).

6. N. V. Patin, V. Kunin, U. Lidström, M. N. Ashby, Effects of OTU Clustering and PCR Artifacts on Microbial Diversity Estimates. *Microb. Ecol.* **65**, 709–719 (2013).

7. B. Bushnell, BBMap: A Fast, Accurate, Splice-Aware Aligner. (2014).

8. Y. Peng, H. C. M. Leung, S. M. Yiu, F. Y. L. Chin, IDBA-UD: a de novo assembler for single-cell and metagenomic sequencing data with highly uneven depth. *Bioinformatics* **28**, 1420–1428 (2012).

9. B. Langmead, S. L. Salzberg, Fast gapped-read alignment with Bowtie 2. *Nat. Methods* **9**, 357–359 (2012).

10. B. Buchfink, K. Reuter, H.-G. Drost, Sensitive protein alignments at tree-of-life scale using DIAMOND. *Nat. Methods* **18**, 366–368 (2021).

11. V. Muthye, C. D. Mackereth, J. B. Stewart, D. V. Lavrov, Large dataset of octocoral mitochondrial genomes provides new insights into *mt-mutS* evolution and function. *DNA Repair* **110**, 103273 (2022).

12. F. Sievers, D. G. Higgins, Clustal Omega. *Curr. Prot. Bioinf.* **48**, 3.13.1-3.13.16 (2014).

13. A. M. Kozlov, D. Darriba, T. Flouri, B. Morel, A. Stamatakis, RAxML-NG: a fast, scalable and user-friendly tool for maximum likelihood phylogenetic inference. *Bioinformatics* **35**, 4453–4455 (2019).

14. I. Burkhardt, T. de Rond, P. Y.-T. Chen, B. S. Moore, Ancient plant-like terpene biosynthesis in corals. *Nat Chem Biol* **18**, 664–669 (2022).

15. J. Mistry, R. D. Finn, S. R. Eddy, A. Bateman, M. Punta, Challenges in homology search: HMMER3 and convergent evolution of coiled-coil regions. *Nucleic Acids Res.* **41**, e121 (2013).

16. B. Q. Minh, *et al.*, IQ-TREE 2: New Models and Efficient Methods for Phylogenetic Inference in the Genomic Era. *Mol. Biol. Evol.* **37**, 1530–1534 (2020).

17. S. Kalyaanamoorthy, B. Q. Minh, T. K. F. Wong, A. von Haeseler, L. S. Jermiin, ModelFinder: fast model selection for accurate phylogenetic estimates. *Nat. Methods* **14**, 587–589 (2017).

18. I. Letunic, P. Bork, Interactive Tree of Life (iTOL) v6: recent updates to the phylogenetic tree display and annotation tool. *Nucleic Acids Res.* **52**, W78–W82 (2024).

19. B. Pfeifer, U. Wittelsbürger, S. E. Ramos-Onsins, M. J. Lercher, PopGenome: An Efficient Swiss Army Knife for Population Genomic Analyses in R. *Mol. Biol. Evol.* **31**, 1929–1936 (2014).

20. D. Charif, J. R. Lobry, "SeqinR 1.0-2: A Contributed Package to the R Project for Statistical Computing Devoted to Biological Sequences Retrieval and Analysis" in *Structural Approaches to Sequence Evolution: Molecules, Networks, Populations*, U. Bastolla, M. Porto, H. E. Roman, M. Vendruscolo, Eds. (Springer, 2007), pp. 207–232.

21. E. Paradis, J. Claude, K. Strimmer, APE: Analyses of Phylogenetics and Evolution in R language. *Bioinformatics* **20**, 289–290 (2004).

22. S. Kryazhimskiy, J. B. Plotkin, The Population Genetics of dN/dS. *PLOS Genet.* **4**, e1000304 (2008).

23. R. Khandia, *et al.*, Analysis of Nipah Virus Codon Usage and Adaptation to Hosts. *Front. Microbiol.* **10** (2019).

24. Correspondence Analysis of Codon Usage. Available at: https://codonw.sourceforge.net/ [Accessed 10 July 2025].

25. B. Plese, *et al.*, Mitochondrial evolution in the Demospongiae (Porifera): Phylogeny, divergence time, and genome biology. *Mol. Phylogenet. Evol.* **155**, 107011 (2021).

26. N. Sueoka, Intrastrand parity rules of DNA base composition and usage biases of synonymous codons. *J. Mol. Evol.* **40**, 318–325 (1995).

27. G. Blay, *et al.*, Synthesis of Spirovetivane Sesquiterpenes from Santonin. Synthesis of (+)-Anhydro-β-rotunol and All Diastereomers of 6,11-Spirovetivadiene. *J. Org. Chem.* **69**, 7294–7302 (2004).

28. P. Rabe, T. Schmitz, J. S. Dickschat, Mechanistic investigations on six bacterial terpene cyclases. *Beilstein J. Org. Chem.* **12**, 1839–1850 (2016).

29. J. Rinkel, L. Lauterbach, P. Rabe, J. S. Dickschat, Two Diterpene Synthases for Spiroalbatene and Cembrene A from Allokutzneria albata. *Angew. Chem. Int. Ed.* **57**, 3238–3241 (2018).

30. M. Musil, *et al.*, FireProtASR: A Web Server for Fully Automated Ancestral Sequence Reconstruction. *Brief. Bioinform.* **22**, bbaa337 (2021).

31. L. P. Patiño Cano, *et al.*, Isolation and Antifouling Activity of Azulene Derivatives from the Antarctic Gorgonian Acanthogorgia laxa. *Chem. Biodivers.* **15**, e1700425 (2018).

32. E. Manzo, *et al.*, A new xenicane norditerpene from the Indian marine gorgonian Acanthogorgia turgida. *Nat. Prod. Res.* **23**, 1664–1670 (2009).

33. V. A. Stonik, T. N. Makar’eva, A. S. Dmitrenok, New diterpenoid of the xeniane series from the gorgonianParagorgia arborea. *Chem. Nat. Compd.* **26**, 103–104 (1990).

34. M. D’Ambrosio, A. Guerriero, F. Pietra, Arboxeniolide-1, a New, Naturally Occurring Xeniolide Diterpenoid from the Gorgonian Paragorgia arborea of the Crozet Is. (S. Indian Ocean). *Z. Naturforsch. C* **39**, 1180–1183 (1984).

35. S.-K. Wang, M.-J. Huang, C.-Y. Duh, Cytotoxic Constituents from the Formosan Soft Coral Clavularia inflata var. luzoniana. *J. Nat. Prod.* **69**, 1411–1416 (2006).

36. D. Banjoo, *et al.*, New Erythrolides from the Caribbean Gorgonian Octocoral Erythropodium caribaeorum. *J. Nat. Prod.* **65**, 314–318 (2002).

37. B. Cinel, *et al.*, Antimitotic Diterpenes from Erythropodium caribaeorum Test Pharmacophore Models for Microtubule Stabilization. *Org. Lett.* **2**, 257–260 (2000).

38. A. D. Rodríguez, O. M. Cóbar, The briarellins, new eunicellin-based diterpenoids from a Caribbean gorgonian, *Briareum asbestinum*. *Tetrahedron* **51**, 6869–6880 (1995).

39. A. D. Rodríguez, C. Ramírez, O. M. Cóbar, Briareins C−L, 10 New Briarane Diterpenoids from the Common Caribbean Gorgonian Briareum asbestinum. *J. Nat. Prod.* **59**, 15–22 (1996).

40. C.-C. Liaw, *et al.*, Frajunolides E−K, Briarane Diterpenes from Junceella fragilis. *J. Nat. Prod.* **71**, 1551–1556 (2008).

41. A. Guerriero, M. D’Ambrosio, F. Pietra, Verecynarmin A, a Novel Briarane Diterpenoid Isolated from Both the Mediterranean Nudibranch Mollusc Armina maculata and its Prey, the Pennatulacean Octocoral Veretillum cynomorium. *Helv. Chim. Acta* **70**, 984–991 (1987).

42. A. Guerriero, M. D’Ambrosio, F. Pietra, Isolation of the Cembranoid Preverecynarmin Alongside Some Briaranes, the Verecynarmins, from Both the Nudibranch Mollusc Armina maculata and the Octocoral Veretillum cynomorium of the East Pyrenean Mediterranean Sea. *Helv. Chim. Acta* **73**, 277–283 (1990).

43. S. J. Wratten, D. J. Faulkner, K. Hirotsu, J. Clardy, Stylatulide, a sea pen toxin. *J. Am. Chem. Soc.* **99**, 2824–2825 (1977).

44. A. Bahl, *et al.*, 2-Acetoxyverecynarmin C, a New Briarane COX Inhibitory Diterpenoid from *Pennatula aculeata*. *Nat. Prod. Commun.* **9**, 1934578X1400900820 (2014).

45. C.-Y. Duh, R.-S. Hou, Cytotoxic Cembranoids from the Soft Corals Sinularia gibberosa and Sarcophyton trocheliophorum. *J. Nat. Prod.* **59**, 595–598 (1996).

46. J. Bernstein, U. Shmeuli, E. Zadock, Y. Kashman, I. Néeman, Sarcophine, a new epoxy cembranolide from marine origin. *Tetrahedron* **30**, 2817–2824 (1974).

47. T. Wen, *et al.*, Sinulaflexiolides A−K, Cembrane-Type Diterpenoids from the Chinese Soft Coral Sinularia flexibilis. *J. Nat. Prod.* **71**, 1133–1140 (2008).

48. T. Kusumi, H. Uchida, M. O. Ishitsuka, H. Yamamoto, H. Kakisawa, Alcyonin, a New Cladiellane Diterpene from the Soft Coral Sinularia flexibilis. *Chem. Lett.* **17**, 1077–1078 (1988).

49. Y.-J. Tseng, A. F. Ahmed, C.-F. Dai, M. Y. Chiang, J.-H. Sheu, Sinulochmodins A−C, Three Novel Terpenoids from the Soft Coral Sinularia lochmodes. *Org. Lett.* **7**, 3813–3816 (2005).

50. N. P. Thao, *et al.*, Diterpenoids from the Soft Coral Sinularia maxima and Their Inhibitory Effects on Lipopolysaccharide-Stimulated Production of Pro-inflammatory Cytokines in Bone Marrow-Derived Dendritic Cells. *Chem. Pharm. Bull.* **60**, 1581–1589 (2012).

51. M. M. Radwan, *et al.*, Sinulodurins A and B, Antiproliferative and Anti-invasive Diterpenes from the Soft Coral Sinularia dura. *J. Nat. Prod.* **71**, 1468–1471 (2008).

52. G. J. Hooper, M. T. Davies-Coleman, New Metabolites from the South African soft coral *Capnella thyrsoidea*. *Tetrahedron* **51**, 9973–9984 (1995).

53. H. M. Hassan, *et al.*, Pachycladins A−E, Prostate Cancer Invasion and Migration Inhibitory Eunicellin-Based Diterpenoids from the Red Sea Soft Coral Cladiella pachyclados. *J. Nat. Prod.* **73**, 848–853 (2010).

54. A. Rudi, *et al.*, Antheliatin and Zahavins A and B, Three New Cytotoxic Xenicane Diterpenes from Two Soft Corals. *J. Nat. Prod.* **58**, 1581–1586 (1995).

55. C. Urda, *et al.*, Protoxenicins A and B, Cytotoxic Long-Chain Acylated Xenicanes from the Soft Coral Protodendron repens. *J. Nat. Prod.* **80**, 713–719 (2017).

56. A. Groweiss, Y. Kashman, Xeniculin, xeniaphyllenol and xeniaphyllenol oxide, new diterpenoids from the soft-coral xenia macrospiculata. *Tetrahedron Lett.* **19**, 2205–2208 (1978).

57. S. D. Rosa, *et al.*, A New Bioactive Eunicellin-Type Diterpene from the Gorgonian Eunicella cavolini. *Nat. Prod. Lett.* **7**, 259–265 (1995).

58. S. A. Look, W. Fenical, G. K. Matsumoto, J. Clardy, The pseudopterosins: a new class of antiinflammatory and analgesic diterpene pentosides from the marine sea whip Pseudopterogorgia elisabethae (Octocorallia). *J. Org. Chem.* **51**, 5140–5145 (1986).

59. A. D. Rodríguez, J.-G. Shi, S. D. Huang, Highly Oxygenated Pseudopterane and Cembranolide Diterpenes from the Caribbean Sea Feather Pseudopterogorgia bipinnata. *J. Nat. Prod.* **62**, 1228–1237 (1999).

60. M. Gutiérrez, *et al.*, Leptolide, a New Furanocembranolide Diterpene from Leptogorgia alba. *J. Nat. Prod.* **68**, 614–616 (2005).

61. V. Muthye, C. D. Mackereth, J. B. Stewart, D. V. Lavrov, Large dataset of octocoral mitochondrial genomes provides new insights into mt-mutS evolution and function. *DNA Repair* **110**, 103273 (2022).
